# MHC class I evolution; from Northern pike to salmonids

**DOI:** 10.1186/s12862-020-01736-y

**Published:** 2021-01-21

**Authors:** U. Grimholt, M. Lukacs

**Affiliations:** Fish Research Group, Norwegian Veterinary Institute, Ullevaalsveien 68, 0454 Oslo, Norway

**Keywords:** MHC class I, Evolution, Whole genome duplication, Phylogeny, Northern pike, Salmonids

## Abstract

**Background:**

Salmonids are of major importance both as farmed and wild animals. With the changing environment comes changes in pathogenic pressures so understanding the immune system of all salmonid species is of essence. Major histocompatibility complex (MHC) genes are key players in the adaptive immune system signalling infection to responding T-cells populations. Classical MHC class I (MHCI) genes, defined by high polymorphism, broad expression patterns and peptide binding ability, have a key role in inducing immunity. In salmonids, the fourth whole genome duplication that occurred 94 million years ago has provided salmonids with duplicate MHCI regions, while Northern Pike, a basal sister clade to salmonids, represent a species which has not experienced this whole genome duplication.

**Results:**

Comparing the gene organization and evolution of MHC class I gene sequences in Northern pike versus salmonids displays a complex picture of how many of these genes evolved. Regional salmonid Ia and Ib Z lineage gene duplicates are not orthologs to the Northern pike Z lineage sequences. Instead, salmonids have experienced unique gene duplications in both duplicate regions as well as in the *Salmo* and *Oncorhynchus* branch. Species-specific gene duplications are even more pronounced for some L lineage genes.

**Conclusions:**

Although both Northern pike as well as salmonids have expanded their U and Z lineage genes, these gene duplications occurred separately in pike and in salmonids. However, the similarity between these duplications suggest the transposable machinery was present in a common ancestor. The salmonid MHCIa and MHCIb regions were formed during the 94 MYA since the split from pike and before the *Oncorhynchus* and *Salmo* branch separated. As seen in tetrapods, the non-classical U lineage genes are diversified duplicates of their classical counterpart. One MHCI lineage, the L lineage, experienced massive species-specific gene duplications after *Oncorhynchus* and *Salmo* split approximately 25 MYA. Based on what we currently know about L lineage genes, this large variation in number of L lineage genes also signals a large functional diversity in salmonids.

## Background

Salmonids comprise many species that are of major importance both as farmed and wild animals on many continents. Many stakeholders are affected when disease outbreaks caused by the many bacterial and viral pathogens occur. Although vaccines have been widely used to reduce disease outbreaks in fish farming, some pathogens still have a negative impact on the industry. Thus, understanding how the immune system handles pathogens and how protective immunity is achieved is important.

Major histocompatibility complex (MHC) molecules are involved in protection against invading pathogens. Most MHC class I (MHCI) molecules are composed of an alpha chain non-covalently linked to a beta2-microglobulin (b2m) molecule. Classical MHCI molecules are defined by their polymorphic content, their expression in most tissues and their ability to bind and present peptides to CD8+ T-cells. Here, the two extracellular alpha 1 and alpha 2 domains of the alpha chain are highly polymorphic and responsible for binding peptides from self and non-self proteins. The alpha 3 domain and b2m contribute with structural stability and CD8 binding. In humans the classical genes are denoted *HLA-A*, *HLA-B* and *HLA-C* and each gene has more than 3600 protein alleles registered in the IPD-HLA database (https://www.ebi.ac.uk/ipd/imgt/hla/stats.html). Non-classical MHCI molecules have more restricted expression patterns, lower levels of polymorphism and most have non-peptide ligands.

One major difference between the mammalian and teleost MHC is the regional organization. In teleosts, the classical MHCI and MHCII genes have separated with class I being linked to genes involved in peptide generation and transport while MHC class II genes reside elsewhere [1, 2]. In humans, the classical MHCI and MHCII genes reside within a 4 Mb region on chromosome 6 alongside genes involved in generating and transporting peptides such as the proteasome component beta genes PSMB8, PSMB9 and the antigen transporter TAP2 [3].

Salmonids experienced a whole genome duplication 94 million years ago where many of the duplicated regions are retained [4, 5]. In rainbow trout and Atlantic salmon this has resulted in a duplicate version of the entire MHCI region with the MHCIa region containing the classical MHCI UBA gene. The duplicate MHCIb region harbours several non-classical U lineage genes [6, 7].

Teleost fish are phylogenetically very distant from mammals and share no MHCI orthology with human MHCI lineages, although they both originated from a common ancestor 450 million years ago. The only lineage shared between the sarcopterygian and actinopterygian lineages is the teleost MHCI Z lineage that is also present in lungfish [8, 9]. In addition to the Z lineage, teleosts have five other MHCI lineages denoted U, L, S, P and H [8, 10, 11]. The U lineage is composed of both classical as well as non-classical peptide-binders. Most teleosts studied so far only have one to possibly three classical U lineage genes. Atlantic salmon and rainbow trout both have only one classical MHCI gene denoted UBA, while Medaka has two classical MHCI genes denoted UAA and UBA [6, 7, 12]. Zebrafish has varying haplotypes with one to three classical MHCI genes [13]. A species where evolution has created a different MHC system is Atlantic cod, which has expanded the MHCI lineage with 100 genes or more, potentially compensating for the lack of MHC class II molecules [14]. Number and polymorphic content of classical U lineage genes in other teleost and ray-finned species are currently not well defined.

Previous studies have shown that U lineage domains have different evolutionary histories with alpha 1 domain sequences segregating as distinct lineages shared between distantly related species [15–17]. Also alpha 2 domain sequences display some evolutionary conserved lineages, although this pattern is less pronounced than for the alpha 1 domain. Alpha 3 domains on the other hand, seem more structurally constrained potentially due to adaptation to species-specific b2m and CD8 association.

One additional MHCI lineage is a peptide-binder, i.e. the Z lineage, which we found to have a completely conserved peptide-binding motif in all studies ray-finned fishes [8]. These Z lineage genes reside in both the MHCIa and MHCIb regions in Atlantic salmon [8]. A complete conservation of the peptide binding residues suggest an intriguingly conserved, but yet undefined, function.

None of the four remaining teleost MHCI lineages have properties consistent with being peptide binders. The L lineage molecules most likely binds hydrophobic ligands, and can be traced back to spotted gar, a species that separated from teleosts before the teleost specific third whole genome duplication event (3WGD) [8]. Different Atlantic salmon L lineage genes were recently shown to vary in their response to pathogen stimulations [18], suggesting they have different roles in defence against pathogens.

The function of the remaining three teleost MHCI lineages is currently unknown. Both the P and H lineage can also be traced back to spotted gar and the P lineage has greatly expanded in species such as pufferfish [8]. Sequences from this H lineage show unprecedented deterioration of its extracellular domains, where teleosts have lost the alpha 3 domain as compared to their spotted gar ortholog. The alpha 1 and alpha 2 domains of teleost H lineage molecules is shorter in some species while the cytoplasmic tail has been conserved across divergent species [10]. The S lineage has only been identified in teleosts.

As mentioned above, salmonids experienced a whole genome duplication approximately 94 million years ago (MYA) [5] where many of the duplicated genes are retained. At least in Atlantic salmon, duplicated genes have taken on new functions rather than sub-functionalization [4]. Access to many new salmonid genomes now open for investigations on how the MHC genes and regions have evolved in this complex duplicated landscape. Northern pike represents a sister phylum to salmonids, that split from the salmonid lineage prior to the fourth whole genome duplication (4WGD) event [19]. Northern pike thus enables studies of how the 4WGD affected evolution of genes and gene duplicates in salmonids. Here, we made use of the available genomes of Northern pike and seven salmonid species to study how the 4WGD affected the evolution of MHCI.

## Results

**Fig. 1:**
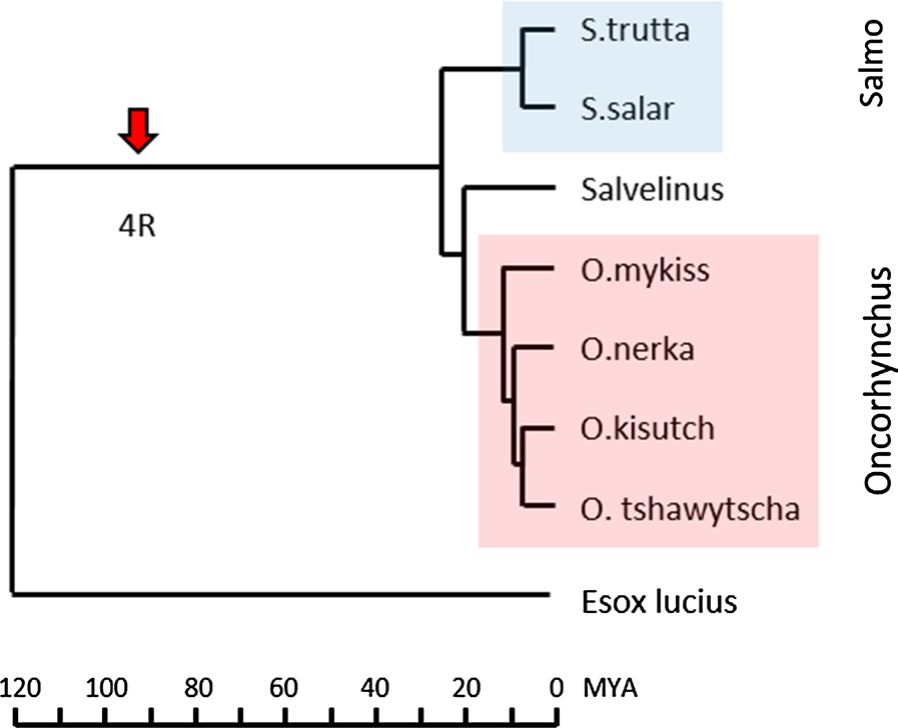
Phylogeny of *Salmonidae* and Northern pike. Phylogenetic relationship between included species. Dating of individual events are based on data from [48, 49]. *Salmo* and *Oncorhynchus* species are shown using a blue and red box respectively. The unique salmonid whole genome duplication event that occurred approximately 94 million years ago (MYA) [5] is shown using a red arrow

The results presented below are based on the NCBI genomes of the salmonids Atlantic salmon, brown trout, rainbow trout, sockeye salmon, coho salmon, chinook salmon and charr (see 'Materials' and 'Methods' for details). All genomes, apart from charr and Northern pike, originated from completely homozygous or so-called double haploid animals thus eliminating the added confusion of allelic gene variants. To understand the evolution of genes, the salmonid data are compared against results from the Northern pike genome, a species that is basal to salmonids, but lacks the 4WGD [20] (Fig. 1). Genomes from the three *Salmonidae* genomes Coregonus, *Hucho hucho* and *Thymallus thymallus* were not included in this study since they contained un-annotated or incomplete genomic regions, thus not enabling informative comparisons.

The origin of the NCBI *Salvelinus* genome, now annotated as *Salvelinus* in NCBI, may potentially be *Salvelinus malma malma* and not *Salvelinus alpinus* as presented in the original article [21, 22]. Using standardised nomenclature exemplified by Sasa for *Salmo salar* and Eslu for *Esox lucius,* we also used Saal for *Salvelinus alpinus* although it may be Sama. We also use *Oncorhynchus* for coho salmon, chinook salmon, sockeye salmon and rainbow trout while we use *Salmo* for Atlantic salmon and brown trout (Fig. 1).

Orthology between salmonid regions is a summary of data obtained from Christensen et al. and Sutherland et al. [21, 23] presented in Additional file 1. For brown trout, the linkage groups presented by Leitwein et al. [24] do not match the chromosome numbers in the NCBI genome, so regional orthology is currently based on blast match with region specific genes from other salmonids when this was informative.

We chose to define pseudogenes as those genes with internal stop codons and these genes have been given a − ps or ψ extension to the gene name. Partial genes have been given a -pt extension to separate them from remaining full-length bona fide gene sequences. The functional status of MHCI genes must await expression data from multiple tissues, multiple animals and diverse developmental stages.

## Evolution of salmonid MHCIa and MHCIb regions

Based on previous data we define the genomic region containing the classical UBA locus as the MHCIa region and the duplicate region containing non-classical genes as the MHCIb region [6, 7]. Genes residing within these two regions also have an − a or − b extension. All salmonid genomes analysed in this study contained well-defined and annotated duplicated MHCIa and MHCIb regions (Additional file 2). The Ia region, containing the UBA locus, was overall identical for all salmonid species with one few exceptions. Brown trout has a unique CD5-like gene in between the *SLC39A7a* and *RING2a_L* gene.

The duplicate MHCIb region was also almost identical in all analysed species. The *LHX9_L* gene found in Northern pike is present in all salmonid MHCIb regions with the exception of *Salvelinus*. All but *Salvelinus* and Northern pike also have a varying number of chitin synthase-like (CHS2) genes in between the RXRB and SLC39A7 genes. Chitin synthase is a well-known molecule in fungi and invertebrates, but the functional role in fish and amphibians need to be defined [25]. In chinook salmon there is a duplicate of the entire MHCIb region (Genbank NW_020128813), which could be an assembly artefact as the sequenced animal was a double haploid.

## Evolution of U lineage genes

**Fig. 2:**
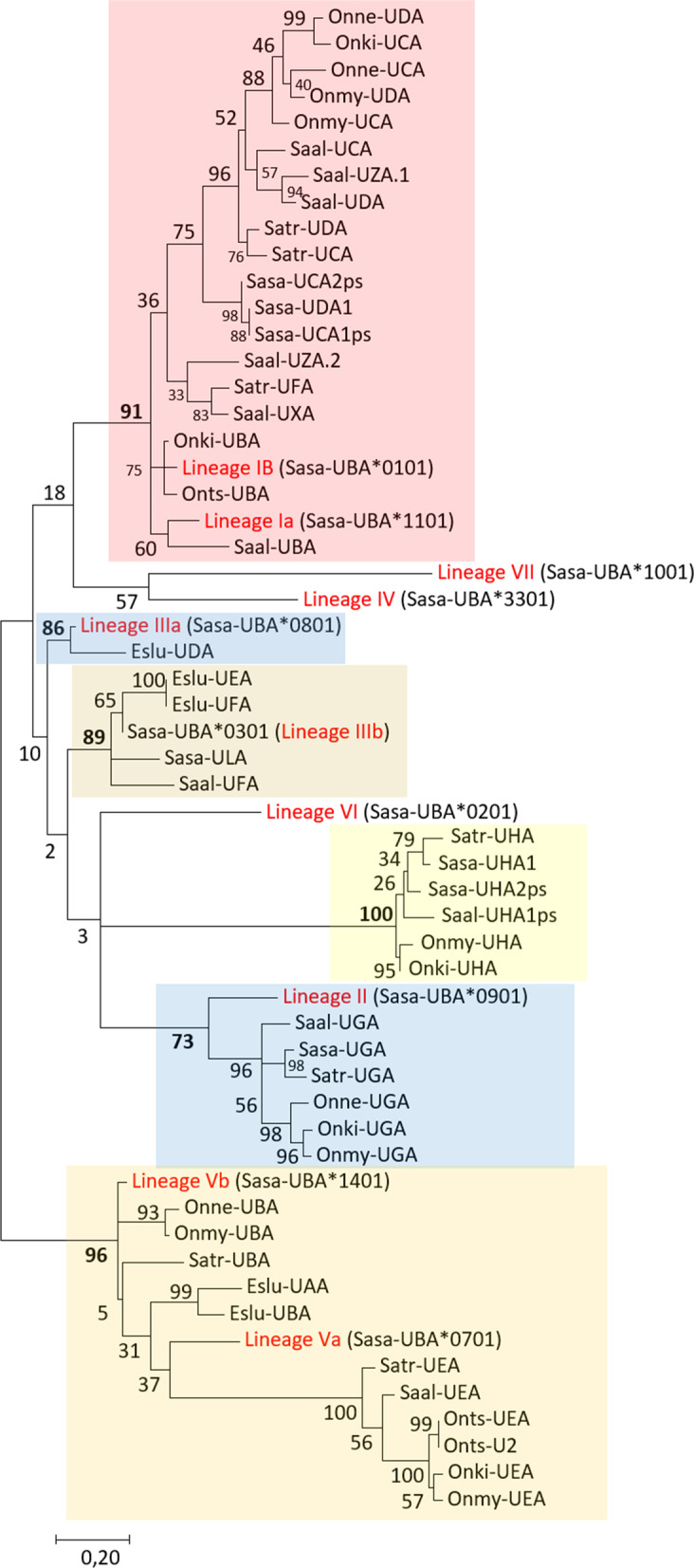
Phylogeny of deduced U lineage alpha 1 domain amino acid sequences. Lineages are shown using roman numbers as defined by Grimholt et al. [8]. Strongly supported clades are shown using coloured boxes. The tree with the highest log likelihood (− 3618,84) is shown. The percentage of trees in which the associated taxa clustered together is shown next to the branches. A discrete Gamma distribution was used to model evolutionary rate differences among sites (5 categories (+ G, parameter = 1,5774)). The tree is drawn to scale, with branch lengths measured in the number of substitutions per site. The analysis involved 59 amino acid sequences. There were a total of 85 positions in the final dataset. Atlantic salmon sequence references not present in Additional file 4 are as follows: UBA*0101 AAN75113, UBA*0201 AF504023, UBA*0301 AAN75116.1, UBA*0701 AAN75109, UBA*0801 AAN75115, UBA*0901 AAN75119, UBA*1001 AAN75118, *UBA1101* AF504017.1, UBA*1401 AAN75110, *UBA3301* DQ091795.1

Six Northern pike U lineage genes reside on chromosome 10 here defined as *Eslu-UAA* through *Eslu-UFA* (Additional files 2, 3, 4). Based on phylogeny, data indicate that there were three original genes where each of the three genes have duplicated into *Eslu-UAA* and *Eslu-UBA*, *Eslu-UCA* and *Eslu-UDA* and *Eslu-UEA* and *Eslu-UFA* (Fig. 2, Additional file 3). *Eslu-UCA* is only a partial sequence and may be a pseudogene. The polymorphic content of these genes remains undefined, but there is one EST and one TSA matching the Eslu-UAA/UBA genes (Genbank GH268323 and TSA GATF010284) and one EST originating from one of the *Eslu-UEA* or *Eslu-UFA* loci (EV373903). A seventh pike U lineage gene is located on an unplaced scaffold (*Eslu-UGA*, NW_022995044), and is a duplicate of the *Eslu-UDA* gene.

As previous studies have shown that the three extracellular alpha domains of U lineage sequences display different evolutionary patterns [8, 15–17], we made phylogenetic trees of both entire mature extracellular amino acid sequences as well as trees of individual alpha 1, alpha 2 and alpha 3 domain sequences to identify orthology (Fig. 2, Additional file 3). Phylogenies of alpha 1 domain sequences shared by distantly related teleost species, show that also non-classical genes share these lineages (Fig. 2) [8, 15–17]. Non-classical UEA gene sequences share the alpha 1 domain lineage Va, UGA gene sequences share the alpha 1 domain lineage II and most UCA and UDA gene sequences cluster with the alpha 1 domain lineage I. Also Northern pike U lineage genes share alpha 1 domain lineages with other teleosts. *Eslu-UAA* and *Eslu-UBA* alpha 1 domain sequences cluster with alpha 1 domain lineages Vb, *Eslu-UDA* clusters with lineage IIIa and *Eslu-UEA* and *Eslu-UFA* cluster with lineage IIIb sequences. In the alpha 2 domain analysis, all Northern pike sequences cluster together, although the bootstrap value is only 31% (Additional file 3). A similar clustering is also seen for all Northern pike alpha 3 domain sequences, with a higher bootstrap value.

Only one salmonid U lineage gene, UHA, resides outside of the two duplicated MHCIa and MHCIb regions (Table 1, Additional files 2 and 4). Sequences from this gene display strongly supported clusters in all phylogenies. Northern pike and sockeye salmon did not display any UHA gene sequences, but the remaining salmonids all have UHA lineage genes on one homeolog of Northern pike chr.16 (Additional file 1). Atlantic salmon and charr have regionally duplicated UHA lineage genes where at least the duplicate *Sasa-UHA2* gene is a pseudogene (Additional file 4). Although the two charr UHA gene sequences are incomplete, there is an expressed UHA1/2-like sequence in *Salvelinus malma* (Genbank AYG86905.1), suggesting at least one of these UHA loci are functional also in charr. Overall, UHA gene sequences are very different from other U lineage sequences (Fig. 2, Additional file 3), suggesting an ancient origin. However, we have not been able to find orthologs in any other teleost, so these genes may have evolved fast in salmonids.

**Table 1:** Number of MHCI lineage genes in salmonids and Northern pike

	U	Z	L	S	H	P
Northern pike (Eslu)	7 (2)	5	4	1	1	-
Atlantic salmon (Sasa)	9 (4)	7	13 (6)	6 (3)	2 (1)	(1)
Brown trout (Satr)	8 (1)	7 (1)	25 (8)	3 (2)	2 (1)	(1)
Rainbow trout (Onmy)	7 (1)	6 (1)	14 (3)	2 (1)	2	(1)
Chinook salmon (Onts)	8 (4)	7*	16 (8)	2 (1)	2	(1)
Coho salmon (Onki)	6 (1)	5	14 (3)	2 (1)	2	(1)
Sockeye salmon (Onne)	5 (1)	5	14 (8)	2 (1)	2	(1)
Charr (Saal)	11* (1)	4	13 (10)	2 (1)	2	(1)

Number of MHCI lineage genes in various salmonids. Partial genes in addition to pseudogenes are given in parenthesis. Star denotes species where there are additional genes on unplaced scaffolds that are most likely assembly artefacts (see Additional file 4)

Only Atlantic salmon has a duplicate annotated U lineage gene in the MHCIa region denoted ULA, a gene that lacks the transmembrane domain (Additional files 2, 3, 4) [26]. We know that the UBA loci from Atlantic salmon, rainbow trout, brown trout and sockeye salmon are classical MHCI loci with considerable polymorphism [15, 17, 27–30]. There are currently 48 Atlantic salmon and rainbow trout UBA alleles registered in the IPD-MHC database [31] while 31 and 34 alleles have been defined in brown trout and sockeye salmon. The polymorphic content of UBA loci from coho, chinook and charr remains undetermined.

MHC class I gene richness is most profound in the salmonid MHCIb regions, with brown trout and *Salvelinu*s having four U lineage genes surrounding the TAPBPb and PSMB8b genes (Additional files 2 and 4). Rainbow trout has three annotated U lineage genes in this region with an additional fourth *Onmy-UFA* pseudogene reported previously [7]. Previous studies have shown that rainbow trout and Atlantic salmon MHCIb regions contain non-classical MHC genes, displaying low polymorphism and more restricted expression patterns than their classical UBA counterparts [6, 7]. Sockeye, chinook and coho salmon all have two annotated U lineage genes in this region. This region then resembles the three original MHCI genes found on Northern pike chromosome chr.10.

*Salvelinus* has two additional unplaced scaffolds containing U lineage genes, all clustering with alpha 1 domain lineage I sequences (UXA, UZA1/2; Fig. 2, Additional files 3, 4). Their origin and location is unknown, but as the sequenced genome does not originate from a double haploid animal, they could be allelic variants of non-classical U lineage genes or assembly artefacts. Chinook salmon also has two additional U lineage genes residing on unplaced scaffolds here denoted Onts-U1 and Onts-U2. Onts-U1 is a partial gene sequence with sequence identity to *Onts-UCA*. Onts-U2 is a duplicate of the *Onts-UEA* gene sequence, and most likely represents an assembly artefact as the chinook salmon genome originates from a double haploid.

MHCIb regions also contain a unique UGA gene that is present in all analysed salmonids, located in between the SLC39A7b and RING2Ab genes (Additional file 2). Chinook salmon lacks an annotated UGA gene, although there are expressed chinook sequences supporting a functional UGA locus (e.g. GGDU01219126.1). The gene denoted UGA in Northern pike (Additional file 4) is not an ortholog to the salmonid UGA genes, so UGA is a gene duplication that translocated to the MHCIb region after salmonids split from pike. UGA lineage sequences show strongly supported clusters in alpha 1 and alpha 2 domain phylogenies, while the alpha 3 domain sequences are more dispersed (Additional file 3).

Based on location and phylogenetic clustering, the UEA gene existed in a primordial salmonid, but was then lost in Atlantic and sockeye salmon (Fig. 2, Additional files 2, 3, 4). All UEA alpha domain phylogenies show strongly supported clusters. Salmonid UCA and UDA gene sequences also form strongly supported clusters in the alpha 1 and alpha 2 domain sequence phylogenies, suggesting they originate from a salmonid ancestor. Duplications from a single primordial UC/DA gene to multiple UCA and UDA genes seem to have occurred individually in the *Oncorhynchus* and *Salmo* lineages based on the alpha 2 domain phylogenies, as well as in each individual species (Fig. 2, Additional file 3). The gene sequences defined as UFA in charr and brown trout do not cluster in phylogenies, so they represent within species gene duplications. However, the UFA pseudogene previously reported in rainbow trout, clusters with the UFA sequence from brown trout (data not shown), so this gene originated in a salmonid ancestor.

We have previously shown that the Atlantic salmon MHCIb region contains haplotypes with varying number of non-classical *Sasa-UCA* and *Sasa-UDA* genes [32]. One sequenced BAC had 30 Kb separating the UDA and UCA genes while another haplotype only had one UCA pseudogene (Genbank FJ969490). The Atlantic salmon genome contains an additional haplotype with 8 Mb separating the *Sasa-UCA* pseudogene from two additional UCA and UDA genes. Brown trout, the closest relative to Atlantic salmon, does not show this UCA/UDA gene duplication 10 Mb upstream suggesting this may be Atlantic salmon specific.

With the exception of *Salvelinus*, salmonids have a U lineage gene located approximately 10 Mb downstream of their UCA genes, a gene we here denoted UMA. All UMA genes contain internal stop codons or are partial gene sequences, suggesting they are nonfunctional. These regions do not contain the same genes as those surrounding the Atlantic salmon genome *Sasa-UDA* gene 8 Mb upstream of the major MHCIb region (Additional file 2). Nor do these regions resemble the UIA region found in Medaka, where there is approximately 14 Mb between the classical UAA/UBA genes and a UIA gene [16]. Thus, the salmonid UMA gene is a unique gene duplication that occurred early in the salmonid lineage.

## Z lineage evolution

In addition to the six U lineage genes, Northern pike also has five Z lineage genes on chr.10 (Table 1, Additional files 2, 4). In comparison, the salmonid MHCIa and Ib regions all have from two to four Z lineage genes per region. Due to the unique position of the *Salmo* ZAA gene residing in the MHCIa region, we chose to reserve this ZAA name to reflect a location in between the VHSVa induced protein and ATF6a. The remaining sequences are named ZBA through ZDA regardless of phylogenetic clustering. Of pike and salmonid Z lineage genes, only *Onmy-ZDAb* and *Satr-ZDAb* are defined as pseudogenes.

**Fig. 3:**
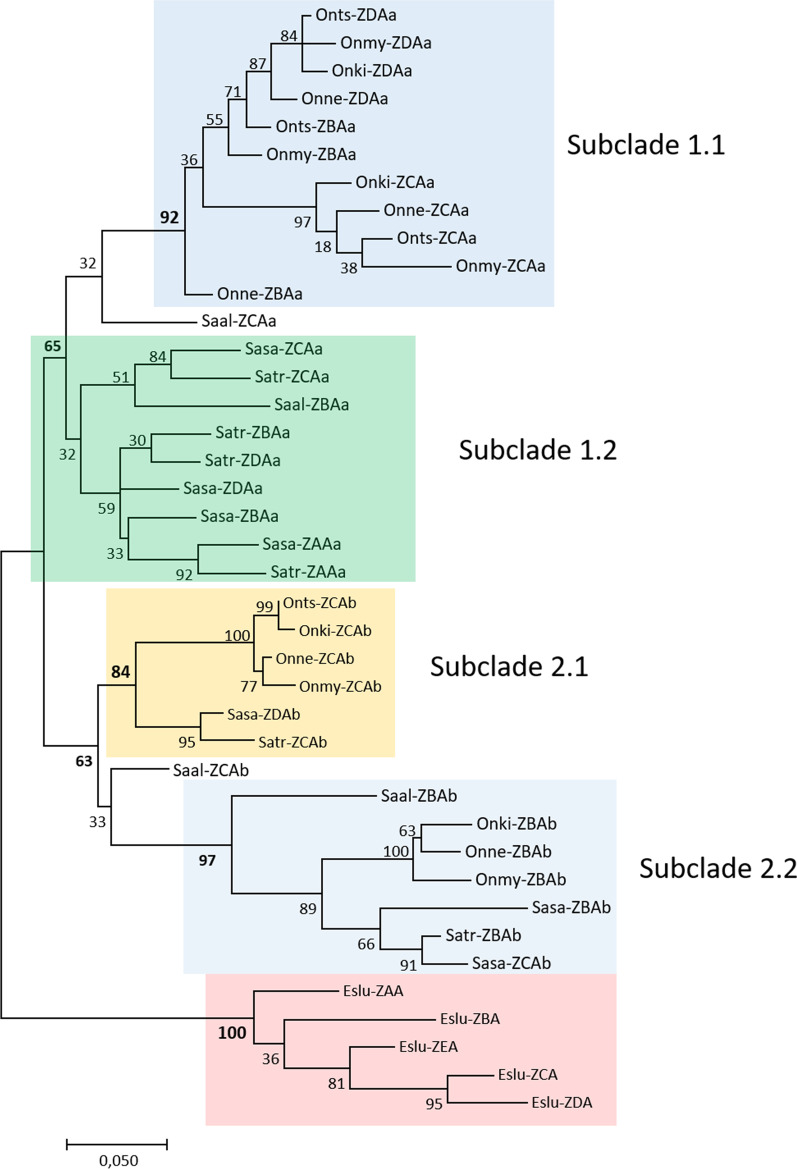
Phylogeny of deduced extracellular Z lineage amino acid sequences. The tree with the highest log likelihood (− 3771,17) is shown. The percentage of trees in which the associated taxa clustered together is shown next to the branches. A discrete Gamma distribution was used to model evolutionary rate differences among sites [5 categories (+ *G*, parameter = 0,3726)]. The tree is drawn to scale, with branch lengths measured in the number of substitutions per site. The analysis involved 40 amino acid sequences. There were a total of 282 positions in the final dataset. The different (sub)clades are shown using coloured boxes

Phylogenetic trees of the entire mature extracellular amino acid Z lineage sequences display two well-supported clades, each with two sub-clades. Surprisingly, all Northern pike Z lineage gene sequences cluster together with a strong bootstrap support, suggesting they are within species gene duplications (Fig. 3). Based on the two to four Z lineage gene duplicates identified in salmonid MHCIa and MHCIb regions (Additional file 2), one would have expected some orthology between pike and salmonid gene sequences.

The first clade (Fig. 3, clade 1) consists of MHCIa region sequences, while the second clade (Fig. 3, clade 2) consists of MHCIb region sequences, suggesting the Z lineage genes evolved independently in the MHCIa and MHCIb regions (Fig. 3, Additional file 2). Clade 1 gene sequences are further divided into two subclades, one containing *Oncorhynchus* gene sequences (subclade 1.1) and the other with *Salmo* gene sequences (subclade 1.2). Subclade 1.1 suggests that one original *Oncorhynchus* gene expanded to the three *Onmy-ZBAa*, *Onmy-ZCAa* and *Onmy-ZDAa* genes present in this region today where *Onmy-ZDAa* is a more recent duplicate of *Onmy-ZBAa*. Although not as strongly supported, *Salmo* Z lineage Ia genes within subclade 1.2 are also within region duplicates of one common ancestor. Here, the evolutionary process has repeated itself with the *Sasa-ZBAa* and *Sasa-ZDAa* genes are duplicates that split from *Sasa-ZCAa*. The unique *Salmo* ZAAa gene is also a more recent duplication of the *Sasa-ZBA* or *Sasa-ZDA* gene. Charr MHCIa Z lineage sequences show a dual clustering, with the *Saal-ZBAa* sequence clustering with *Oncorhynchus* while the *Saal-ZCAa* sequence clusters with *Salmo* ZCAa sequences.

Sequences originating from the MHCIb region split into two strongly supported subclusters (Fig. 3, subclades 2.1 and 2.2) and in this region *Oncorhynchus* and *Salmo* Z lineage genes share an evolutionary history. The subclade 2.1 contains ZCAb sequences while subclade 2.2 contains ZBAb sequences. The only exception is Atlantic salmon sequences where *Sasa-ZBAb* and *Sasa-ZCAb* represents a more recent gene duplication (Fig. 3). *Sasa-ZBAb* is the only soluble Z lineage molecule, lacking the transmembrane region [32].

## Evolution of L lineage genes

**Fig. 4:**
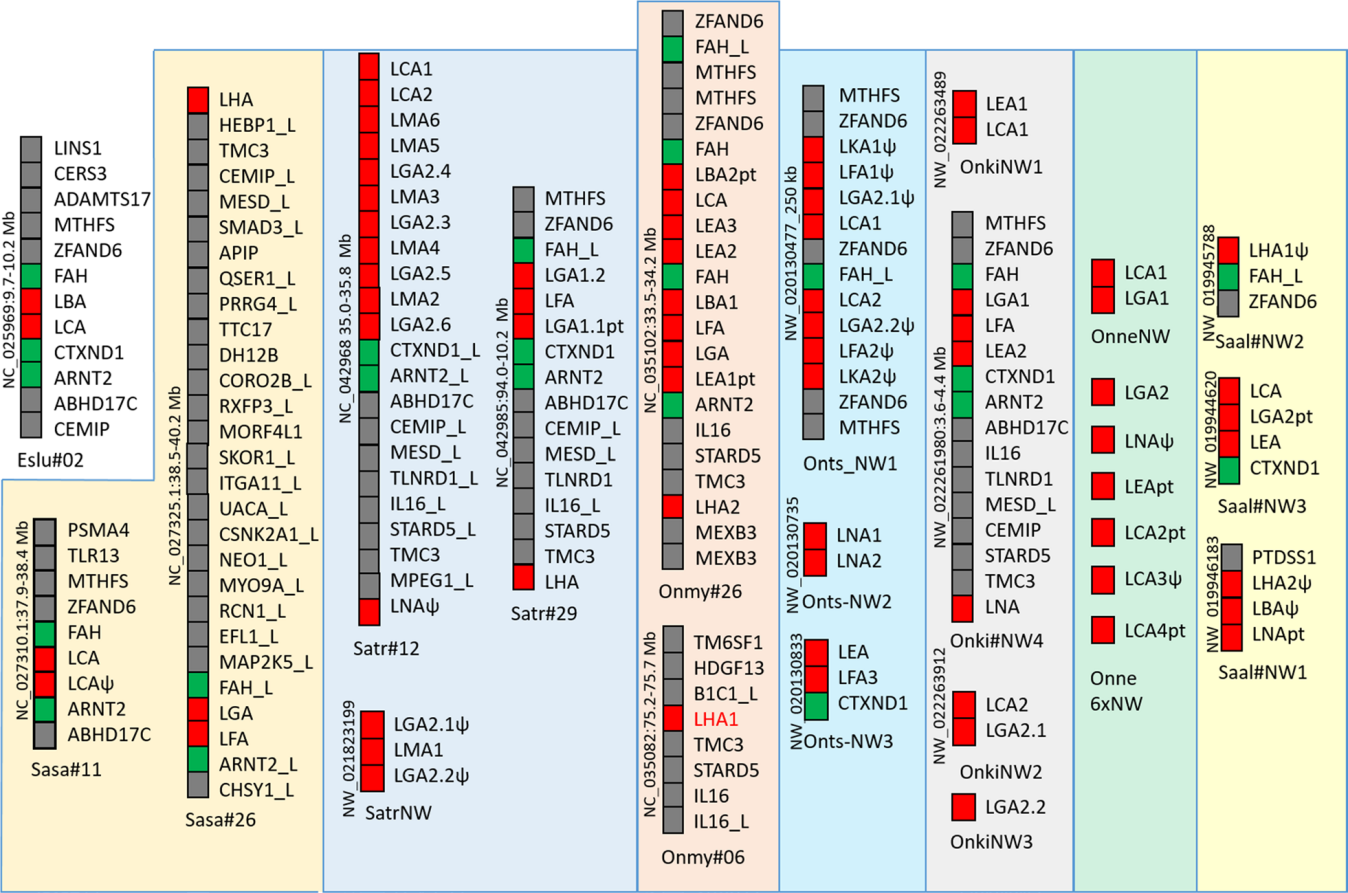
Comparison of L lineage regions from salmonids and Northern pike. Genomic regions containing L lineage genes clustering in phylogenetic analyses and based on regional orthology. Genes represented by boxes are colour shaded as follows: red boxes are L lineage genes, green boxes are flanking genes found in most regions and grey boxes are other genes. Additional colour shading is used for regions from each species. Regional location is shown on the side of each region and species and chromosome when available is shown below. Details of unplaced scaffolds can be found in Additional file 3. Atlantic salmon and rainbow trout genes are on homeolog chromosomes (see Additional file 1), orthology to brown trout chromosomes is undefined and regions from the remaining species are all unplaced scaffolds (NW), thus proving no informative on orthology. Pseudogenes are shown using ψ while partial genes are shown using a pt name extension. Many genes have the extension _L for _like as they need further phylogenetic and functional studies to warrant a definite gene name.

**Fig. 5:**
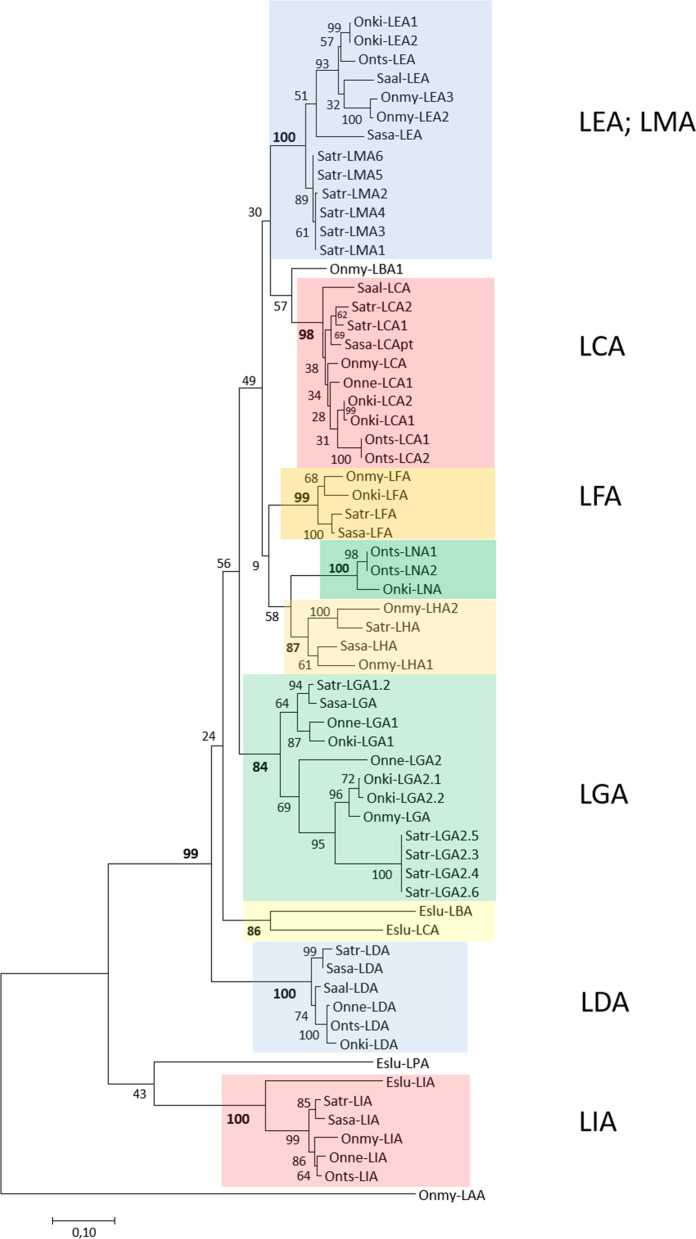
Phylogeny of deduced extracellular L lineage amino acid sequences. The tree with the highest log likelihood (− 6285,74) is shown. The percentage of trees in which the associated taxa clustered together is shown next to the branches. A discrete Gamma distribution was used to model evolutionary rate differences among sites [5 categories (+ G, parameter = 0,7617)]. The tree is drawn to scale, with branch lengths measured in the number of substitutions per site. There were a total of 269 positions in the final dataset. Strongly supported clusters are shown using colour shaded boxes

Northern pike has four L lineage genes dispersed on chr.2, 15 and 20 where salmonids have orthologs to the pike genes on chr.2 and chr.20 based on phylogeny and regional orthology (Table 1, Fig. 4, Additional files 1 and 4). Nomenclature is based on phylogenetic clustering with previously identified L lineage gene sequences [8, 11], as exemplified by the LGA gene sequences, which form a strongly supported phylogenetic cluster (Fig. 5). L lineage genes have exploded in salmonids ranging from 13 genes in charr to 25 genes in brown trout. Most charr L lineage genes are defined as pseudo or partial genes, but this needs verification by expressed sequences. The remaining species have 6–17 bona fide genes.

The previously published rainbow trout *Onmy-LAA* gene [11], is also found in salmonid species, whereas this gene was lost in Northern pike (Additional file 2). Fragments of this gene is found on Atlantic salmon homeolog chromosomes 13 and 15 and ortholog regions in the other salmonids (Additional files 1, 2, 4), flanked by ANKS1A and SARG genes. Only rainbow trout has a bona fide LAA gene, where the LAA genes from the other species are partial or pseudogenes. The *Onmy-LAA* sequence is quite distant from the remaining L lineage sequences and forms the base of the phylogenetic tree (Fig. 5).

Another older L lineage gene previously described in Atlantic salmon, LIA, [8] has orthologs in all species including Northern pike (Fig. 5, Additional files 1, 2, 4). LIA gene sequences are also quite old forming a strongly supported branch quite basal in the phylogenetic tree. Only the charr LIA gene is a pseudogene with an internal stop codon. This LIA gene is flanked by VWA8 and F5 in all species. Although salmonid LIA regions are ortholog to Northern pike chr.16, the pike LIA gene resides on chr.20, suggesting a translocation in a salmonid ancestor. The salmonid homeolog chromosome also hold L lineage genes in most species represented by the LLA and LJA genes, many being pseudogenes. Although not strongly supported, the Northern pike L lineage region on chr.15, here called *Eslu-LPA* clusters with the LIA gene sequences and is most likely a gene duplication specific for Northern pike. A similar unique gene duplication is seen for the Atlantic salmon *Sasa-LKA* gene with no ortholog region in other salmonids or Northern pike.

Salmonid LDA gene sequences represent another strongly supported clade, but also clusters with the remaining gene sequences from Northern pike and salmonids (Fig. 5, Additional files 2, 4). Salmonid LDA genes reside on an ortholog of pike chr.9 flanked by IRAK1BP1 and IL17RD genes (Fig. 4, Additional file 1). These genes are found on pike chr.17, without traces of the LDA gene (data not shown). Only located on one of the salmonid homeologs, the LDA gene most likely translocated to these salmonid regions after the 4WGD event.

Salmonid orthologs to the Northern pike chr.2 genes here defined as *Eslu-LBA* and *Eslu-LCA* have expanded a lot with brown trout being the most extreme with twelve L lineage genes on chr.12 (Fig. 4 and 5, Additional file 4). FAH and CTXND1/ARNT2 genes, flanking the two pike L lineage genes on chromosome 2, are also present in ortholog regions represented by Atlantic salmon chr.11 and chr.26 [23]. Most likely due to regional complexity, clustering genes from coho, chinook, sockeye and charr all reside on unplaced scaffolds. Gene expansions have occurred locally after the 4WGD. For instance, Atlantic salmon chr.11 with the two duplicate *Sasa-LCA* genes is a homeolog of rainbow trout chr.26 containing nine L lineage genes. Brown trout chr.12 and an unplaced chinook scaffold both display a similar L gene expansion with twelve and eight L lineage genes respectively. Most of the chinook genes on this unplaced scaffold are pseudogenes with internal stop codons while most of those on rainbow trout chr.26 are bona fide genes. Phylogenetically, also LEA/LMA genes as well as LFA genes reside in strongly supported clusters suggesting a shared evolutionary history for these sequence clades.

## Evolution of S, H and P lineage genes

S lineage genes have previously been described in many teleosts [8]. This gene is also present in Northern pike on chr.1 (Table 1, Additional files 2 and 3). Most salmonids have duplicate S lineage genes on both homeologs, where the SBA gene has been silenced in a primordial salmonid (Table 1, Additional files 2 and 4). Atlantic salmon has six S lineage genes residing on unplaced scaffolds where three of these six genes are partial gene sequences and may be pseudogenes. In a previous study, we sequenced a bacterial artificial chromosome (BAC) clone originating from chr.9, which contained one SAA gene in addition to the flanking VWA5 and AKT2 genes [32]. We did not find other BACs positive for the SAA probe, so potentially there are individual differences in the number of SAA genes in Atlantic salmon.

A fifth MHCI lineage described in teleosts is the P lineage, which has expanded to 24 genes in pufferfish [8]. Remnants of this P lineage is lacking in Northern pike while all salmonid P lineage genes have been silenced (Table 1, Additional files 2 and 4). Only one homeolog has remnants of this P lineage gene, suggesting it has been deleted in the duplicated region. The PAA gene is surrounded by PPP1R12A_like and Immunoglobulin light chain (Ig-L) genes. We previously found an IgL gene linked to a UIA gene in Medaka and to Z lineage genes in stickleback [8]. IgL genes are also found linked to the shark MHC region, suggesting it was present in the primordial MHC region [33].

We recently found a sixth MHC class I lineage in teleosts which we denoted the H lineage [10]. One HAA lineage gene is present in Northern pike and all salmonids studied here have HAA and HBA genes on homeologs to this pike HAA gene on chr.3 (Table 1, Additional files 1, 2, 4). All regions have TOX and PPP1R7 genes flanking the H lineage gene. The HAA genes seem functional in all species, while the HBA gene is a pseudogene at least in *Salmo* species. In coho and chinook, there are expressed reads matching the HBA gene (GGDU01537164.1, GDQG01022515.1), suggesting the homeolog HBA gene has retained a function in some species. The fact that H lineage sequences lack the alpha 3 domain, and has a cytoplasmic domain highly conserved also between distant teleost species, suggests that teleost MHCI may have a broader functional diversity than previously envisioned. Mammalian equivalents with such a molecular structure are the ULBP/RAET genes, which interact with the NKG2D receptor upon stress or infection [34]. If the salmonid H lineage molecules have a similar function remains to be determined.

## Discussion

All salmonids have single MHCIa UBA genes, defined as classical in brown trout, sockeye, Atlantic salmon and rainbow trout based on polymorphic content, peptide binding ability and for some broad expression patterns [15, 17, 27–30]. Although without functional evidence, we also expect these MHCIa genes in coho salmon, chinook salmon and charr to be classical genes. Similarly, we expect salmonid MHCIb region U lineage genes to be non-classical as shown in Atlantic salmon and rainbow trout [7, 32, 35].

In zebrafish, there are functional MHCI haplotypes with polymorphism in closely linked proteasome subunits PSMB8, PSMB13 as well as TAP2 [36]. Each haplotype contain one to three widely expressed U lineage genes [13], where polymorphic content and thus classical nature still need verification. Such functional haplotypes were not found in MHCIa and MHCIb regions of Atlantic salmon and rainbow trout [37]. In rainbow trout, the two allelic PSMB8 variants found in zebrafish are encoded by two different genes in the trout MHCIa region. Here, the *Onmy-PSMB8a* gene is a pseudogene while the *Onmy-PSMB8F* gene is functional. PSMB8F pseudogenes were also found in the duplicate Atlantic salmon MHCIa and MHCIb regions alongside functional PSMB8 genes [37]. However, there is a bona fide Atlantic salmon *Sasa-PSMB8F* sequence in Genbank (ACI66984.1), suggesting some Atlantic salmon haplotypes may have a functional variant of this gene. Neither pike nor other salmonids have an annotated PSMB8F gene in the MHCIa region, but charr has a PSMB8F gene on an unplaced scaffold (XP_023998549.1). Atlantic salmon and rainbow trout haplotypes did not display allelic variants of the PSMB13 and TAP2 gene either. Although we do not have data to support lack of functional haplotypes for the remaining salmonids, we postulate that functional haplotypes similar to those found in zebrafish are not operational in salmonids. The 4WGD providing duplicate MHCI regions with functional copies of tapasin, proteasome components and TAP2 may have replaced such haplotypes if they exist in Northern pike.

Evolutionary orthology between individual Northern pike and salmonid MHCI gene sequences is not apparent in our phylogenies. The seven pike U lineage genes occurred through duplications in pike after the split from salmonids. A similar gene expansion of U lineage genes in the MHCIb region has occurred in a salmonid ancestor, where a primordial UBA gene has duplicated and diversified into the non-classical genes found in the MHCIb regions today. Such a species-specific duplication of classical genes into diversified non-classical genes has also occurred in some tetrapod species [38, 39]. Sharing of alpha 1 domain lineages between classical and non-classical MHCI genes in addition to alpha 3 domain sequence conservation due to CD8 and b2m interaction adds to problems in reconstructing the evolutionary history of these salmonid genes.

Salmonid MHCIa and MHCIb Z lineage genes are not orthologs of the Northern pike Z lineage genes. Instead, the salmonid Z lineage genes have experienced unique gene duplications in the two duplicated regions sharing an evolutionary history in the MHCIb region, but evolving independently for *Oncorhynchus* and *Salmo* species in the MHCIa region. Potentially, transposable elements enabling these duplications were already present in Northern pike. As seen in other teleosts [8], all peptide anchoring residues are also conserved in salmonid Z lineage sequences (data not shown) suggesting they bind a similar or identical ligand as all other ray-finned fish Z lineage molecules.

Different evolutionary histories for MHCIa and MHCIb region genes are also reflected in their transcription patterns where Atlantic salmon MHCIa region genes dominated in gills while MHCIb genes had highest expression levels in gut [8]. Once we identify their common ligand, the functional advantage of having many Z lineage genes with different expression profiles will hopefully become apparent. In zebrafish, the Z lineage genes are not linked to the MHCI region on chr.19, but instead reside on chr.1 and chr.3 [8, 40]. Nine to twelve zebrafish Z lineage genes were found with diverse transcription patterns similar to that found in Atlantic salmon. Why teleosts need multiple Z lineage genes with diverse transcriptional patterns is unclear as they all seem to have one specific common ligand.

Both U and Z lineage genes comply with having peptide ligands so both lineages then rely on the peptide loading machinery to acquire these peptides. We recently found that Atlantic salmon have multiple genes for many of the components in this machinery originating from the second, third and fourth WGD [37]. It is tempting to speculate that specific combinations of the five protein disulfide-isomerase A3 (PDIA3) genes, six Tapasin (TAPBP) and tapasin-like (TAPBP-L) genes in addition to duplicate immunoproteasome components most likely provide peptides to classical U lineage genes. While other gene combinations play a role in providing peptides for non-classical U lineage genes and yet other combinations are important for peptide loading of Z lineage genes.

The L lineage genes have exploded in some salmonids with brown trout being the most extreme with 25 L lineage genes. The other salmonids have between three and eleven bona fide L lineage genes. A structural investigation of L lineage sequences found them to be able to bind quite hydrophobic structures, possibly analogue to mammalian CD1 molecules [8]. Our understanding of the L gene function has since advanced with the study by Edholm et al. [18] showing that L lineage genes display different responses upon stimulation. Six Atlantic salmon L lineage genes were included in their study where *Sasa-LIA* responded to a single-stranded RNA virus but not when challenged with a bacteria. *Sasa-LIA* and *Sasa-LGA* both responded to stimulation by type I interferon A, while *Sasa-LHA* did not. Instead, *Sasa-LHA* responded to a variety of viral and bacterial TLR ligands. These results show that salmonid L lineage genes have acquired a variety of functional roles in protection against pathogens. The large span in number of L lineage genes could reflect habitat differences for instance between fresh water and anadromous species, but unfortunately there is no information on the origin of the sequenced brown trout specimen and also uncertainty regarding the charr specimen. Future studies into number of expressed genes and their function are needed to clarify the biological role of L lineage genes in salmonids where brown trout and charr represent the two extremes.

## Conclusion

Although both Northern pike as well as salmonids have expanded their U and Z lineage genes, these gene duplications have occurred separately in pike and in a salmonid ancestor. However, the similarity between these duplications suggest the transposable machinery was present in a common ancestor. The salmonid MHCIa and MHCIb regions evolved during the 94 MYA since the split from pike and before the *Oncorhynchus* and *Salmo* branch separated. As seen in tetrapods, the non-classical U lineage genes are diversified duplicates of their classical counterpart. One MHCI lineage, the L lineage, experienced massive species-specific gene duplications after *Oncorhynchus* and *Salmo* split approximately 25 MYA. Based on what we currently know about L lineage genes, this diversity holds promise for yet undiscovered MHCI functions in salmonids.

## Methods

### Materials

Genomes used in this study are as follows: *Salvelinus alpinus/malma* GCA_002910315.2 (charr; [21]), *Salmo trutta* GCA_901001165.1 (brown trout, https://www.ncbi.nlm.nih.gov/assembly/GCF_901001165.1/), *Oncorhynchus nerka* GCA_006149115.1 (sockeye salmon; https://www.ncbi.nlm.nih.gov/assembly/GCF_006149115.1/), *Oncorhynchus tshawytscha* GCA_002872995.1 (chinook salmon [41]), *Oncorhynchus kisutch* GCA_002021735.2 (coho salmon; https://www.ncbi.nlm.nih.gov/assembly/GCF_002021735.2), *Oncorhynchus mykiss* GCA_002163495.1 (rainbow trout; [42]), *Salmo salar* GCA_000233375.4 (Atlantic salmon, [4]), and *Esox Lucius* GCA_004634155.1 (Northern pike; [20]).

### Data mining

Genome searches were performed using previously identified Atlantic salmon MHC gene sequences [8, 10, 32] and tblastn against annotated salmonid genomes available in NCBI. Genomic regions identified through these searches were screened for annotated genes. Some additional unannotated genes were also identified using tblastn search.

### Sequence alignments and phylogenies

Amino acid sequences were aligned using ClustalX [43] with manual corrections for some predicted sequences. Individual domain sequences used in phylogenies were extracted using Jalview [44]. All evolutionary analyses were conducted in MEGA7 [45]. The evolutionary history of selected amino acid sequences was inferred by using the Maximum Likelihood method based on the JTT matrix-based model [46]. Additional phylogenetic trees were also tested using the Neighbor-Joining method [47] (data not shown). The percentage of trees in which the associated taxa clustered together are shown next to the branches. Initial trees for the heuristic search were obtained automatically by applying Neighbor-Join and BioNJ algorithms to a matrix of pairwise distances estimated using a JTT model, and then selecting the topology with superior log likelihood value. The trees are drawn to scale, with branch lengths measured in the number of substitutions per site. All positions with less than 95% site coverage were eliminated. That is, fewer than 5% alignment gaps, missing data, and ambiguous bases were allowed at any position.

## Supplementary information

**Additional file 1:** Chromosomal orthology.

**Additional file 2:** Compared MHCI regions.

**Additional file 3:** Phylogeny of U lineage sequences.

**Additional file 4:** Deduced MHCI amino acid sequences.

## Data Availability

All data supporting the conclusions of this article are referred to or included within the article and its additional files.

## Abbreviations

MHCI: Major histocompatibility complex
B2m: Beta2-microglobulin
PSMB: Proteasome 20S subunit beta
IgL: Immunoglobulin light chain
4WGD: Unique salmonid fourth whole genome duplication
TAP: Transporter antigen peptide 2
Mb: Megabase
MYA: Million years ago
NK: Natural killer
BAC: Bacterial artificial chromosome
Eslu: *Esox lucius*
Sasa: *Salmo salar*
Onmy: *Oncorhynchus mykiss*
Onts: *Oncorhynchus tshawytscha*
Onne: *Oncorhynchus nerka*
Onki: *Oncorhynchus kisutch*
Saal: *Salvelinus alpinus/malma*

## References

[1] Bingulac-Popovic J, Figueroa F, Sato A, Talbot WS, Johnson SL, Gates M, Postlethwait JH, Klein J (1997). Mapping of mhc class I and class II regions to different linkage groups in the zebrafish, *Danio rerio*. Immunogenetics.

[2] Grimholt U (2016). MHC and evolution in teleosts. Biology (Basel).

[3] Klein J (1986). The natural history of the major histocompatibility complex.

[4] Lien S, Koop BF, Sandve SR, Miller JR, Kent MP, Nome T, Hvidsten TR, Leong JS, Minkley DR, Zimin A (2016). The Atlantic salmon genome provides insights into rediploidization. Nature.

[5] Macqueen DJ, Johnston IA (2014). A well-constrained estimate for the timing of the salmonid whole genome duplication reveals major decoupling from species diversification. Proc R Soc B.

[6] Lukacs MF, Harstad H, Grimholt U, Beetz-Sargent M, Cooper GA, Reid L, Bakke HG, Phillips RB, Miller KM, Davidson WS (2007). Genomic organization of duplicated major histocompatibility complex class I regions in Atlantic salmon (*Salmo salar*). BMC Genomics.

[7] Shiina T, Dijkstra JM, Shimizu S, Watanabe A, Yanagiya K, Kiryu I, Fujiwara A, Nishida-Umehara C, Kaba Y, Hirono I (2005). Interchromosomal duplication of major histocompatibility complex class I regions in rainbow trout (*Oncorhynchus mykiss*), a species with a presumably recent tetraploid ancestry. Immunogenetics.

[8] Grimholt U, Tsukamoto K, Azuma T, Leong J, Koop BF, Dijkstra JM (2015). A comprehensive analysis of teleost MHC class I sequences. BMC Evol Biol.

[9] Stet RJ, Kruiswijk CP, Dixon B (2003). Major histocompatibility lineages and immune gene function in teleost fishes: the road not taken. Crit Rev Immunol.

[10] Grimholt U, Tsukamoto K, Hashimoto K, Dijkstra JM (2019). Discovery of a novel MHC class I lineage in teleost fish which shows unprecedented levels of ectodomain deterioration while possessing an impressive cytoplasmic tail motif. Cells.

[11] Dijkstra JM, Katagiri T, Hosomichi K, Yanagiya K, Inoko H, Ototake M, Aoki T, Hashimoto K, Shiina T (2007). A third broad lineage of major histocompatibility complex (MHC) class I in teleost fish; MHC class II linkage and processed genes. Immunogenetics.

[12] Nonaka MI, Nonaka M (2010). Evolutionary analysis of two classical MHC class I loci of the medaka fish, *Oryzias latipes*: haplotype-specific genomic diversity, locus-specific polymorphisms, and interlocus homogenization. Immunogenetics.

[13] McConnell SC, Restaino AC, de Jong JL (2014). Multiple divergent haplotypes express completely distinct sets of class I MHC genes in zebrafish. Immunogenetics.

[14] Star B, Nederbragt AJ, Jentoft S, Grimholt U, Malmstrom M, Gregers TF, Rounge TB, Paulsen J, Solbakken MH, Sharma A (2011). The genome sequence of Atlantic cod reveals a unique immune system. Nature.

[15] Aoyagi K, Dijkstra JM, Xia C, Denda I, Ototake M, Hashimoto K, Nakanishi T (2002). Classical MHC class I genes composed of highly divergent sequence lineages share a single locus in rainbow trout (*Oncorhynchus mykiss*). J Immunol.

[16] Nonaka MI, Aizawa K, Mitani H, Bannai HP, Nonaka M (2011). Retained orthologous relationships of the MHC Class I genes during euteleost evolution. Mol Biol Evol.

[17] Shum BP, Guethlein L, Flodin LR, Adkison MA, Hedrick RP, Nehring RB, Stet RJ, Secombes C, Parham P (2001). Modes of salmonid MHC class I and II evolution differ from the primate paradigm. J Immunol.

[18] Svenning S, Gondek-Wyrozemska AT, van der Wal YA, Robertsen B, Jensen I, Jorgensen JB, Edholm ES (2019). Microbial danger signals control transcriptional induction of distinct MHC class I L lineage genes in Atlantic Salmon. Front Immunol.

[19] Rogers SL, Gobel TW, Viertlboeck BC, Milne S, Beck S, Kaufman J (2005). Characterization of the chicken C-type lectin-like receptors B-NK and B-lec suggests that the NK complex and the MHC share a common ancestral region. J Immunol.

[20] Rondeau EB, Minkley DR, Leong JS, Messmer AM, Jantzen JR, von Schalburg KR, Lemon C, Bird NH, Koop BF (2014). The genome and linkage map of the northern pike (*Esox lucius*): conserved synteny revealed between the salmonid sister group and the Neoteleostei. PLoS ONE.

[21] Christensen KA, Rondeau EB, Minkley DR, Leong JS, Nugent CM, Danzmann RG, Ferguson MM, Stadnik A, Devlin RH, Muzzerall R (2018). The Arctic charr (*Salvelinus alpinus*) genome and transcriptome assembly. PLoS ONE.

[22] Shedko S. Assembly ASM291031v2 (Genbank: GCA_002910315.2) identified as assembly of the Northern Dolly Varden (*Salvelinus malma malma*) genome, and not the Arctic char (*S. alpinus*) genome. *arXivorg*. (2019)

[23] Sutherland BJG, Gosselin T, Normandeau E, Lamothe M, Isabel N, Audet C, Bernatchez L (2016). Salmonid chromosome evolution as revealed by a novel method for comparing RADseq linkage maps. Genome Biol Evol.

[24] Leitwein M, Guinand B, Pouzadoux J, Desmarais E, Berrebi P, Gagnaire PA (2017). A dense brown trout (*Salmo trutta*) linkage map reveals recent chromosomal rearrangements in the Salmo genus and the impact of selection on linked neutral diversity. G3 (Bethesda).

[25] Tang WJ, Fernandez J, Sohn JJ, Amemiya CT (2015). Chitin is endogenously produced in vertebrates. Curr Biol.

[26] Miller KM, Li S, Ming TJ, Kaukinen KH, Schulze AD (2006). The salmonid MHC class I: more ancient loci uncovered. Immunogenetics.

[27] Grimholt U, Larsen S, Nordmo R, Midtlyng P, Kjoeglum S, Storset A, Saebo S, Stet RJ (2003). MHC polymorphism and disease resistance in Atlantic salmon (*Salmo salar*); facing pathogens with single expressed major histocompatibility class I and class II loci. Immunogenetics.

[28] Kiryu I, Dijkstra JM, Sarder RI, Fujiwara A, Yoshiura Y, Ototake M (2005). New MHC class Ia domain lineages in rainbow trout (*Oncorhynchus mykiss*) which are shared with other fish species. Fish Shellfish Immunol.

[29] O'Farrell B, Benzie JA, McGinnity P, de Eyto E, Dillane E, Coughlan J, Cross TF (2013). Selection and phylogenetics of salmonid MHC class I: wild brown trout (*Salmo trutta*) differ from a non-native introduced strain. PLoS ONE.

[30] McClelland EK, Ming TJ, Tabata A, Miller KM (2011). Sequence analysis of MHC class I alpha2 from sockeye salmon (*Oncorhynchus nerka*). Fish Shellfish Immunol.

[31] Maccari G, Robinson J, Bontrop RE, Otting N, de Groot NG, Ho CS, Ballingall KT, Marsh SGE, Hammond JA (2018). IPD-MHC: nomenclature requirements for the non-human major histocompatibility complex in the next-generation sequencing era. Immunogenetics.

[32] Lukacs MF, Harstad H, Bakke HG, Beetz-Sargent M, McKinnel L, Lubieniecki KP, Koop BF, Grimholt U (2010). Comprehensive analysis of MHC class I genes from the U-, S-, and Z-lineages in Atlantic salmon. BMC Genomics.

[33] Ohta Y, Kasahara M, O'Connor TD, Flajnik MF (2019). Inferring the "Primordial Immune Complex": origins of MHC class I and antigen receptors revealed by comparative genomics. J Immunol.

[34] Bacon L, Eagle RA, Meyer M, Easom N, Young NT, Trowsdale J (2004). Two human ULBP/RAET1 molecules with transmembrane regions are ligands for NKG2D. J Immunol.

[35] Dijkstra JM, Kiryu I, Yoshiura Y, Kumanovics A, Kohara M, Hayashi N, Ototake M (2006). Polymorphism of two very similar MHC class Ib loci in rainbow trout (*Oncorhynchus mykiss*). Immunogenetics.

[36] McConnell SC, Hernandez KM, Wcisel DJ, Kettleborough RN, Stemple DL, Yoder JA, Andrade J, de Jong JL (2016). Alternative haplotypes of antigen processing genes in zebrafish diverged early in vertebrate evolution. Proc Natl Acad Sci USA.

[37] Grimholt U (2018). Whole genome duplications have provided teleosts with many roads to peptide loaded MHC class I molecules. BMC Evol Biol.

[38] Dijkstra JM, Yamaguchi T, Grimholt U (2018). Conservation of sequence motifs suggests that the nonclassical MHC class I lineages CD1/PROCR and UT were established before the emergence of tetrapod species. Immunogenetics.

[39] Hughes AL, Nei M (1989). Evolution of the major histocompatibility complex: independent origin of nonclassical class I genes in different groups of mammals. Mol Biol Evol.

[40] Dirscherl H, Yoder JA (2013). Characterization of the Z lineage major histocompatability complex class I genes in zebrafish. Immunogenetics.

[41] Christensen KA, Leong JS, Sakhrani D, Biagi CA, Minkley DR, Withler RE, Rondeau EB, Koop BF, Devlin RH (2018). Chinook salmon (*Oncorhynchus tshawytscha*) genome and transcriptome. PLoS ONE.

[42] Berthelot C, Brunet F, Chalopin D, Juanchich A, Bernard M, Noel B, Bento P, Da Silva C, Labadie K, Alberti A (2014). The rainbow trout genome provides novel insights into evolution after whole-genome duplication in vertebrates. Nat Commun.

[43] Larkin MA, Blackshields G, Brown NP, Chenna R, McGettigan PA, McWilliam H, Valentin F, Wallace IM, Wilm A, Lopez R, et al. Clustal W and Clustal X version 2.0. Bioinformatics 2007;23(21):2947–48. 10.1093/bioinformatics/btm404 17846036

[44] Waterhouse AM, Procter JB, Martin DM, Clamp M, Barton GJ (2009). Jalview Version 2-a multiple sequence alignment editor and analysis workbench. Bioinformatics.

[45] Kumar S, Stecher G, Tamura K. MEGA7: molecular evolutionary genetics analysis version 7.0 for bigger datasets. Mol Biol Evol. 2016;33(7):1870–74. 10.1093/molbev/msw054 PMC8210823 27004904

[46] Jones DT, Taylor WR, Thornton JM (1992). The rapid generation of mutation data matrices from protein sequences. Comput Appl Biosci.

[47] Saitou N, Nei M (1987). The neighbor-joining method: a new method for reconstructing phylogenetic trees. Mol Biol Evol.

[48] Crete-Lafreniere A, Weir LK, Bernatchez L (2012). Framing the Salmonidae family phylogenetic portrait: a more complete picture from increased taxon sampling. PLoS ONE.

[49] Macqueen DJ, Primmer CR, Houston RD, Nowak BF, Bernatchez L, Bergseth S, Davidson WS, Gallardo-Escarate C, Goldammer T, Guiguen Y (2017). Functional Annotation of All Salmonid Genomes (FAASG): an international initiative supporting future salmonid research, conservation and aquaculture. BMC Genomics.

