# Supplementary material for: MHC class I evolution; from Northern pike to salmonids
Source: BMC Ecol Evol. 2021 Jan 21;21:3. doi: 10.1186/s12862-020-01736-y (PMC7853315; doi:10.1186/s12862-020-01736-y)
Supplement: Supplementary file 1 — Additional file 1: Chromosomal orthology. [file 12862_2020_1736_MOESM1_ESM.pdf]

Additional File 1 (AF1). Chromosomal orthology between salmonids and Northern pike.

Pike (Eslu)	A. Salmon (Sasa)	B. trout (Satr)	R. Trout (Onmy)	Chinook (Onts)	Coho (Onki)	Sockeye (Onne)	Charr (Saal)	Gene
1.1	20b	26	27	13q	15b	11a	22	SBA
1.2	9c	13	24	14q	18a	14b	4p	SAA
2.1	26	12*	6b	4q	3b	27a	15b	LFA-LHA
2.2	11a	29*	26	12q	8b	28a	10	LBA-LCA
3.1	14a	21	8b	10q	30	29	32	HAA
3.2	3a	31	28	28	27	20b	19	HBA
8.1	23	nd	4	1p	10a	17	13b	
8.2	10a	nd	5	5q	13a	24a	16	*Sasa-LKA*
9.1	2b	nd	13a	32	20b	21a	21	
9.2	12a	14	17b	2q	1b	15b	1a	LDA
10.1	27	34	18b	13p	17a	4a	31	UBA, ULA, Z
10.2	14b	36	14a	31	14b	7a	30	UCA, UDA, UMA, Z
12.1	13a	30*	16b	22	24	20a	17	LAA1
12.2	15b	28*	9b	16q	17b	7b	7	LAA2
15.1	9a	nd	25b	8p	14a	12a	4q	*Eslu-LPA*
15.2	1a	nd	19b	11q	7b	18a	9	*Eslu-LPA*
16.1	21	20	22	26	26	1	36	UHA,LIA
16.2	25	24	3b	3q	2b	3a	2	LLA,LJA
20.1	5b	nd	2a	23	13b	14a	6.1	*Eslu-LIA*
20.2	2a	nd	3a	3p	2a	3b	35	
24.1	7a	8	21b	15q	9b	8a	3b	PAA
24.2	18b	40	9a	10p	16a	12b	37	

Orthology between relevant Northern pike and salmonid regions is a summary of data obtained from main text references Christensen et al., 2018 and Sutherland et al., 2016. For Brown trout, regional orthology is based on blast match with region specific genes from other salmonids where * denotes undefined orthology. Species abbreviations are Eslu (*Esox Lucius),* Sasa (*Salmo salar),* Onmy (*Oncorhynchus mykiss),* Onts (*Oncorhynchus tshawytscha),* Onne (*Oncorhynchus nerka),* Onki (*Oncorhynchus kisutch),* Saal (*Salvelinus alpinus/malma).*
