# Supplementary material for: MHC class I evolution; from Northern pike to salmonids
Source: BMC Ecol Evol. 2021 Jan 21;21:3. doi: 10.1186/s12862-020-01736-y (PMC7853315; doi:10.1186/s12862-020-01736-y)

Additional file 2 (AF2): Comparison of genomic MHCI regions from selected Salmonids and Northern pike

AF	Region	Page
AF2.1	MHC class Ia regions	2
AF2.2	MHC class Ib regions	3
AF2.3	Extended salmonid MHC Ib regions	4
AF2.4	UHA regions	5
AF2.5	L lineage regions	6-9
AF2.6	S lineage regions	10
AF2.7	H lineage regions	11
AF2.8	P lineage regions	12

Comparison of genomic regions of MHC class I lineage genes in Salmonids and Northern pike. Genes are depicted as grey boxes apart from MHC class lineage genes are shown as red boxes and in red font. Chromosomal location or unplaced scaffold names are shown below and on the left hand side of each. Scaffolds with MHCI genes only are not shown (see Additional file 4). Typical regional genes are shown using green boxes. Phylogenetic clustering of MHCIa and MHCIb U and Z lineage genes is shown using colored boxes covering the gene names. Arrow in the MHCIb regions indicate direction of the extended MHCIb region shown on page 3 and 4. Large regional gaps are shown using solid black line where gap distance is shown in megabases (Mb). Definite pseudogenes are shown in gene name with a ps extension. Many gene names also have an _L extension for _like as functional verification is needed to define them as orthologs of mammalian molecules. Page 10 * : The single Atlantic salmon S gene region located on Chr. 09 originates from main text reference Lukacs et al.2010, while the six S lineage gene sequences identified in the Atlantic salmon genome all reside on small scaffolds with MHC genes only.

AF2.1. MHC class Ia regions

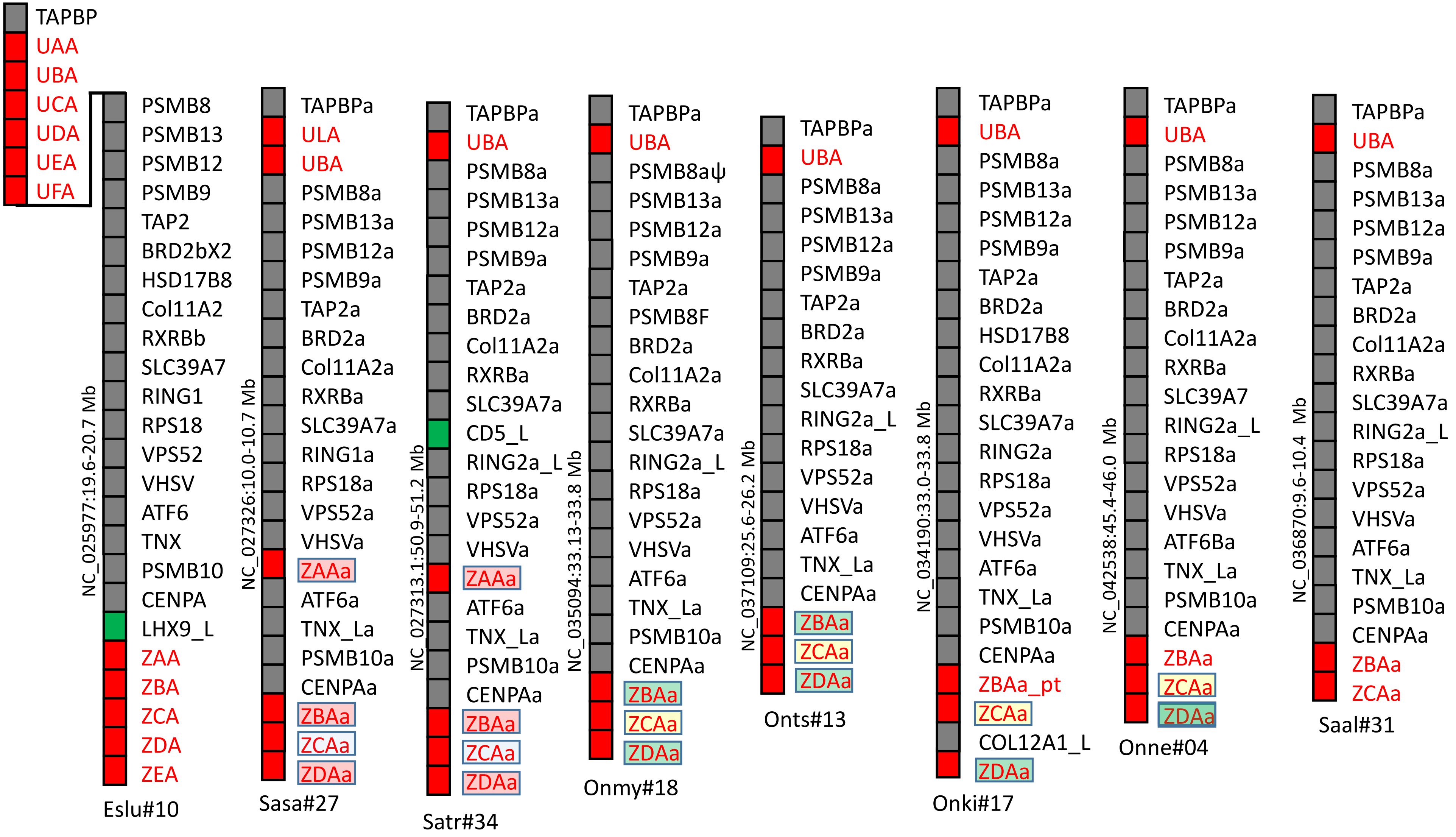

AF2.2. MHC class Ib regions

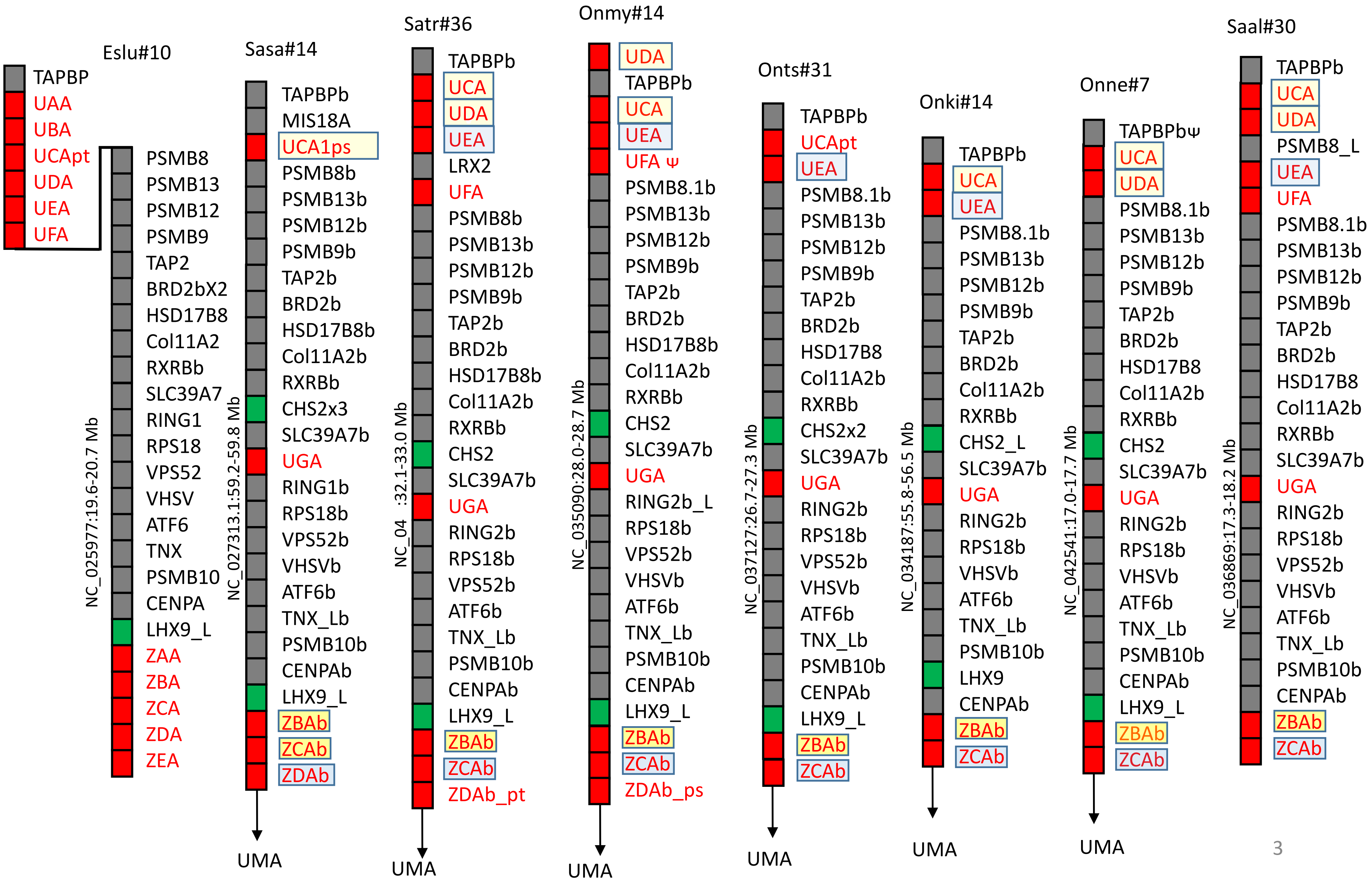

AF2.3. Extended salmonid MHC class Ib regions

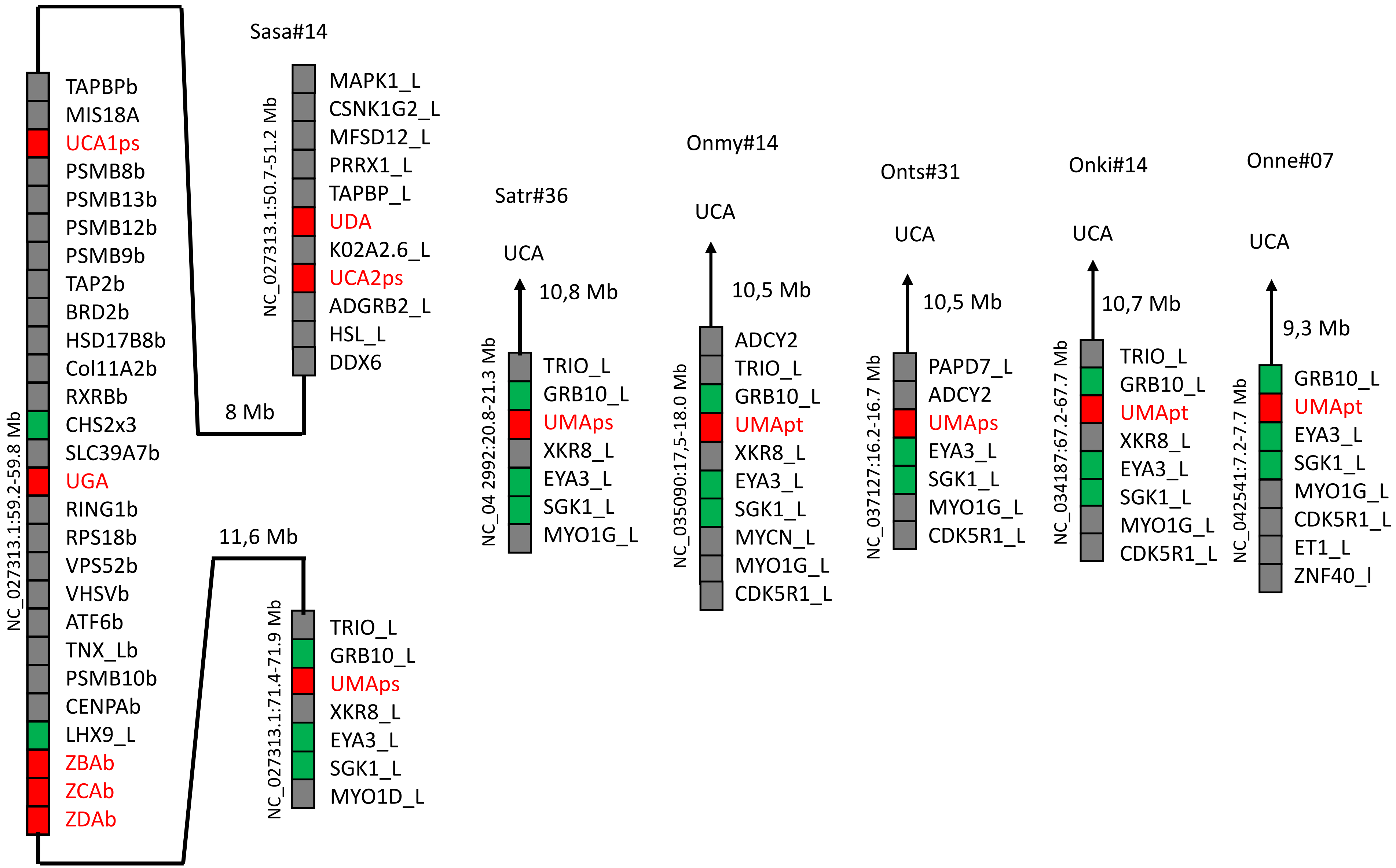

AF2.4. UHA lineage regions

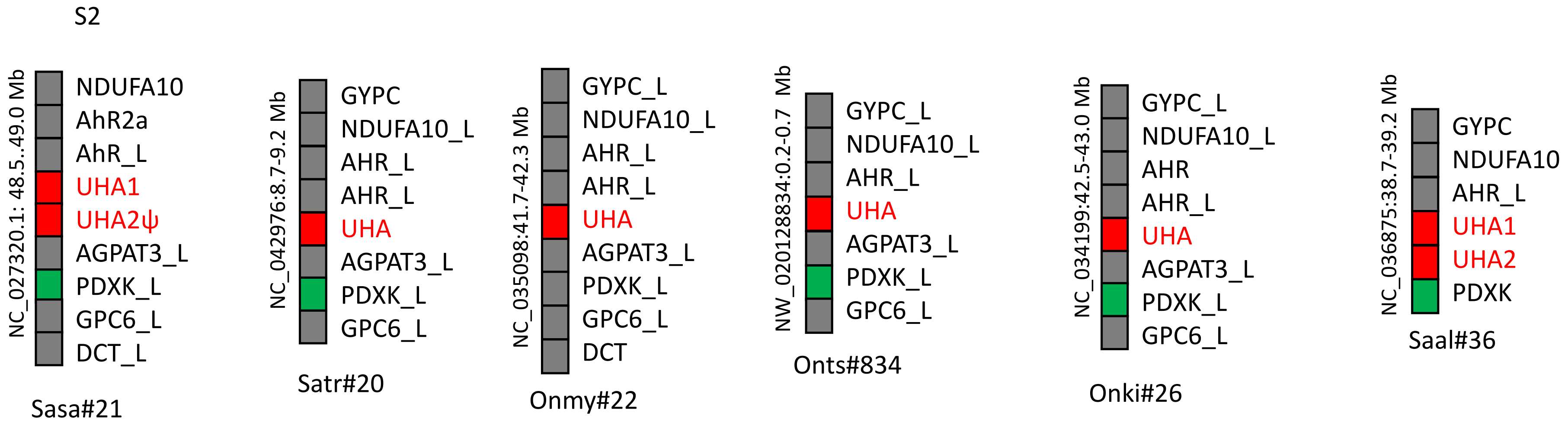

AF2.5. L lineage region 1

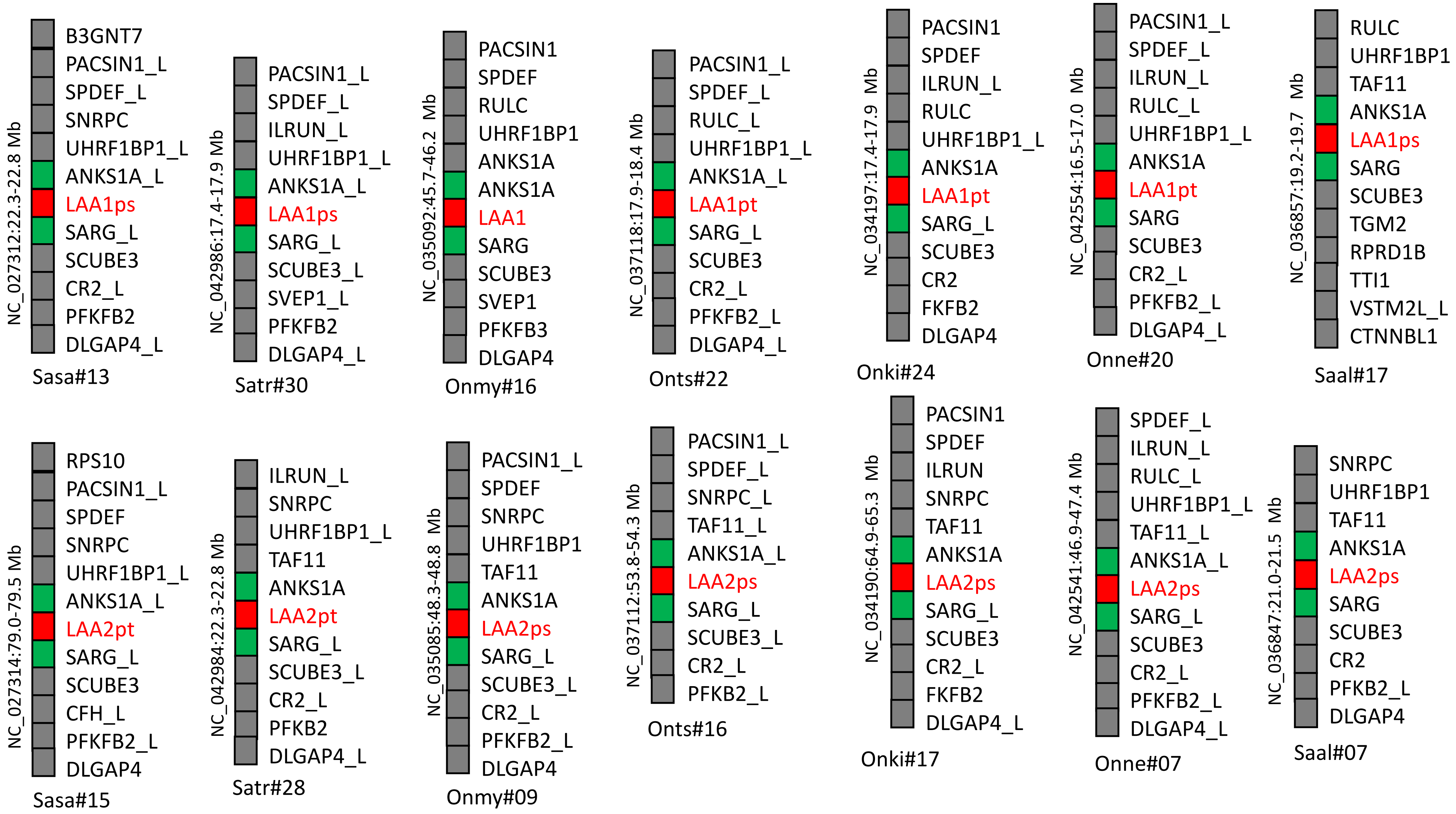

AF2.5. L lineage region 2

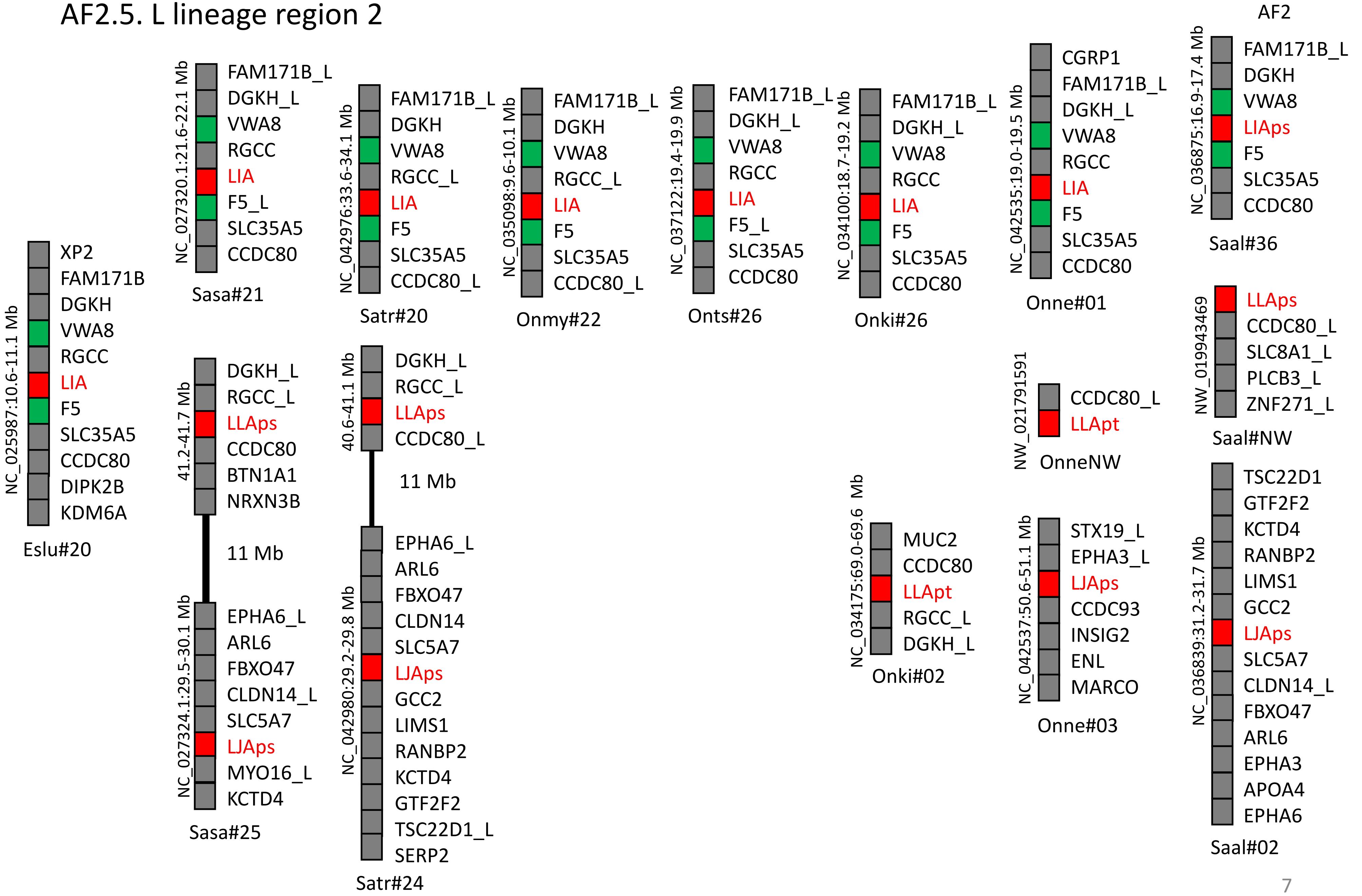

AF2.5. L lineage region 3

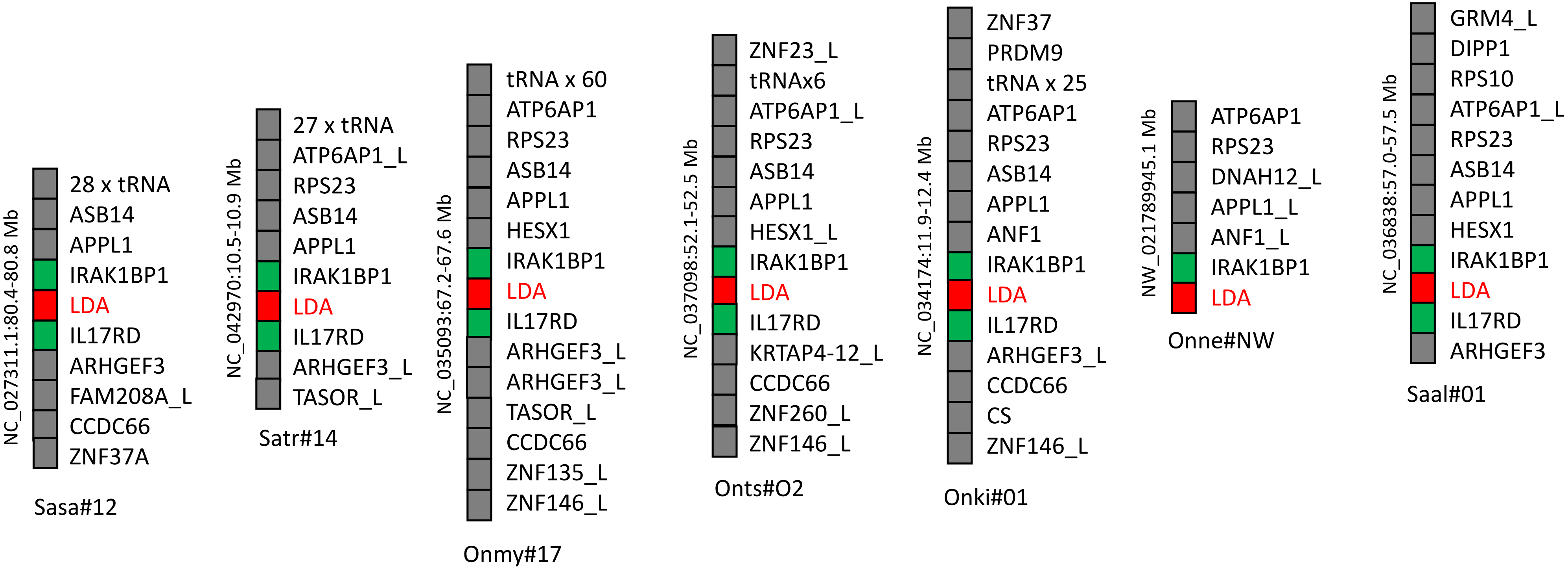

AF2.5. Unique L lineage regions

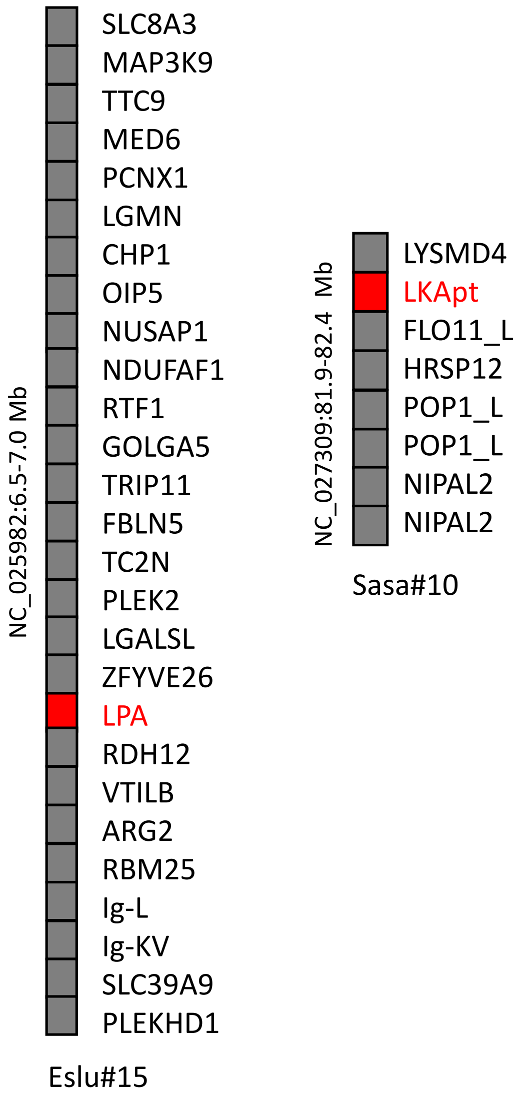

AF2.6. S lineage regions

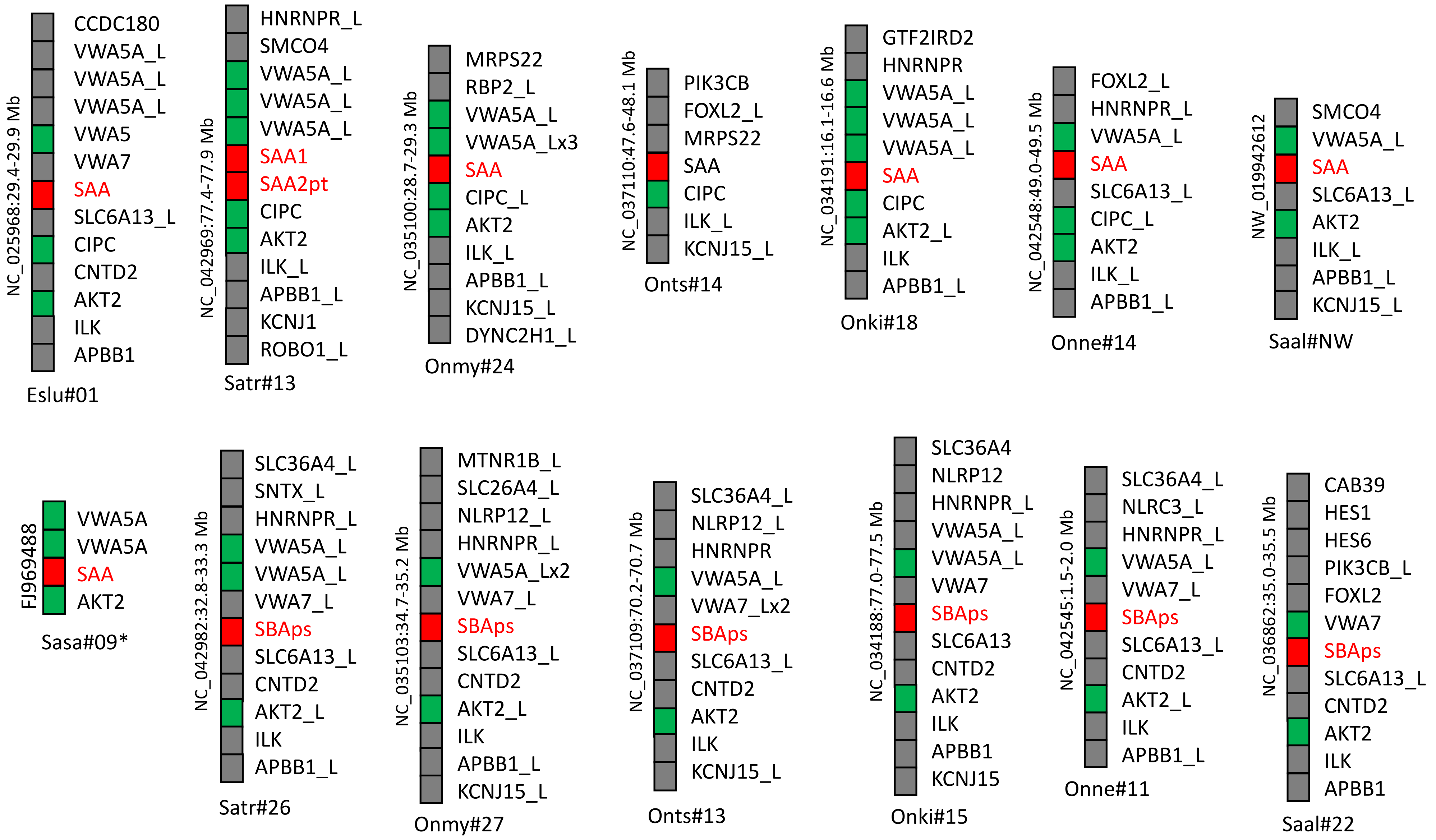

AF2.7. H lineage regions

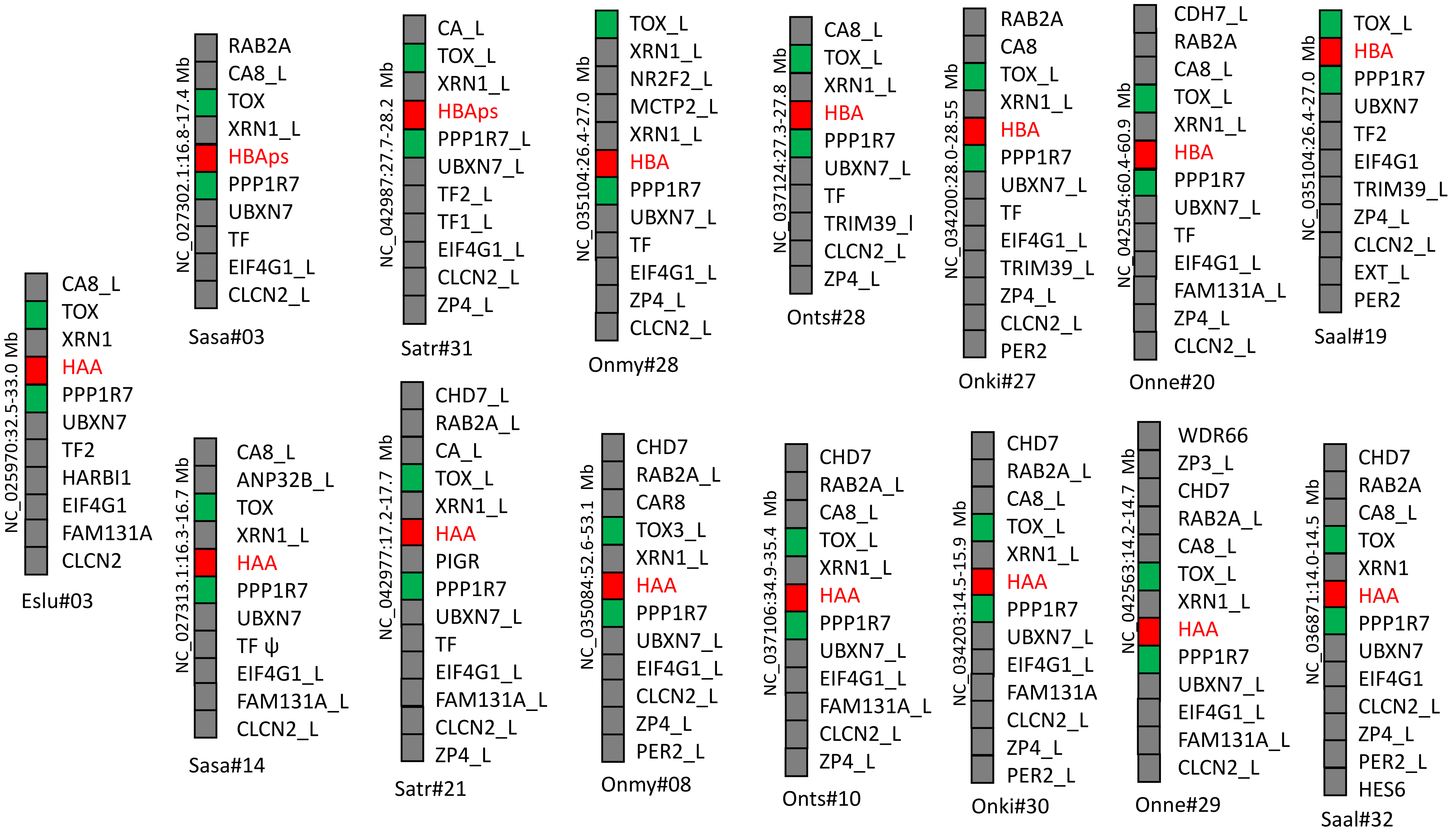

AF2.8. P lineage regions

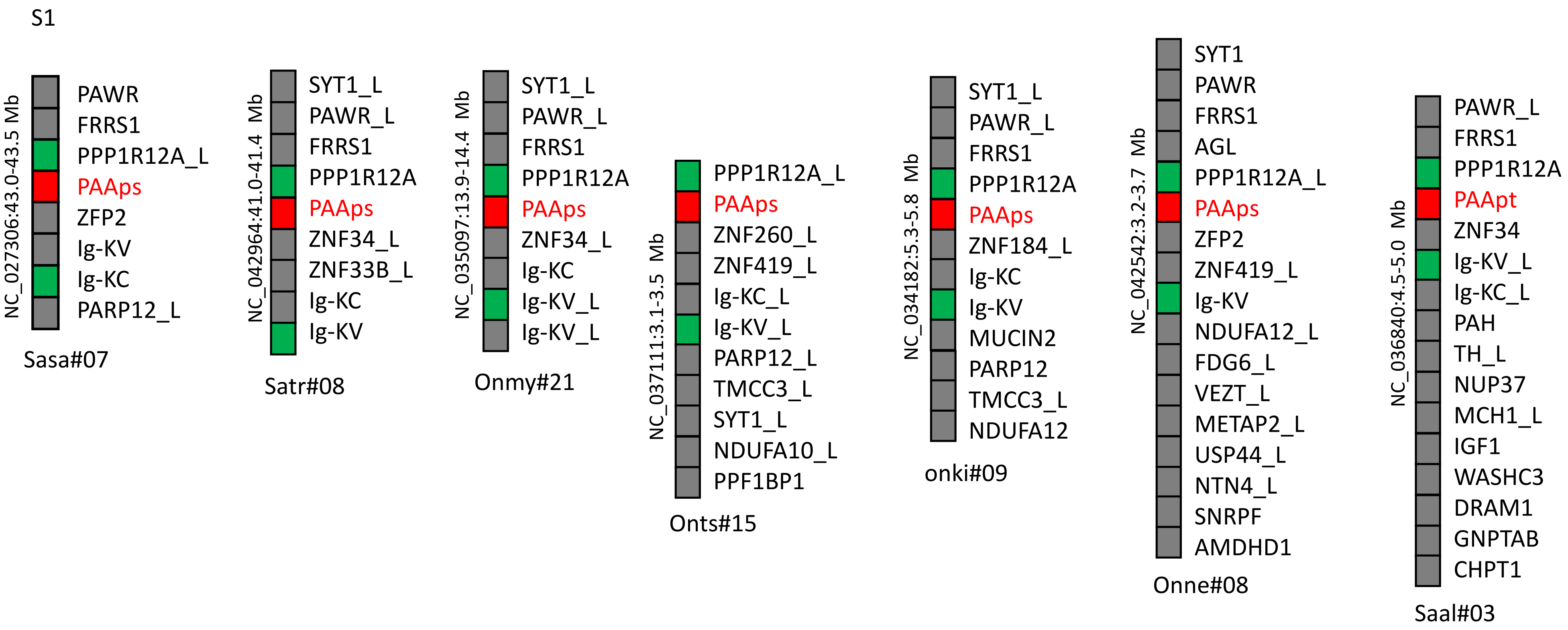

Supplement: Supplementary file 2 — Additional file 2: Compared MHCI regions. [file 12862_2020_1736_MOESM2_ESM.pdf]
