# Supplementary material for: MHC class I evolution; from Northern pike to salmonids
Source: BMC Ecol Evol. 2021 Jan 21;21:3. doi: 10.1186/s12862-020-01736-y (PMC7853315; doi:10.1186/s12862-020-01736-y)
Supplement: Supplementary file 3 — Additional file 3: Phylogeny of U lineage sequences. [file 12862_2020_1736_MOESM3_ESM.pdf]

Additional file 3 (AF3). Phylogeny of U lineage sequences

AF	Table of Content	Page
AF3.1	Deduced U lineage extracellular domain sequences	2
AF3.2	Deduced U lineage alpha 2 domain sequences	3
AF3.3	Deduced U lineage alpha 3 domain sequences	4

The evolutionary history of the phylogenetic trees presented in this supplementary file were inferred by using the Maximum Likelihood method based on the JTT matrix-based model (Jones et al.2002. Deduced amino acid sequences are used in all trees. The trees with the highest log likelihood are shown. The percentage of trees in which the associated taxa clustered together are shown next to the branches. Initial tree(s) for the heuristic search were obtained automatically by applying Neighbor-Join and BioNJ algorithms to a matrix of pairwise distances estimated using a JTT model, and then selecting the topology with superior log likelihood value. A discrete Gamma distribution was used to model evolutionary rate differences among sites. The trees are drawn to scale, with branch lengths measured in the number of substitutions per site. All positions with less than 95% site coverage were eliminated. That is, fewer than 5% alignment gaps, missing data, and ambiguous bases were allowed at any position. Evolutionary analyses were conducted in MEGA7 (Kumar et al.2016). See Additional file 4 for sequences and genomic location. Partial genes and pseudogenes were with a few exceptions omitted from the phylogenic analyses.

Jones D.T., Taylor W.R., and Thornton J.M. (1992). The rapid generation of mutation data matrices from protein sequences. Computer Applications in the Biosciences 8: 275-282.

Kumar S., Stecher G., and Tamura K. (2016). MEGA7: Molecular Evolutionary Genetics Analysis version 7.0 for bigger datasets.Molecular Biology and Evolution 33:1870-1874.

AF3.1 Phylogeny of U lineage Extracellular Domain sequences

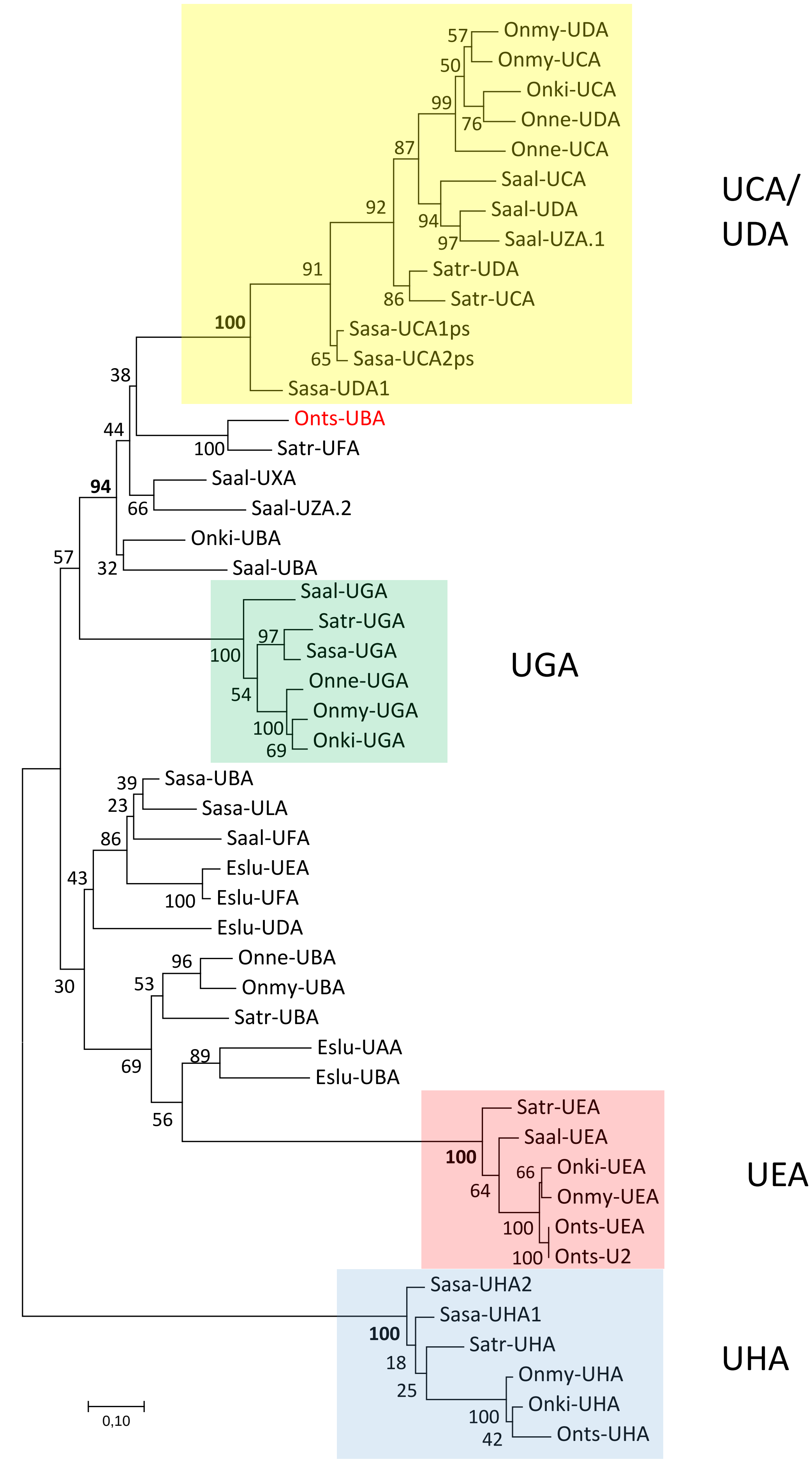

AF3.1. The tree with the highest log likelihood (-8334,57) is shown. A discrete Gamma distribution was used to model evolutionary rate differences among sites (5 categories (+G, parameter = 1,1067)). The analysis involved 48 amino acid sequences. There were a total of 255 positions in the final dataset

AF3.2 Phylogeny of U lineage alpha 2 domain sequences

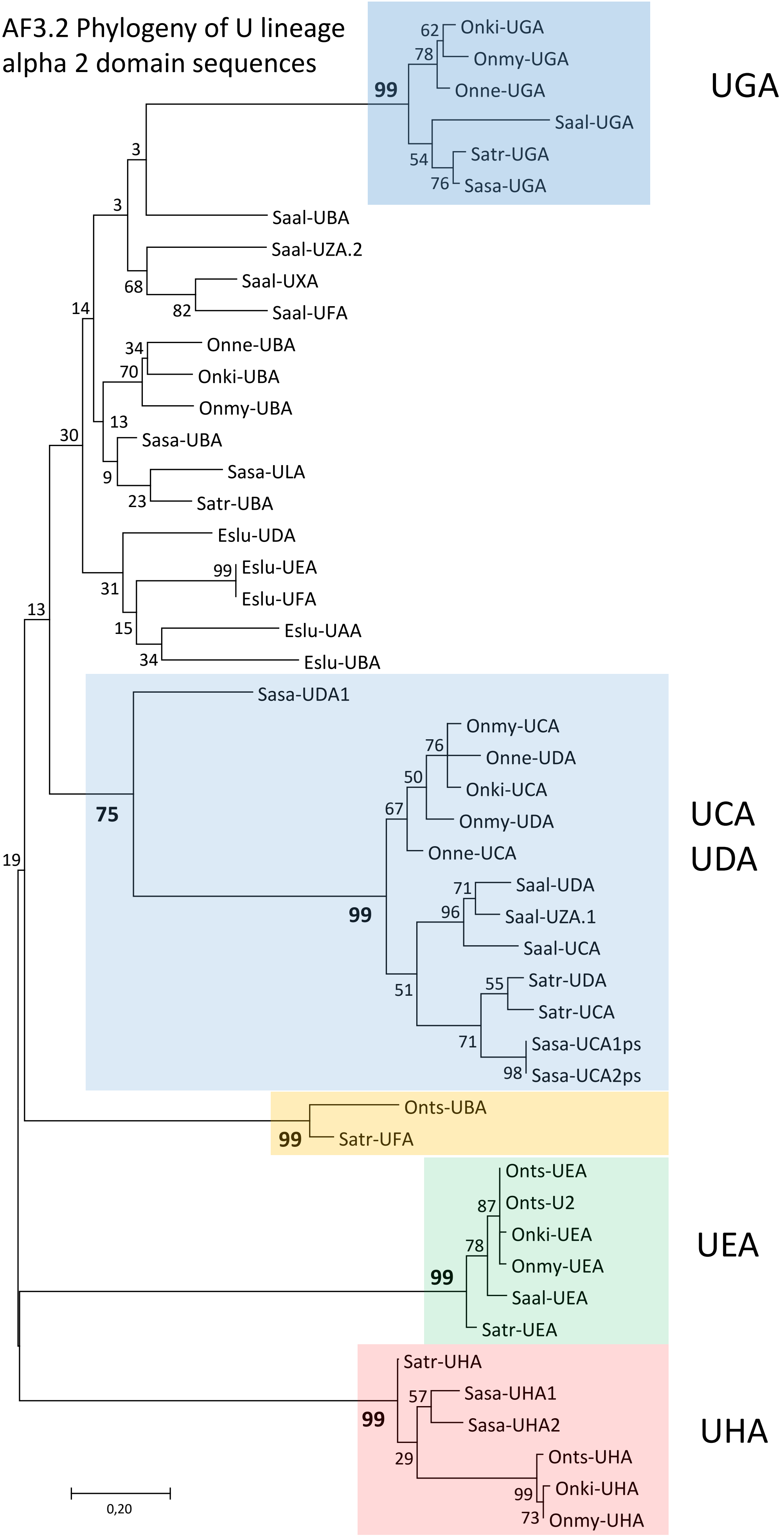

AF3.2. The tree with the highest log likelihood (-3135,50) is shown. A discrete Gamma distribution was used to model evolutionary rate differences among sites (5 categories (+G, parameter = 1,4071)). The analysis involved 48 amino acid sequences. There were a total of 91 positions in the final dataset.

AF3.3 Phylogeny of U lineage alpha 3 domain sequences

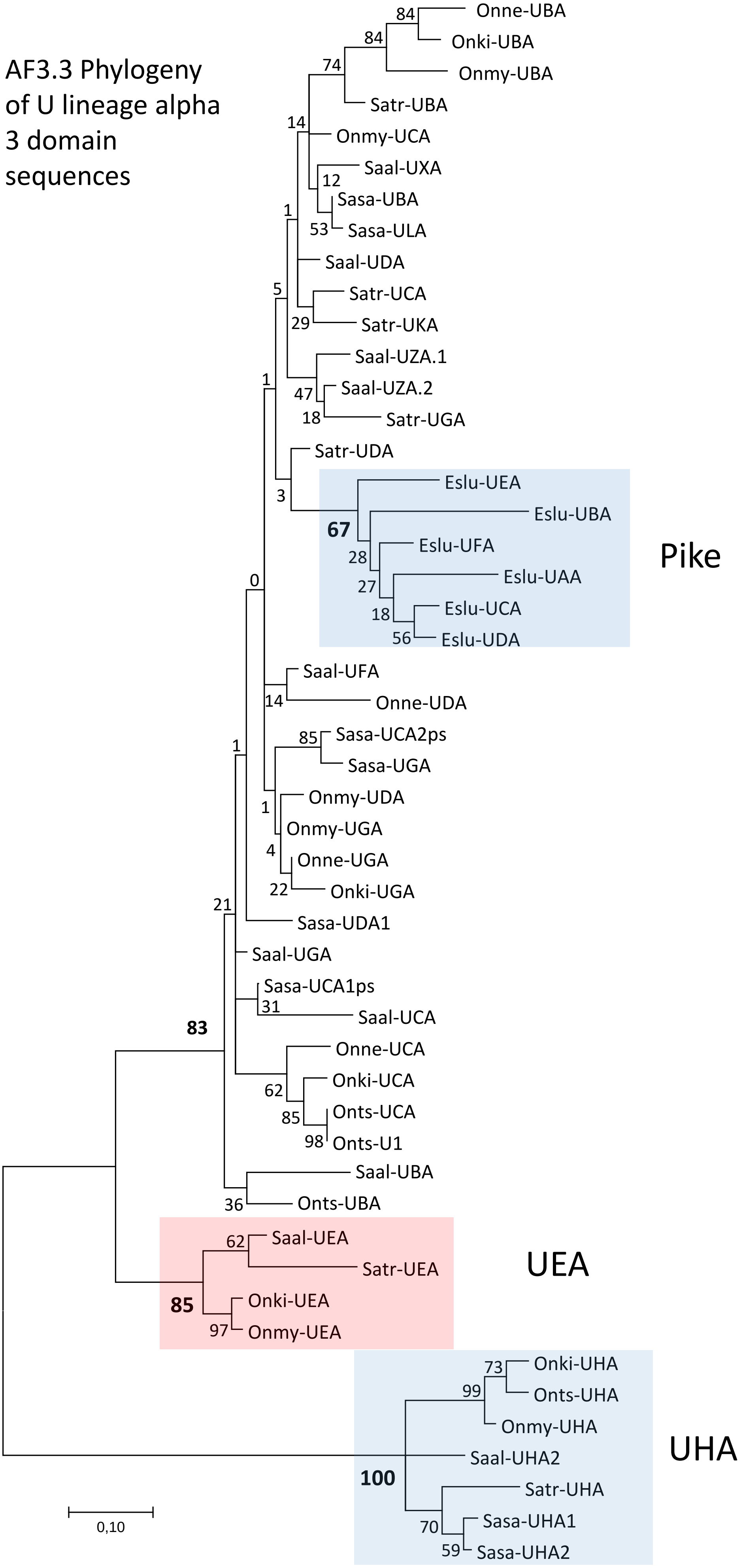

AF3.3. The tree with the highest log likelihood (-1871,26) is shown. A discrete Gamma distribution was used to model evolutionary rate differences among sites (5 categories (+G, parameter = 0,9744)). The analysis involved 50 amino acid sequences. There were a total of 87 positions in the final dataset.
