# Supplementary material for: MHC class I evolution; from Northern pike to salmonids
Source: BMC Ecol Evol. 2021 Jan 21;21:3. doi: 10.1186/s12862-020-01736-y (PMC7853315; doi:10.1186/s12862-020-01736-y)
Supplement: Supplementary file 4 — Additional file 4: Deduced MHCI amino acid sequences. [file 12862_2020_1736_MOESM4_ESM.pdf]

Additional file 4 (AF4). Deduced MHC class I amino acid sequences from selected salmonids

Deduced amino acid sequences from *Salvelinus alpinus/malma* (Saal), *Oncornhynchus nerka* (Onne), *Oncorhynchus kisutch* (Onki), *Oncorhynchus tshawytscha* (Onts), *Oncorhynchus mykiss* (Onmy), *Salmo trutta* (Satr), *Salmo salar* (Sasa) and *Esox lucius* (Eslu) originating from the NCBI genomes described in main text Material and Methods. When available, the GenBank protein accession number and chromosomal location is provided for each sequence. Pseudogenes defined as having internal stop codons are shown using a ps extension to gene name while partial gene names have been given a pt extension.

AF4.1 Deduced U lineage MHCI amino acid sequences

```
>Saal-UBA XP_023832378.1 chr.31 NC_036870:9.674.553-9.682.654
MNVNMKGFVLLVLGIGLLHTASAVTHSLNYFYTASSEIPNFPEFVALSMVDGVQTVYYDS
NSQRAVPKQDWMNKATETLPRYWETVTESFMGNQPNFKANIDIAKQRFNQSGGVHISQRM
YGCEWNDETGATGGYFQDGYDGEDFLALDLKTKTWIAPKPQAVITKYKLDSDTAQNEYLK
NYFTQECIEWLKKYLDYGKSTLMRTVRPSVSLLQKTPSSPVTCHATGFYPSGVMVFWQKE
RQDHHEDVEHGETLQNDDGTFQKSTHLTVTPEEWKKTQYQCVVQVTGIKEDIITVLTESE
IKTNWNATNFVPIIGGVVALLVIVAVVVGVVIWKKKSKKGFVPASTSDTDSENSGRAAQQ
I
>Saal-UCA XP_023830372.1 chr.30 NC_036869:18.153.603-18.167.426
MKVFILMFLGIGHLYEAFGTIHSLKHFYTASSEVSNFPKFVVVGMVDGVHMVHYDSNSQK
VVPEQDWMKQTDAEFWESERENFFDSQQSFKAEVHILKQRFNQSGGVHVLQYIYGCSLDN
ESEQKEGFGHLGYNGENFLEYDMKKSSWKSLKQQADFMQDKWNSDISRLNFWKFYFTQTC
IECLKKQVVNGKSILMRTVPPSVSLLQKTPSSPVTCHATGFYPSGVMVSWQKDGNDQHED
VEHGEILLNDDESFQKSTHLRVTPEEWKNNKYQCVVQITGIKEYFIKVLTESEIQTNWVD
PAPIIGGVVALLLVIVVVVVGVVIWKKNSNKGFLPASTDEDNSVRGFLKT
>Saal-UDA XP_023990448.1 chr.30 NC_036869:18.081.405-18.087.963
MKGFILMFLGIVHLHEAFGAIHCLTHFYTASSDIPNFPEFVVVGMVDGVQIVHYDSNSQK
VVPKQDWMKQTDAEFWESEREKFFNSQQSFKVEVHILMQRFNQSGGVHILQYIYGCSLDN
ESEQKEGFGQLRYNGENFLVYDMKTLAWKSLKQQADFMQDKWNHDHSRLVFWRIYFTQTC
IECLKKQVVNGKSILMRTVPPSVSLLQKTLSSPVTCHATGFYPSGVMVVWQKDGQDHHED
VEHGQXLPNDDGTFQKSTHLTVTPEEWKNNKYQCVVQVTGIKEDFIKVLTESEIQTSWGK
TNRGTNDPNTIGSAPIIGVAVALLVVVVVVVGVVIWKKKREKGFVPASSSDTDSENSWKG
AQKT
>Saal-UEA XP_023830369.1 chr.30 NC_036869:18.033.964-18.036.877
MISFCLLFLWIPITSAAPHSMKYLYTAVSGDTDFPEFTVVGLLDDHQFVYFNSNTKTLVL
TTEWMKKKAGKYYRKLYTEPLINQHEGFKNLITFAKKQFNQTQSKGVHTIHNLYGCEWND
ETELKDYFHHYGYDGEDFISLDMKTVRWITFVQQADTIKQKWDDNSKDLHYLKWYFTKEC
IDTLKKYLNFASSVLKRIVPPSVSLLQKTPSSPVTCHATGFYPSGVTVSWQKDGQEQHED
VLHGEILPNDDXTFQKSAHLTMDCDEWKNNNYTCVVEHKENIIAIRLDQSVIKTNSDKPP
ESGPIIIGVVVAVILLALITIAGFIMWNRKRTVSDDGFNPSMTQQNQIQLEVSLSLIKQE
RSRENRTFIEGGEAS
>Saal-UFA XP_023830370.1 chr.30 NC_036869:18.000.010-18.018.603
MKGFILVVLGVGLLHCASAATHSLRYVYTATSGIPAFPEFVTVGLVNGEPISYYDSVIRR
ETPRQDWMAKSEGPDYWERETLISIGAEQTFKVGIENIKPRFNQTGGVHTWVNMYGCEWD
EDTGVTKGFNQFGYDGEDFVAFDLKTKTWIAPTPQAVITKHKWERDIAYNEQRKYYLTQT
CIEWLKKYLDYGKSTLMRTVPPSVSLLQKTPSSPVTCHATGFYPSGVMVFWQKDGQDQHE
DVEHGETLPNDDGTFQKNTHLTVTPEEWKNNKYRCVVQVTGIKEDFIKVLTESEIQTNWG
KTNRGANDPNTIGLAPIIGVAVALLVVVVAVVGVVMWKKKSKTGFVPASTSDTDSENSGK
GAQKT
>Saal-UGA XP_023830918.1 chr.30 NC_036869:17.644.777-17.650.392
MKTRLISAMKTYFILLFCIHGALSVAHFLRYFYTASSGIPDFPEFVDMGMVNDQVISRYD
SITKRKVPKQRWMEKVFDQQYWDSTTENLREAEQVFKANIQIAQKRFNDTGGMHINQDMY
```

GCEWDDETGFTEGFHHVGYNGKDLLAFDLQRATWIAFVPQAVHSKMKWESDKSAIEMERH
YLTQTCIXWLKMYLNYGKSTLQRTXPPSVSLLQKTPSSPVTCHATGFYPSGVMVFWQKDG
QEQHEDVEHGEILPNDDGTFQKSTHLTVTPEEWKNNKYQCVVQVTGIKEDFIEVLTESEI
QTSWGKTNRGDPAPNIVPIIGGVVALLLVVVGVVIWKKKSKKGFVPASTSDTDSENSGKX
AQKI
>Saal-UXA XP_023996926.1 NW_019943742:11.208-50.680
MKDFILLVLGVGLLHTASAVTHSMKYFYTGSSEVSNFPEFVVVGMVDGVQMVHYDSNSQR
AVPKQDWMTRHTDTQYWERNTGIFMNSQQIFKVDINTLKQRFNQSGGVHTLMEMYGCEWD
EDTGVTEVFKQFGYDGEDFVAFDLKTKTWIAPTPQAVITKHKWDNDMSYNEYWKNYLTQT
CIEWLKKYLDYGKSTLKRTVPSSVSLLQKTPSSPVTCHATGFYPSGVMVSWQKEGQNHHE
DVEHGETLHNDDGTFQKSTHLTVTPEEWKNNKYQCVVQVAGIKEDFIKVLTESEIQTNWG
KTNRGANDPNTIGSIAMEPVIGVAVALLVIVVIVVVGVVIWRKMKSEKGVRREGCFVPTS
TSDTDSENSWKGFQKT
>Saal-UZA.1 XP_023997137.1 NW_019943825:6.883-38.250
MKGFILMFLGIVHLHEAFGAIHSLMHFYTASSDIPSFPEFMVVEMVDDVQMVHYDSNSQK
VVPKQDWMKQTDAGFWESEREKFFHSQQSFKVEVHILMQRFNQSGGVHILQYIYGCSLDN
ESEQKEGFGQLRYNGENFLEYDMNDLTWKSLKQQADFMQDKWNSDSSRLVFWKIYFTQTC
IECLKKQVVNGKSILRRTVPPSVSLLQKTPSSPVTCHATGFYPSGVKVVWIKDGQEHHED
VEYGETLQNDDGTFQKSTHLTVKPEEWKNNKYQCVVQVTGIKEDFIKVLTESEIQTNRGD
PAPNFVPIIGGVVALLLVVVVVVVVGVVIWKKRSKKGFIPASTSDSDSENSGKGAQKI
>Saal-UZA.2 XP_023997139.1 NW_019943825:66.739-90.643
MKGFVLLVLGIGLLHTASAVTHSLKYFYTASSGVTNFPEFVGVGMVDDVQMVHYDSNSQR
VVCKQDWMNKQPDTQYWEREKVNLLGSQQIFKVNINTLKQRFNQSGGVHVFQKMYGCEWD
DESGATEGFNQFGFDDFEDFVALDLKTETWTAPTPQAVITKHKWENLSANTKHWKNYHTQ
TCIEWLKKYLDYGKSTLKRTVPPSVSLLQKTPSSPVTCHATGFYPSRVKVVWIKDGQEHH
EDVENGETLPNDDGTFQKSTHLTVTPEEWKNNKYQCVVQVTGFKEDFIKVLTESEIQTSW
GKTNRDTNVPNTVGSAPVIGVAVALLVVVVAVVVGVVIWKKK
>Saal-UHA1 XP_023837329.1 chr.36 NC_036875:38.986.008-38.984.679
VYVTVLLLFPSINLYLCLPIFFLATHSLKYFYTALPQSTGLPEFSAVAYLDEEPIYSYDS
STKEVVAQQEWVKEAVDPDFWGRSTHILMETEKVFKDNMNTARARFNQTSAHTLQKMYSC
EWDEETGATEGREQYGYGGEDFLLFDLKNERWIAPVRQGLITKMKWDDNVPKLEAKIHYL
THTCIEWLKKYVFYRRSMLEKTSYPVTCHATGFYYQKRPISLCPEDLQKYDYTCTVHTLE
VMCVLYANRDSIYSNSRNRQG
>Saal-UHA2 chr.36 no predicted orf NC_036875:39.022.250-39.028.661
MYTVSNRIVHISTLLVTYLIPVTCAGEHSLKYFYTALPQPTGLPEFSAVAYLDEEPIYSY
DSSTKEVVARQEWVEGAVDPDFWRRNTTAVRGMRKLVLQRDVNSMDMRRDFLLFDLKNKR
WIAPVWQGLITKIKWDANVTVLPQVSLLQKNLSYPVTCHATGFYPNAIMIFWRRDGVVIH
DDVVHEETLPNGDGTYQKRTHLTVFPEDLQKHDYTCTVQHSGDDVVLSANRDSIRRNTQG
>Onne-UBA XP_029514050.1 chr.4 NC_042538:45.484.905-45.509.590
MNVKMNGFISLVLGIGLLHTASAATHSLKYFYTAVSGDIDYPEFTAVGLVDNGQFMYFDS
STKTAVPKTEWMKREGADYWDSQTQGLIGEHQTFKVNIQTLKDRFNQSKSTGVHVVQRMY
GCEWDDDSGATEGFDQYGYDGEDFIAFDLKTTKWIAPAPQAVITKLKWDSNTANNEHRKN
YYTQECIDWLKKYMDYGKSTLMRTVRPSVSLFQKTSSSPVTCHATGFYPSDVMVSWQKDG
QDHHEDVEYGETLPNDDGTFQKSIHLTMTPEDRKNNKYQCVVQVKGIKDDFIRVLPDQDA
TNVVPIIGGVVALLLVVVAVVVCVVIWKKRSKKGFVPTSTSDTDSDNSGRAAPQI
>Onne-UMApt chr.07 NC_042541:7.582.558-7.584.184
YSGTSGIPDLPEFVTLVLVNGNPFSYYDSNISRMTHKQEWIAKSEGPDYWERNTQLYIGN
ELLPKTNLVVSQQRFNQTGVHISQVMYGCEWDEETGITDVLFQYGHDREDFISFDLKRMT
WVTPKPQAVITKHHKYNIAVNEGYKHYLTHRCITCLKKYLQNGKSTPQRIVSILQKTPSSP
VVTCHATGFYPRGVMVMRMRGELDIHEDVKYGETLPNHDGIFQMRSRLTVDPKEWDNNNT
>Onne-UCA XP_029519320.1 chr.7 NC_042541:17.660.146-17.669.552
MKGFILMVLGIVHLHEAFGVTHSLTHFYTASSGITNFPEFMVVGMVDGVQMVHYDSNIQK
AVPEQGWMKQTDAEFWEKERDNLFDFQQSFKAEIATLKQHFNQSGGVHILQYMYGCSQDY
ETEQTEGFGQLGYNGEDFLEYDMKTLTWKSRKQQANFMQDEWNSDTSRLLFWKNYFTQTC
IECLKKQVVNGKSTLMRTVPPSVSLLQKTPSSPVTCHATGFYPSGVMVFWQKYGKYQHED
VENGEILVNDDGTFQKSAHLTVTPEEWKNNKYQCVVQVTGIKEDLIKVLTESEIQTNWKD
PAPNIILIIGGVVALLLVVVGVVIWKKKKKKGFVPASTNDTDSAYSGKGLLKT
>Onne-UDA XP_029519318.1 chr.7 NC_042541:17.635.043-17.646.967
MKGFMLMFMGIGHLYEAFAVTHSLEHFYTASSELHNFPEFMVVGMVDRVQMVHYDSNIQK
VVPEKDWMKQTDTEFWEKERENFFYSQQSFKAEIANLKQHFNQSGGVHILQYMYGCSRDD
ETEQTERFGQLGYNGENFLEYDMKTLTWKSRKQQAHFMQDEWNSDISRLDFWKNYFSQTC
IDCLKKQVDKGKSTLMKTVPPSVSLLQKTPSSPVTCHATGFYPSGVMVFWQKDGQDHHED
VENGETLPNDDGTFQKSTHLTVTPEKWNNQNYRCVVQVTGTKEVFIKVLTESEIQTNWED

PAPNIILIIGGVVALLLVVVGVVIWKKKSKKGFVPASTSDTDSDNSGKGVQKT
>Onne-UGA XP_029519296.1 chr.7 NC_042541:17.344.654-17.353.783
MKTRLIGAMKTYFIILFCIHGALSVKHSLRYFYTTSSEIPAFPEFVDMGMVNDQVISRYD
SITKRKVPKQSWMETVFDQQYWDSTTKDLSGAESVFKTNIQIAQKRFNQTGGMHISQDMY
GCTWDDETGFTEGFHHIGYDGQDLLAFDVKTATWIASVPQALHSKMKWEMDLSSIEIKKS
YLTQDCIKWLKKYLDYGKTTLQRTVPPSVSLLQKTPSSPVTCHATGFYPSGVMVSWQKDG
QEQHEDVENGETLPNDDGTFQKSTHLTVTPEEWKNNKYQCVVQVTGIKEDFIKVLTEFEI
QTNWGDPAPNIILIIGGVVALLLVVVVVVVVIWKKKSKKGFVPASSSDTDWDKSGQGFL
>Onki-UCA XP_031645085.1 chr.14 NC_034187:55.902.447-55.906.191 dual orf
MKAFMLMFMGIGHLHEAFAVTHSLEHFYTASSELHNFPEFMVVGMVDGVQMVHYDSNIQK
AVPEKDWMKQTDTEFWEKEKENFFYSQKSFKAEVANLKQHLNQSGGVHILQYMYGCSRDD
ETEQTEGFGQLGYNGENFLEYDMETLTWKSRKPQANFMQDEWNSDISRLGFWKNYFSQTC
IDCLKKQVDNGKSTLMRTVPPSVSLLQKTPSSPVTCHATGFYPSGVMVFWQKYGKYQHEG
VEHGEILFNDDETFQKSTHLTVMPEEWKNNKYQCVVQVTGIKKDFIKVLTESEIQTNWKD
PAPNIVLYIGGVVAFLLVVVVVVVGVIIWKKKSK
>Onki-UEA XP_031645085.1 chr.14 NC_034187.2:55.926.414-55.927.642 dual orf
MISFCLLFLWIPTMSADPHSLKYIYTAVSGDTDFPEFTVVGLLDDQQFVHFGSNTKTLVN
DAEWMNKTGKYYRELYNETLINQYEGFKNLITFAKKQFNQTQSKGVHTIQNLYGCEWNDE
TELQDYFHHYGYDGEDFISLDMKTVRWITSVQQADTIKQKWDDNSKDLHYLKWYFTKECI
DTLKEYVNFASSVLKKIVPPSVSLLQKTPSSLVTCHATGFYPSGVMVSWQKDGQEQHEDV
LHGEILPNGDGTFQKSAHLTIDSEKWENNNYTCVVEHKENIIAIRLNQSVIKTNSVKLLE
TVPIIIGGGVVAVILLVTFIITRFIMWNRKSTASDDGSNPSMTQQNQIQLEVSLSLIKQE
RSREKRTFIEGQ
>Onki-UGA XP_031645087.1 chr.14 NC_034187:56.227.506-56.231.443
MTAYILSTFSVTVRHSLRYFYTTSSEIPAFPEFVDMGMVNDQVISHYDSITKRKVPKQSW
MEPFFNQQYWDSTTENLRSAESVFKTNIQIAQKRFNQTGGIHISQDMYGCTWDDETGFTE
GFHHIGCDGQDLLAFDVKTATWIASVPQALHSKMKWEMDLSSIESKKSYLTQDCIEWLKK
YLDYGMTTLQRTVPPSVSLLQKTPSSPVTCHATGFYPSGVMVSWQKDGQEQHEDVENGET
LPNDDGTFQRSTHLKVMPEEWKNNKYQCVVQVTGIKKDFIKVLTEFEIQTNWGDPAPNII
PIIGGVVALLLVVVVVVVVGVVIWKMKSKKGFVPARTSDTDSENSGERSPDDLKNFCNI
RELKRTATEITNRNFPRL
>Onki-UMApt XP_031645116.1 chr.14 NC_034187:67.374.482-67.376.232
MCDLSDFESDMIVSASCLSLSGVHISQVMYGCEWDEETGITDVLFQYGHDREDFISFDLK
TMTWVTPKPQAVITKHKYDSNIAVNEGYKHYLTQRCITCLKKYLQNGKSTLQRTHVSPPV
SILQKTPPSPVVTCHATGFYPRGVMVMRMRGEQDIHEDVKYGETLPNHDGIFQIRSRLTV
DPKEWDNNNT
>Onki-UBA XP_020361135.1 chr.17 NC_034190.2:33.698.029-33.719.534
MKGIILLVLGIGLLHTASAVTHSLKYFYTASSEVPNFPEFVVVGMVDGVQMVHYDSNSQR
AVPKQDWVNKAADPQYWERNTGNFKGSQQTFKANIDIVKQRFNQSGGVHIVQMMYGCEWD
DETGVTEGFEQHGYDGEDFIAFDLKTTKWIAPMPQAVITKHKLDSNTANNEYKKNYYTQT
CIDWLKKYVDYGKSTLMRTVCPSVSLFQKTSSSLVTCHATGFYPSDVMVSWQKDGQDHHE
DVEYGETLPNDDGTFQKSSHLTVTPEDRKNSKYQCVVQVKGIKEDFIKVLPDLDAANVVP
IIVGVVALLLVVVAVVVGVVIWRKRSKKGFVPTSTSDTDSDNSGRAAPQI
>Onki-UHA XP_020316994.1 chr.26 NC_034199:42.842.475-42.852.669
MVGQILVLWLCLNLPTANSATHSLKYFYTALPQSTGLPEFSAVTYLDEEPIYFYDSSTKE
VVARQEWVKEAVDPDFWRRNTQILKETEKVFKNNMDTARDRFNQTSARTLQVMYSCESVE
ETGATEGHEQYGYGGEDFLLFDLKNERWIAPVRQGHITKIKWDANVPKLKAKIHYLTHTC
NEWLNKYVSNRRRMLQRTAPPQVTLFQKEPFYRVTCHATGFYPNAIMIFWRRDGVDVHGD
VVHEETLPNGDGTYQKRTHLTVSPEDLQKHDYKCTVKHSGNDVVLSANRDSIRSNSRNTQ
GVSIIITINMVLMLVILAFMVTVLVLKRTRDCKGKEPTTGKEKQCPISHLEKNVILLNNS
KPAEMEKSKLSHSADTLQCPTQDTTGSDRTSNRSRTISSSSSGGSDDSGDSTTPLVKKL
>Onts-UHA XP_024258185.1 NW_020128834:520.036-527.062
MRSYPFPEVFLHSAPTGLPEFSAVTYLDEEPIYFYDSSTKEVVARQEWVKEAVDPDFWRR
NTQILKETEKVLKNNIATARDRFNQTSARTLQVMYSCEWVEETGATEGHEQYGYGGEDFL
LFDLKNERWIAPVRQGHITKIKWDANVPKLEAKVHYLTHTCNEWLNKYVSNRRRMLQRTA
PPQVTLFQKEPSNPVTCHATGFYPNAIMIFWRRDGVDVHGDVVHEETLPNGDGTYQKRTH
LTVSPEDLQKHDYKCTVKHSGNEVVLSANRDSIRSNSRNTQGVSIIITINMVLMLVILAF
MVTVLVLKRRRDCKGKEPTTGKEKQCPISHLEKNVLLLNNSKPAEMEKSKLSHSADTLQC
PTQDTTGSDRIRTLSSSSSGGSDDSXDSTTPLVKKL
>Onts-UCApt XP_024251646.1 chr.31 NC_037127:27.243.272.27.248.291
MVHYDSNIQKAVPEKDWMKQTDTEFWEKERENFFYSQQSFKAEVANLKQHLNQSGVHILQ
YMYGCSRDDETEQTEGFGQLGYNGENFLEYDMKTLTWKSRKPQANFMQDEWNSDISRLGF
WKNYFSQTCIDCLKKQVDNGKSTLMRTVPPSVSLLQKTPSSPVTCHATGFYPSGVMVFWQ

KYGKYQHEDVEHGEILFNDDETFQKSTHLTVTPEGWKNNNKYQCVVQVTGIKEVFIKVLT
ESEIQTNWKDPSPNIVLHIGVVVALLLVVVAVGVIIWKSKKGFVPASTS
>Onts-UEA XP_024251644.1 chr.31 NC_037127:27.226.332-27.228.596
MISFCLLFLWIPITSADPHSLKYLYTAVSGDTDFPEFMVVGLLDDQQFVHFGSNTKTLVN
DAEWMNKTEKYYRELYTEPLINQYEGFKNLITFAKKQFNQTQSKGVHTIQNLYGCEWNDE
TELQDYFHHYGYDGEDFISLDMKTVRWITSVQQADTIKQKWDDNSKDLHYLKWYFTKECI
DTLKKYVNFASSVLKKIVPPSVSLLQKTPSSPVTCHATGFYPSGVMVSWQKDGQEQHEDV
LHGEILPNGDGTFQKSAHLTIDSEKWENNNYTCVCLYVCMYVCLSVSLSLSLSQERSREK
RTFIEGQ
>Onts-UMAps no gene prediction chr.31 NC_037127:16.450.518-16.449.323
YSGTSGIPDLPEFVTLVLVNGNLFSYYDSNISRMTH*QEWIAKSEGPDYWERNTQLYIGN
ELLTKTNLVVSQQRFNQTGVHISQVMYGCEWDEENGITDVLFQYGHDRENFISFDLKTMT
WVTPKPQAVITKHKYDSNIAVNEGYKHYLTQRCITCIKKYLQNGKSTLQRIVSILQKTPP
SPVVTCHATGFYPRMVMRMRGEQDSHEDVKY*ETLPNHDGIFQIRSRLTVDPKEWDNNNT
>Onts-U1pt XP_024256602.1 NW_020128813:6.051.676-6.056.695
MVHYDSNIQKAVPEKDWMKQTDTEFWEKERENFFYSQQSFKAEVANLKQHLNQSGVHILQ
YMYGCSRDDETEQTEGFGQLGYNGENFLEYDMKTLTWKSRKPQANFMQDEWNSDISRLGF
WKNYFSQTCIDCLKKQVDNGKSTLMRTVPPSVSLLQKTPSSPVTCHATGFYPSGVMVFWQ
KYGKYQHEDVEHGEILFNDDETFQKSTHLTVTPEGWKNNNKYQCVVQVTGIKEVFIKVLT
ESEIQTNWKDPSPNIVLHIGVVVALLLVVVAVGVIIWKSKKGFVPASTS
>Onts-U2 (UEA) XP_024256601.1 NW_020128813:6.071.372-6.073.634
MISFCLLFLWIPITSADPHSLKYLYTAVSGDTDFPEFMVVGLLDDQQFVHFGSNTKTLVN
DAEWMNKTEKYYRELYTEPLINQYEGFKNLITFAKKQFNQTQSKGVHTIQNLYGCEWNDE
TELQDYFHHYGYDGEDFISLDMKTVRWITSVQQADTIKQKWDDNSKDLHYLKWYFTKECI
DTLKKYVNFASSVLKKIVPPSVSLLQKTPSSPVTCHATGFYPSGVMVSWQKDGQEQHEDV
LHGEILPNGDGTFQKSAHLTIDSEKWENNNYTCVCLYVCMYVCLSVSLSLSLSQERSREK
RTFIEGQ
>Onts-UBA XP_024297406.1 chr.13 NC_037109:26.068.285-26.090.507
MKGIILLVLGIGLLHTASAVTHSLKYFFTASSEVPNFPEFVVVGMVDGVQMVHYDSNSQR
AVPKQDWVNKAADPQYWERNTGNCKGNQQTFKANIDIAKQRFNQSGGVHTFQWMYGCELG
DDGITRGDCQYGYDGADFLSLDKSTLTWTAANQKAVITKLKLDATGAVANNYKNYLENTC
IEWLKKYVNFGKDTLERKVRPSVSLLQKTPSSPVTCHATGFYPSGVMVFWQKDGQEQHGD
VEHGEILQNDDGTFQKSTHLTVTPEEWKNNKYQCVVQLAGIEDDITKVLIESEIQTNFGK
TNRGSNDPNTIGLIIGGVIALLVIIAVVGVVIWKKKNKKGFVPASTSDTDSENSGKGIQK
I
>Onmy-UBA XP_021427673.1 chr.18 NC_035094:33.174.619-33.200.831
MNVKMNGFISLVLGIGLLHTASAATHSLKYFYTAVSGDIDFPEFTAVGLVDKGQFMYFDS
STKTAVPKTEWMKREGADYWDRQTQGLIGAHQTFKVNIQTLKDRFNQSKSTGVHVFQWMY
GCEWDDEAGVTEGFDQYGYDGEDFIAFDLKTTKWIAPTPQAVITKHKWDSNTARNEQNKN
YYTQICIEWLKKYVDYGKSTLMRTVPPSVSLLQKAPSSPVTCHATGFYPRDVMVSWQKDG
QDHHEDVEYGEILQNDDGTFQKSSHLTVTPEDRKNSKYQCVVQVKGIKEDFIGVLPDQDA
ANVVPIIVGVVALLLVVVAVVVGVVIWRKRSKKGFVPASTSDTDSDNSGRAVPQI
>Onmy-UDA XP_021416252.1 chr.14 NC_035090:28.622.921-28.630.059
MKGFILMVLGIVHLHEAFGVTHSLTHFYTASSDIPNFPEFVFVGMVDGVQMVHYDSNIQK
VVPEQDWMKQTDAEFCEKERENLFYSQQSFKAEVATLKQHFNQSGGVHILQYMYGCSRDD
ETEQTEGFGQLGYNGENFLEYDMKTLTWKSLKQQPNFMQDEWNSDTSRLLFWKNYFTQTC
IDCLKKQVDNRKSTLRRTVPPSVSLLQKTPSSPVTCHATGFYPSGVMVFWQKDGQEQHED
VENGETLPNDDGTFQKSTHLTVTPKEWKNNKYQCVVQVTGIKEVFIKVLTESEIQTNWKD
PAPNIVLHIGGVVALLLVVVVVVGVVIWKKKKKKGFVPASTNDTDTSVLSALLKQLKTVT
K
>Onmy-UCA XP_021416249.1 chr.14 NC_035090:28.598.436-28.609.464
MKGLMLMFMGIGHLYEAFGVTHSLKHFYTASSDIPNFPEFVFVGMVDDVQMVHYDSNSQK
AVPKQDWMKQTDAEFWEKERENFFYSQQSFKAEVATLKQHFNQSGGVHILQYMYGCSRDD
ETEQTEGFGQLGYNGENFLEYDMKTLTWKSRKQQANFMQDEWNRDISRLGFWKIYFSQTC
IDCLKKQVDNGKSTLMRTVPPSVSLLQKTPSSPVTCHATGFYPSGVMVSWQKDGQDHHED
VENGETLPNDDGTFQKSTHLTVTPKEWKNNKYQCVVQVTGIKEDFIKVLTESEIQTNWKD
PAPNIVPIIGGMVALLLVVVVVVGVVIWKKKSKKGFVPASTSDTDSDNSGKGLLKI
>Onmy-UEA XP_021416247.1 chr.14 NC_035090:28.579.652-28.583.175
MISFCLLFLWIPSTSADPHSLKYLYTAVSGDTDFPEFTVVGLLDDQQFVHFGSNTKTLVN
DAEWMNKTGKYYNDLYNEPLINQYEGFKNLITFAKKQFNQTQSKGVHTIQNLYGCEWNDE
TELQDYFHHYGYDGEDFISLDMKTVRWITSVQQADTIKQKWDDNSKDLHYLKWYFTKECI
DTLKKYVDFASSVLKKIVPPSVSLLQKTPSSPVTCHATGFYPSGVMVSWQKDGQEQHEDV
LHGEILLNGDGTFQKSAHLTIDSEKWENNNYTCVVEHKENIIAIRLNQSVIKTNSVKPSE

YVPIIIGVGVVAVILLVLVPIITFIMWNRKSTASDDGSNPSMTQQNQIQLEVSLSLIKQE
EAEKRGPLLKDSEAS
>Onmy-UGA XP_021416226.1 chr.14 NC_035090:28.270.887-28.279.730
MKTRLIDAMKTNFIILFCIHGALSVRHSLRYFYTTSSEIPAFPEFVDMGMVNDQVISHYD
SITKRKVPKQSWMETFFDQQYWDSTTENLKSAESIFKTNIQIAQKRFNQTGGIHISQDMY
GCTWDDETGFTEGFHHIGYDGQDLLTFDVKTATWIALVPQALHSKMKWEMDLSGIKSKKS
YLTQDCIEWLKKYLDYGKTTLQRTVPPSVSLLQKTPSSPVTCHATGFYPSGVMVFWQKDG
QEQHEDVENGETLPNDDGTFQKSTHLTVTPEEWKNNKYQCVVQVTGIKEDFIKVLTEFEI
QTNWGDPAPNCIPIIGGVVALLLVVVVVGVVIWKKKSKKGFVPASSSDTDSDNSGKGLLK
I
>Onmy-UHA XP_021435429.1 chr.22 NC_035098:41.948.847-41.957.334
MYTVSNWIVHISTLLVTYLIPMTCAATHSLKYFYTVLPQSTGLPEFSAVTYLDEEPIYFY
DSSTKEVVARQEWVKEAVDPDFWRRNTQILKETEKVLKNNMATARDRFNQTSARTLQVMY
SCEWVEETGATEGHEQYGYGGEDFLLFDLKNERWIAPVRQGHITKIKWDANVPKLKAKIH
YLTHTCNEWLNKYVSNRRRMLQRTVPPQVTLFQKEPSYPVTCHATGFYPNAIMIFWRRDG
VEVHGVDVVHEETLPNGDGTYQKRTHLAVSPEDLQKHDYKCTVKHSGNDVVLSANRDSIR
GNSRNTQGVSIIIITINMVLISVILAFSVIVLVLKRTMYCKGKEPTTGKEKQCPISRLEK
NVLLLNNSKPAEMEKSKLSHSADTLQCPTQDTTGSDRSRTISSGSSVGSDDSGDSTTPFV
KKL
>Onmy-UMApt No prediction chr.14 NC_035090:17.785.888-17.784.737
YSGTSGIPDLPEFVTLVLVNGNPFSYNDSNISRMTHKQEWIAKSEGPDYLERNIQLYIGN
ELLTKTNLVVSQQLFNQTGVHIYQVMYGCEWDEETGITDVLFQYGHDREDFISFDLKTMT
WVTPKPQAVITKHKYDSNIAVNEGYKHYLTQRCITCLKKYLQNGKSTLQRIVSILQKTPS
SPVVTCHATGFYPRGVMVMRMRGEKDIHEDVKYGETLPNHDGIFQMR
>Satr-UBA XP_029587594.1 chr.34 NC_042990:10.539.672-10.557.140
MKGFILLVLGISLLHTASAAIHTLKNFYTAASGDISFPEFIIVGLVDNTPYLYFDSNTKT
AVPKTEWMKKSVGADYWDSMTQIGIGAHQTFKANIQVAKDRFNESKSTGVHVVQNMYGCE
WDDEAGVTEGFDQYGYDGEDFLAFDLKTKTWIAPKPQAVITKLKWDSNMAWKEQKKNYLT
QECIEWLKKYVDYGKSTLMRTVPPSVSLLQKTPSSPVTCHATGFYPSDVMVSWQKDGQDH
HEDVEYGETLPNDDGTFQKSIRLTVTPEEWKNSKYQCVVQVKGLKEDFIKVLTDSDATNI
VPIIGGVVALLLLIVAAVVGVVIWKKKSKKGFVPTSTSDTDSDNSGRAAQMT
>Satr-UCA XP_029591425.1 chr.36 NC_042992:32.893.949-32.904.220
MKGVILMFLGIVHLREAFSVTHSLKYFSTASSNIPNFPEFMVVGMVDGVQMVLYDSNSQK
VVPKQDWINKQTDAEFWESEREKFFDFQQSFKAEVDILKQRFNQSGGVHVLQYIYGCSRN
DETEQRDGFGKLGYNGEDFLVYDMNTLTWNALKQQANFMRDKWNNDNGRLVFWKTYFSQT
CIECLKKQVVNGKRPLMRTVPPSVSLLQKTPSSPVTCHATGFYPSGVMVVWKKDGQDHHE
DVKHGETLPNDDGTFQKSSHLKVTPEEWKNNKYQCVVQVTGIKEDFIKVLTESEIQTNWD
DPAPNIVPIIGVVVALLLLVVAVVVGVVIWKKKKSKKGFVPASTNDTDSAYSGKDLLKT
>Satr-UDA XP_029591428.1 chr.36 NC_042992.1:32.865.446-32.880.246
MKSFILIFLGIVEVHETFGVTHSLKYFYTGSSEVPNFPEFMVVGMVDGVQMVLYDSNSQK
VVPKQDWMNKQTDAAFWESEREKFFDSQQSFKAEVDILKQRFNQSGGVHVLQYIYGCSRN
DETEQRDGFGQLGYNGEDFLEYDMNTLTWNALKQQANFMRDKWNNDISRLGFWKIYFSQT
CIECLKKQVVNGKRPLMRTVPPSVSLLQKTPSSPVTCHATGFYPSGVMVVWQKDGQEQHE
DVKHGETLQNDDGTFQKSTHLTVMPEEWKNNKYQCVVQVTGIKEDFIKVLTESEIKTNWD
DPAPNIVLIIGGVVAILLQVVAVVGVVIWKKKSKKGFVPASTSDTDSENSGKGLLKT
>Satr-UEA XP_029591421.1 chr.36 NC_042992:32.837.610-32.841.400
MISFCILFLWIPITSAAPHSMKYLYTAVSGDTGFPEFTVVGLLDDHQFVYFDSNTKTLVP
TTEWMKEKAGEYYWDLYTEPLINQYEGFKNLIMFAKKQFNQTQSKGVHTIQNLYGCEWND
GTELKDYFHHYGYDGEDFISLDMKTMRWITSVQQADTIKQKWDNNSKDLHYLKWYFTKEC
IDTLKKYVNFASSILKRTVPPSVSLLQKTPSSPVTCHATGFYPRGVTVFWQKDGQDQHED
VVNGEILPNEDGTFQKRAHFTMDCEEWENNNYTCVVEHKENIITIRLDQSVIKTNSVKPP
ESGPIIIGVVVAVILLALITIAGFIMWNRKRTASDDGSNPSMTQQNQIQLEVSLSLIKQE
RSREKWTFIEGQ
>Satr-UFA XP_029591426.1 chr.36 NC_042992:32.791.364-32.726.587
MKDIILLVLGIGLLHTASAVTHSMKYFYTASSEVSNFPEFVVVGMVDGVQMVHYDSNSQR
TVPKQDWVNKAADPQYWERNTGYFMNSQQIFKVDINTLKQRFNQSGGVHALQRMYGCEMD
DDGVTRGYFQYGYDGADFLSLDKSTRTWTAANQKAVITKLKWDATGDNANIFKNYLENTC
IEWLKKYVNYGKDTLERKVPPSVSLLQKTPSSPVTCHATGFYPSGVMVVWQKDGQDHHED
VENGEILPNNDGTFQKSAHLKVTPEEWKNNKYQCVVQVTGIKEDFIKVLTQSEIHPNWGA
NDPNTVGSAPIIGGVVALLLLVVVVVVGVVMWRKKKSEKGFVPASTSDTDSENSGKGAQK
I
>Satr-UGA XP_029591397.1 chr.36 NC_042992.1:32.292.855-32.301.696
MKTRLISAMKIYFVLLNCIHVALSGTHSLRYLYTSSSGISDFPEFVDMGMVNDQIISHYD

```
SITKRKVPKQSWMKNVFDQQYWDGTTEDLRGAEKVFKNNIQTAQKRFNQTGGMHISQDMY
GCSWDDETGFTEGFHHIGYDGQDLLVFDLKRVTWIASVPQALHSKMKWEGDPSSIESEKR
YLTQDCIVWLKKYLEYGKTTLQRTVPPSVSLLQKTPSSPVTCHATGFYPRGVKVVWMKDG
QDHHEDVENGEILPNDDGTFQKSTHLTVMPEEWKNNKYQCVVQVTGITEDFIKVLTESEI
QTNWGVNTIGSAPIIGVVVALLLLVVVVVVVVGVVMWRKKSKKGFVPASTSDTDSENSGK
GAQKI
>Satr-UHA XP_029558050.1 chr.20 NC_042976.1:8.942.111-8.953.982
MYTVSNRIVHISTLLVTYLIPMTCAANHSLNYFYTALPQSTGLPEFTAVAYLDEELMYFY
DSSTKEVVARQEWVKGAVDPEFWRRNTHILKENEQVFKDNMDTARDRFNQTSALVLQKMY
GCDWDEATGATDGHEQYGYGGEDFLLFDLKNERWIAPGRQGLITKMKWDANVIKLEAKIH
YLTRTCIEWLKKYVSNRRRKLERTVPPQVTLLQKEHSYPLTCHATGFYPNAIRILWGRDE
VEIYDDVVHEETLPNGDGTYQKRIHLTVSPEDLQQHDYTCTVQHVSGDDVVLSANRDSIR
SNSRNTQVHYITIVLVLVPLAFSVIEPTAGKEKQCPTSLPEKSVHLLINGSDRTSNRSGT
ISSGFNCLLTLTTLQKGWKTSF
>Satr-UMAps chr.36 NC_042992:20.989.044-20.988.357
SGVHISQVM*YGTEWDEETGITDVLFQYGHDREDFISFDLKTMTLVAPKHYLTQRCITFL
KKYLQNGKSTLQRIVSILQKTPSSPVVTCHATGFYPRGVMVMWTRGEQDIYDDVKYGETL
PNHVGTFQMRSRLTVDPKEWDNNNT
>Sasa-UBA*0301 XP_014032819.1 chr.27 NC_027326:10.123.703-10.146.903
MKCFILLLLGIALHSSSAATHSLRYVYTATSGIPDFPEFVTVGLVNGEPISYYDSIIRRE
TPRQDWMAKTEGSDYWESQTQVSIGSEQTFKANIDVAKQRFNQTGGVHVNQKMYGCEWDD
ETGVTEGFDQDGYDGEDFLAFDLKTLTWIAPTPQAVITKLKWDSNTAQNEYRKNYLTQTC
IEWLKKYLDYGKSTLMRTVPPSVSLLQKTPSSPVTCHATGFYPSGVMVSWQKDGQDHHED
VEHGETLQNDDGTFQKSSHLTVTPEEWKNNKYQCVVQVTGLQEDFIKVLTESEIKTNWND
PNIVLIIGVVVALLLVVVAVVVGVVIWKKKSKKGFVPASTSDTDSDNSGRAAQMT
>Sasa-UDA1 XP_013996967.1 chr.14 NC_027313:50.985.016-50.996.125
MKGFILMFMGTCHLFEAFGVTHSLKYFYTASSGVPNFPEFMVVGMVDGVQIDHYDSNSQR
MVPKQDWMNKQTEAEYWERETGIAFDSQQVFKDDVNILKQRFNQSGGVHIVQKTYGCEWD
DETGQTGGFTQHGYDGEDFLGYDMKTFTWIAPKQQAEITRLKWNHDQAGLAFWHNYLTQT
CIEWLKKYVDYGKSTLRTVPPSVSLLQKTPSSPVTCHATGFYPSGVMVFWQKDGQEQHED
VEHGETLPNDDGTFQKSAHLTVTSEEWKINQYQCVVQVTGIKEDFIKVLTESEIQTNWDP
APIIGGVVALLLVVVVVVGVVIWKKKSKKGE
>Sasa-UCA2ps chr.14 NC_027313:51.036.934-51.045.017 Pseudogene
MKGFILMFMGTCHLFEAFGVTHSLKYFYTASSGVPNFPEFMVVGMVDGVQIDHYDSNSQR
MVPKQDWMNKQTEAEYWERETGIAFDSQQVFKADVDILKQRFNQSGGVHVLQYIYGCSWD
DETEQRD*GFGQLGYNGEDFLVYDMNTLTWKALKQQADVMRDKWNRDISRLVFWKTYFSQ
TCIECLKKQVVNGKSTLRTVPPSVSLLQKTPSSPVTCHATGFYPSGVMVFWQKDGQEQHE
DVENGETLHNDDGTFQKRTHLKVTSEEWKNNKYQCVVQVTGIKEDFIKVLTESEIQTNWD
PAPIIVPIIGGVVALLLVVVVVGVVIWKKKSKKGE
>Sasa-UCA1ps chr.14 NC_027313:59.088.400-59.091.612 Pseudogene
VTHSLKYFYTASSGVPNFPEFMVVGMVDGVQIDHYDSNSQRMVPKQDWMNKQTEAEYWER
ETGIAFDSQQVFKDDVNILKQRFNQSGGVHVLQYIYGCSWDDETEQRD*GFGQLGYNGED
FLVYDMNTLTWKALKQQADVMRDKWNRDISRLVFWKTYFSQTCIECLKKQVVNGKSTLRT
VPPSVSLLQKTPSSPVTCHATGFYPSGVMVFWQKDGQEQHEDVEHGEILHNDDGTFQKST
HLRVTPEEWKNNKYQCVVQVTGIKEDFIKVLTESEIQTNWDPAPIIVPIIGGVVALLLVV
VVVGVVIWKKKSKKGE
>Sasa-UMAps No prediction chr.14 NC_027313:71.687.592-71.688.277
HISQVM*YGSEWDEETGITDVLFQYGHDREDFISFDLKTMTLVAPKHYPTQRCITFLKKY
LQNGKSTLQRIVSILQKTPSSSVVTCHATGFYPRGVMVMWTRGEQDIYEDVKYGETLPNH
VGTFQMRSRLTVDPKEWDNNNT
>Sasa-UGA XP_013997204.1 chr.14 NC_027313:59.499.424-59.507.750
MKIYFVLLSCIHGALSVIHSLRYFYTSSSGISDFPEFVDMGMVNDQVISHYDSITKRKVP
KQSWMGKVFDQQYWDSTTEDLRGAEKVFKNNLQTAQKRFNQTGGMHISQDMYGCEWDDET
GLTEGFHHIGYDGQDLLVFDLKRATWIASVPQALHSKMKWEGDPSSIESEKRYLTQDCIV
WLKKYLEYGKTTLQRTVPPSVSLLQKTPSSPVTCHATGFYPSGVMVFWQKDGQDHHEDVE
NGETLHNDDGTFQKRTHLKVTSEEWKNNKYQCVVQVTGIKEDFIKVLTESEIQTNRGVNT
IGSAPIIGVVVALLVVVVVVVGLVMWRRKKSKKGFVPASTSDTDSENSGKGAQKI
>Sasa-UHA1 XP_014021022.1 chr.21 NC_027320:48.663.108-48.675.015
MIVLNMYSVSNRIVHISTLLVTYLIPMTFAANHSLKYFYTALPQSTGLPEFSAVAYLDEE
PMYFYDSSTKEVVARQEWVKGAVDPDFWRRNTQILKENEMVFKDNMDTARDRFNQTSALV
LQKMYSCDWDEVTGATDEREQYGYGGEDFLLFDLKNKRWIVPGRQGLITKMKWDANVIKL
EAKIHYLTHTCIEWLKKYVSNWRRNLERTVPPQVSLLQKEPSNPVTCHATGFYPNAIMIF
WGRDGVEIHADVVHEETLPNGDGTYQKRIHLTVSPEDLQQHNYTCTVQHVRGDDVVLSAN
RDSIRSNSRNTQAKTAEMEKPKKRHSADTLQIQLVRIFIPVFV
```

```
>Sasa-UHA2ps no gene prediction chr.21 NC_027320:48.690.859-48.692.163
MGGQILVLWLCLSLRTANSATHSLKYFYTALPQSTGLPEFSAVAYLDEEPMYFYDSSTKE
VVARQEWVKEAVDPDFWRRNTHIFKETEKVFKYNMDSARDRFNQTSALALQKMYSCDWDD
VTGAATDGREQYGYGGEDFLSFDLKNERWIAPGRQGLITKMKWDANVIKLKAKIHYLTHT
CIEWLKKYVSNRRRNLQRTVPPQVSLLQKEPSNPVTCHATGFYPNAIMILWGRDGVEIHD
DVVHEETLPNGDGTYQKRIHLTVSPEDLQQHNYTCTVQHIRGDDVVLSANRDSIRSNSRN
TQGHYITIVLVTLAFSVIVVFIMTKVCKGKEPTAGKEKQCPSEY
>Sasa-ULA XP_014032820.1 chr.27 NC_027326:10.049.215-10.051.213
MKCFILLLLSISLHAASAAMHSLRYVYTATSGMPDFPEFMTVGLVNGEPISYYDSIIRRE
TPRQDWMKEAVDPDYWNRNTQTSIGDEQTFKANIDVAKQRFNQTGGVHVYQNMYGCEWDD
EAGVTEGFDQYGYDGEDFLAFDLKTLKWIAPTPQSLITKLKWDNNMAQIQQDKHYLTQTC
IEWLKKYLDYGKSTLMRTVPPSVSLLQKTPSSPVTCHATGFYPSGVMVSWQKDGQDHHED
VEYGETLQNDDGTFQKSSHLTVTPEEWKNNKYQCVVQVTGVKEDFIKVLTESEIKTNWGN
TNIGFVPANTSDVGSNSSHNTVPKE
>Eslu-UAA XP_028978611.1 chr.10 NC_025977:20.563.916-20.582.608
MKVIILSILGIGFLPTVSAVTHSLKYFYTGVSGDINFPEFTTVGLVDNGQFIYFDSNTKT
AVPKTEWMKREGPDYWDSETQTGINLHQVFKNSIQSTMDRFNQSMSTGVHVVQMMYGCEW
DDETEATEGFDRYGYDGEDFLLLDLKTLSWIAPKSQAFITKNKFDSNKDTNLYLRKYFTQ
TCVDWLKKYVDYGKSSLMRTVPPTVSLLQKTPSSPVTCHATGFYPSRVNVTWKKNGQEQH
EDVEMGEILPNDDGTFQTSVRLNVKPEERKNNKYQCVVQVAGINEDFIKDLTEDTTSIGP
IIGVVVAGLLVIAIVVIGVVIWKKKSKK
>Eslu-UBA XP_012986805.1 chr.10 NC_025977:20.351.381-20.369.310
MYFFVLLILIIGHLHTVSAVTHSLKYFYTGVSGDINFPEFTTVGLVDNVTFIYFDSNTKT
AVPKTEWIKRVGPGYWVSQTKTCINLHQFFKNNIQVAKDRFNQSMSTGVHVLQWMYGCDW
DDETGVIQGFDQYGYDGENFISLDLKTLSWIAPKPQAFITKNKWDSDKADNEYLKHYFTL
ECIEWLKKYLQYEETSQMRTVPPSVSLLQKTPSSPVTCHATGFYPSEVNILWQKDGPFQH
EDVETGHTLPNGDGTFQKSVLLTVKPEEWKNNKYQCVVQVSGINKDFIKDLTESEIQTNR
DTSIGHIITVVVAGLLAIGAAVIGVVNRKKKSKKASDDGSNSSNKSSSNV
>Eslu-UCApt XP_028978614.1 chr.10 NC_025977:20.310.040-20.343.354
MQIDYYDSNTKKVVPKQDWMAKAVGSDYWERQTQNSIGAEQSFKANIDTAKQRFNQTGGV
HVVQEMYGCEWDDETGVIEGFDQHGYDGEDFISFDLKTLKWVAPTPQAFISKNKLDYNEA
GNEGEKHYLTQECIEWLKKYVDYGKSSLMRTVPPSVSLLQKTPSSPVTCHATGFYPSGVH
VAWKKDGQDHHEDVEMGETLQNDDGTFQKSVLLTVKPEEWKNNKYQCVVQVSGINKDFIK
DLTESEIQTNREPTSIGPIIGAVAGLLVIAVATVIGVVIWKKKSKKGFIPANTSDDGSNS
SNNTAPKA
>Eslu-UDA XP_012986807.1 chr.10 NC_025977:20.277.128-20.292.967
MKVIILLILGIGLLPTVSAASHSIRYVYTATSGIPDFPEFVNLGILDGMQIDYYDSNTKK
VVPKQDWMAKAVGSDYWDRETQNSIGREKSFKANIDTAKQRFNQTGGVHVAQMMFGCEWD
DETGVTRGFNQQGFDGEDFISFDLKTLTWIAPKPQAVITKNKWDNNKAGNEYWKNYLTQE
CIDWVKKYVDYEKSSLMRTVPPSVSLLQKTPSSPVTCHATGFYPSGVHVTWKKDGQDHHE
DVEMGETLQNDDGTFQKSVRLNVKPEEWKNNKYQCVVQISGTKEDFIKDLTEDELVSNWG
KTNRGVNDQTSIGPIIGVVVAGLLVIVAAVIIGVVIWKKNRKGFVPANTSDGGSSTNSAP
EA
>Eslu-UEA XP_028978617.1 chr.10 NC_025977:20.168.704-20.174.982
MKVFILLIMGIGLLHSVSAATHSLCYVYTATSGIPDFPEFVTVGLVNREPISYYDSIIRT
ETPRQDWMANNEGSDYWESQTQISNGTEQTFKANIDILKQRFNQTGGVHVVQNMYGCEWD
DETGVTDGFDQYGYDGEDFIVFDLKRLTWIAPKPQAFINKNKWDNDRGWIEQTKNYLTQT
CIEWLKKYVDYGKSSLMRTVPPSVFLLQRTPSSPVTCHATGFYPSRVMVTWMKDGQEQYE
DVEVGETLQNDDGTFQKSVHLTVKPEEWKKNKYQCVVQVSGIKEDFIKDLTEDQIQTNTG
FVPANTSDDGSNSSNNTAPKA
>Eslu-UFA XP_028978615.1 chr.10 NC_025977:20.150.199-20.136.525
MKVFILLIMGIGLLHSVSAATHSLCYVYTATSGIPDFPEFVTVGLVNREPISYYDSIIRT
ETPRQDWMANNEGSDYWESQTQISNGTEQTFKANIDILKQRFNQTGGVHVVQNMYGCEWD
DETGVTDGFDQYGYDGEDFIVFDLKRLTWIAPKPQAFINKNKWDNDRGWIEQTKNYLTQT
CIEWLKKYVDYGKSSLMRTVPPSVSLLQKTPSSPVTCHATGFYPSGVHVTWKKDGQEQHE
DVEMGETLQNDDETFQRSAHLTVKPEEWKNNKYQCVVQVAGIKEDFIKDLTEEEIKTNMD
TTSIGPIIGVVVAGLLVIVAAVIGVVIWKKKSKKGFVPANTSDDGSNSSNNTAPKA
>Eslu-UGApt XP_034147214.1 NW_022995044:9.751-21.223
MQIDYYDSNTKKVVPKQDWMAKAVGSDYWDRETQNSIGREKSFKANIDTAKQRFNQTGGV
HVAQMMFGCEWDDETGVTRGFNQQGFDGEDFISFDLKTLTWIAPKPQAVITKNKWDNNKA
GNEYWKNYLTQECIDWVKKYVDYEKSSLMRTVPPSVSLLQKTPSSPVTCHATGFYPRRVH
VTWKKDGQDHHEDVEMGETLQNDDGTFQTSVHLNVKPEEWKNNKYQCVVQVSGIKEDFIK
DLTEDELLSNWGKTNRGVNDQTSIGPIIGVVVAGLLVIVAAVIIGVVIWKKNRKGFVPAN
TSDGGSSTNSAPEA
```

AF4.2 Deduced Z lineage amino acid sequences

```
>Saal-ZBAa XP_023831639.1 chr.31 NC_036870:10.263.928-10.297.134
MYRPNLMFLVLYFSLECICRSQSDIYSLXYIYTALSKPLELPGIHEFTAMGLMNNRQIDY
YDSVSKKKIPKQEWMREKLPADYWEKGTQSRKSKEQWFKVNVNILMDRMRHNNTDVHILQ
WKHGCEIEQQIDGTLNFIKGTDQYSYDGDDFLAFDDATMQWVAPVDQALPTKRKWDGVQI
LNQYTKGYLEKECVDWLSKFMEYGKKHLRMDDSPPKVYXFAKKAKTAGHVRLTCMATGFY
PKEVEMHIXKNGVPFTKHDGVQSTXXLPNDDDXYQIRMSVQIPEADKETYECSVSHRALK
EPIVVKWDGKCCDCTQVTGVIIGAVVAVVLLLTGVSSLLVLWKRGKIFXGKKGALPPTGI
QAPLNGNGNGAAVLNMPVKKDDASSTSSDSGQGSNERLEREAMLANGGKTRDNPSPLDSG
QGSSESSSEGSCQEGSPTGSREDIDENQQLLPGDGLYISTSLSASIAPFRVWVI
>Saal-ZCAa XP_023831647.1 chr.31 NC_036870:10.307.087-10.314.615
MYRPNLMFFVLYFSLECICRSQSDIYSLNYIYTALSKPLELTGIHEFTAMGLMNDKQIDY
YDSVSKKKIPKQDWMREKLPXDYWEKGTQSRKSKEQWFKVNVNILMDRMRHNNTDLHILQ
WKHGCEIDQQSDGKVKFIKGTDQYSYEGDDFLAFDDVTMQWVAPVDQALPTKRKWDGVQI
LNQYTKGYLEKECVDWLSKFMEYEYKEFSRADSPPKVYAFAKKAKTAGHVRLTCMATGFY
PRDVLMHIKKNGVPLTKHDGVQSTEALPNDDDTYQIRMSVQIPEADKETYECYVDHRTLT
EPIVVKWDGECCDCSSFGAVVIGSVIAIIVVLILLSGVLFVLHKRGTIVIPGLRTTATGN
EVEFSGVNTFENGG
>Saal-ZBAb XP_023830996.1 chr.30 NC_036869:17.481.295-17.482.388 dual orf
MYTFMLIVLYFATECIVQSQSEIYSLNYIYTALSKPVELPGIHEFTAMGLMNDKQIDYYD
SVDKKKIPKQDWMREKLPADYWEKGTQSRKSKEQWFKVNINILMDRMXHNNTDVHVLQWK
HGCEIDKQPDGTLKFIKGTDQYSYDGDDFLAFDFATMQWVASVVQAVPTKRKWDGVQILN
QYTKGYLEKECVYWLSKFMEYGDKEFSSTDPPPNISVFAKKATPAGNVRLTCMATGFYPK
DVIIHIKKNGVQLTEDDGVQSDGVLPNNDDTYQIRISVQIPEADKEMYECSVSHTTLNES
IVKKWDGTCRDCSQSTVAXIAIIIIVGAAAIGIIIVLFLWRK
>Saal-ZCAb XP_023830996.1 chr.30 NC_036869:17.402.402-17.403.594 dual orf
KIYSLNYIYTALSKPVELPGIHEFTAMGLLNDKQIDYYDSVAKKKIPKQDWMREKLPADY
WEKGTQSRKSKEQWFKVNVNILMDRMRHNNTDVHILQWKHGCEIDKQPDDTLKFIKGTDQ
YSYDGDDFLAFDXATMQWVAPVDQALPTKRKWDGVQILNQYTKGYLEKECVDWLSKFMXY
GXKEFXRXDSPPKVYAFAKKAKTAGHVRLTCMATGFYPKDVLMYIKKNGVQLTKQDGVQS
DGVLPNDDETYQIRMSVQIPEADKETYECFVXHTTLKEPIWVKWDGKCCDCSQVTVVVIV
AVVLVVLIVALVTSLLVLWKKGKICAGNTGALXPTEIQASLNGTVDTMSFSFL
>Onne-ZBAa XP_029514088.1 chr.4 NC_042538:45.904.746-45.954.046
MYRPNLIFVLYFSLECICLSQSDIYSLNYIYTALSKPLELPGIHEFTAMGLMNDKQIDYY
DSVAKKKIPKQDWMREKLPADYWDKGTQSRKSKEQWFKVNVNILMDRMMHNNTDVHILQW
KHGCEIDRQSDGTLKFIKGTDQYSYDGDDFLAFDDANMQWVAPVDQALPTKRKWDGVQIL
NQYTKGYLEKECVDWLSKFMKYEDKEFSRADSPPKVYVFAKKAKTAGHVRLTCMATGFYP
KDVEMHIKKNGVPLTERDGVQSTGVLPNDDDTYQIRMSVQIPEADKETYECYVDHRTLKK
PIVGKWDGICCDCSSFGAVVIGAVIAIIVVLILISVVLFVLHKSGTIVIPGLKTTATGNG
VAFSGVNTL
>Onne-ZCAa XP_029514085.1 chr.4 NC_042538:45.921.305-45.927.348
MCAFKMYVVVLLFLFATLSTVDSVEKWSLNYIYTALSKPVELPGIHEFTAMGLMNDKQID
YYDSVAKKKIPKQDWMREKLPADYWDKGTQSRKSKEQWFKVNVNILMDRMRHNNTDVHIL
QWKHGCEIDRQSDGTLKFIKGTDQYSYDGDDFLAFDDANMQWVAPVVQAQPTKRKWDGVQ
ILNQYTKGYLEKECVDWLSKFMAYEDKEVSRADSPKVYAFAKKAKTAGNVRLTCMATGFY
PKDVEMHIKKNGVPLTERDGVQSTGILPNDDDTYQIRMSVQIPEADKGTYECSVNHVALK
EPVVVKWDGKCCDCTQVTGVVIGAVVAVVLLLIVFSSMLVLWKKGKIFAGTKGDLPATGI
QAPLNVTKDDASSTDSGQGSNESLEKRQCCQVEPLRSD
>Onne-ZDAa XP_029514089.1 chr.4 NC_042538:45.945.026-45.954.046
MYRPNLMFFVLYFSLECICRSQSDIYSLNYIYTSLSKPLELPGIHEFTAMGLMNDKQIDY
YDSVAKKKIPKQAWMREKLPADYWDKGTQSRESKEQWFKVNVDILMKRMMHNNTDVHILQ
WKHGCEIDQQSDGTLKFIKGTDQYSYDGDDFLAFDDANMQWVAAVVQAQPTKRKWDGVQI
LNQYTKGYLEKECVDWLSKFMKYEDKEFSRADSSPRVYVFAKKAKTAGHVRLTCMATGFY
PKDVIMQIKKNGVPLTERDGVQSTGVLPNDDDTYQIRMSVQIPEADKETYECYVDHRTLK
KPIVVKWDGECCDCSSGTAVVIGAVIAIIIVVLILI
>Onne-ZBAb XP_029519275.1 chr.7 NC_042541:17.154.314-17.164.103
MYTFILFVFYFSTECIFQSQSEIYSLNYIYTALSKPVELPGIHEFTAMGLMNNRQIDYYD
SVDKKKIPKQDWMREKLPADYWEKGTQSRKSKEQWFKVNVDILINRMRHNNTDVHILQWR
HGCEIDKQPGGILKFIKGTDQYSYDGDDFLAFDDATMQWVAPDDQALPTKRKWDGVQILN
QYTKGYLEKECVDWLSKFMAYGDKEFSKADSPPNIYVFANKVKTAGNVRLTCMATGFYPK
DVIINFKKNGIQLNKNDGVLSTGARPNNDDTYQIRISVQIPEADKQTYECFVNHITLNES
```

IVEKWDGTCRDCSQSTVAIIAIIIIGGAAAIGIIIVLFLWKKGKICAGNTGALPPTGIQA
PLNGSG
>Onne-ZCAb XP_029519273.1 chr.7 NC_042541:17.135.412-17.150.806
MYFVVVLLLFGTLSTVNSETLSLSYIYTALSKPVELPGIHEFTAMGLMNNRQIDYYDSVD
KKKIPKQDWMREKLPADYWEKGTQSRKSKEQWFKVNVDILMNRMRHNNTGVHILQWRHGC
EIDKQPDGTLKFIKGTDQYSYDGDDFLAFDDATMQWVAPVDQALPTKRKWDGVQILNQYT
KGYLEKECVDWLSKFMAYGDKEANKHSPPKVYAFAKKAKIAGHIRLTCMATGFYPKDVLM
HIKKNGVQLTKQDGVQSDGVLPNDEESYQIRMSVQIPEADKETYECFVNHRTLKEPIVVK
WDGKCCDCSQGTVAVIVAVVVAVVLLLAMVTLLLVLWRTGTICAGQTGALMPTGNQAPLN
VTKDVASSTSSDSGQGSNERLERETMLTNGVTEDASSTSSDSGQGSNERLERETMLTNGV
TTEDASSTSSDSAIGE
>Onki-ZBAb XP_020355968.1 chr.14 NC_034187:56.393.574-56.407.374
MYTFILFVFYFSTECIFQSQSEIYSLNYIYTALSKPVELPGIHEFTAMGLMNNRLIDYYD
SVDKKKIPKQDWMREKLPADYWEKGTQSRKSKEQWFKVNVDILMNRMRHNNTDVHILQWR
HGCEIDKQPGGTLKFMKGTDQYSYDGDDFLAFDDAAMQWVAPVDQALPTKRKWDEVQILN
QYTKGYLEKECVDWLSKFMEYGDKEFSKADSPPNIYVFANKAKTAGDVRLTCMATGFYPK
DVKINFKKNGIQLNKNDGVLSTGARPNNDDTYQIRISVQIPEADKQTYECFVNHITLNES
IVEKWDGTCRDCSQSTVGIIAIIIIGGAAAIGIIIVLFRWKKGKICAGNTGALPPTGIQA
PLNVTMNGARGTSADSGKDGGSINSSDSGKGSQKERSSDGASEEEVKTPMLPN
>Onki-ZCAb XP_020355967.1 chr.14 NC_034187:56.408.644-56.418.129
MYFVVVLLLFGTLSTVNSETLSLNYIYTALSKPVELPGIHEFTAMGLMNNRQIDYYDSVD
KKKIPKQDWMREKLPADYWEKGTQSRKSKEQWFKVNVDILMNRMRHNKTGVHILQWRHGC
EIDKQPDGTLKFIKGTDQYSYDGDDFLAFDDATMQWVAPVDQALPTKRKWDGVQILNQYT
KGYLEKECVDWLSKFMTYEDKEANKHSPPKVYAFAKKAKTAGHIRLTCMATGFYPKDVIM
HIKKNGVQLTKQDGVQSDGVLPNDEESYQIRMSVQIPEADKETYECFVNHRTLKEPIVAK
WDGKCCDCSQGTVVVIVAVVAVVLLLAVVTSLLVLWRIGTICGGQTGALMPTGNQAPLNV
TTEDASSTSSDSGQGSNERLERETMLTNGVTTEDASSTSSDSGQGSN
>Onki-ZBAa_pt XP_031649466.1 chr.17 NC_034190.2:33.300.332-33.308.914
MGLMNDKQIDYYDSVAKKKIPKQAWMREKLPADYWDKGTQSRKSKEQWFKVNVDILMKRM
RHNNTDVHILQWKHGCEIDRQSDGTLKFIKGTDQYSYDGDDFLAFDDANMQWVAPVVQAQ
PTKRKWDGVQILNQYTKGYLEKECVDWLSKFMNYEDKEFSRADSPPRVYVFAKKAKTAGH
VRLICMATGFYPKDVIMQMKKNGVPLTERDGVQSTGVLPNDDDTYQIRMSVQIPEADKET
YECSVDHRTLKAPIVGKWDGICCDCSSFGAVVIGAVIAIIVVLILVSVVLFVLHKSGTIV
IPGLKTTDQGSQNAESSDRGSEEDVKKSMLPQENDRFWPNFLTGP
>Onki-ZCAa XP_031649459.1 chr.17 NC_034190.2:33.287.633-33.293.654
MCAFKMYVVVLLFIFATLSTVDSVGKWSLNYIYTALSKPVELPGVHEFTAMGLMNDKQID
YYDSVSKKKIPKQDWMREKLPADYWDKGTQSRKSKEQWFKVNINILMDRMRHNNTDVHIL
QWKHGCEIDQQSDGTLKFIKGTDQYSYDGDDFLAFDDANMQWVAPVVQAQPTKRKWDGVQ
ILNQYTKGYLEKECVDWLSKFMAYEDKEVSRADPPKVYAFAKKAKTVGNVRLTCMATGFY
PKDVEMHIKKNSVPLTERDGVQSTGVLPNDDDTYQIRMSVQIPEADKETYECSVDHVALK
KPVVVKWDGKCCDCTQVTGVIIGAVVVLVLLLTVVSSMLVLWKKGKIFAGKKGDLPATGI
QAPLNGNGNGAAVLNMPGKRDDASSTDSGQGSNESLEKEAILPNGAIEE
>Onki-ZDAa XP_020361083.2 chr.17 NC_034190.2:33.261.064-33.268.451
MYRPNLMFFVLYFSLECICRSQSDIYSLNYIYTSLSKPLELPGIHEFTAMGLMNNQQIDY
YDSVAKKKIPKQAWMREKLPADYWDKGTQSRKSKEQWFKVNVDILMKRMMHNSTDVHILQ
WKHGCEIDQQSDGTLKFIKGTDQYSYDGDDFLAFDDANMQWVAPVVQAQPTKRKWDGVQI
LNQYTKSYLEKECVDWLSKFMKYEDKEFSRADSPPRVYAFAKKAKTAGHVRLTCMATGFY
PKDVIMQIKKNGVPLTEHGVQSTGVLPNDDDTYQIRMSVQIPEADKETYECYVNHRTLKK
PIVVKWDGECCDCSSGTAVVIGAVIAIIVVLILVSVVLFVLHKRETIVIPGLKTTATGNG
VAFSGVNTLEYGG
>Onts-ZBAa XP_024297365.1 chr.13 NC_037109:25.649.700-25.659.903
MYRPNLMFFVLYFSLECICLSQSDIYSLNYIYTALSKPLELPGIHEFTAMGLMNDKQIDY
YDSVAKKKIPKQDWMREKLPADYWDKGTQSRKSKEQWFKVNVDILMKRMRHNNTDVHILQ
WKHGCEIDQQSDGTLKFIKGTDQYSYDGDDFLAFDDANMQWVAPVVQAQPTKRKWDGVQI
LNQYTKGYLEKECVDWLSKFMKYEDKEFNRADSPPRVYEFAKKAKTAGHVRLTCMATGFY
PKDVIMQIKKNGVPLTERDGVHSTGVLPNDDDTYQIRMSVQIPEADKETYECYVDHRTLK
KPIVVKWDGECCDCSSGTAVVIGAVIAIIVVLILISVVLFVLHKSGTIVIPGLKTTAKYD
ESRTSSDSDQGSQNAGSSGRGSEEDVKKSMLPQESV
>Onts-ZCAa XP_024297360.1 chr.13 NC_037109:25.633.867-25.641.623
MCAFKMSVFVLLFLFATLSTVDSVGKWSLNYIYTALSKPVELPGIHEFTAMGLMNDKQID
YYDSVSKKKIPKQDWMREKLPADYWDKGTQSRKSKEQWFKVNVNILMDRMRHNNTDVHIL
QWTHGCEIDQQSDGTLKFIKGTDQYSYDGEDFLAFDDANMQWVAPVVQAQPTKRKWDGVQ
ILNQYTKGYLEKECVDWLSKFMAYEDKEVSRADSPKVYAFAKKAKTAGNVRLTCMATGFY

PKDVIMQMKKNGVPLTERDGVQSTGVLPNDDATYQIRMSVQIPEADKETYECSVNHVALK
EPVVVKWDGKCCDCTRVTGVIIRAVVVVVLLLTVVPCCSMLVLWKKGKIFAGKKGDLPAT
GIQAPLNGNGNGAAVLNMPGTRMTLAALTLAASNV
>Onts-ZDAa XP_024297354.1 chr.13 NC_037109:25.712.62-25.77467
MYRPNLMFFVLYFSLECICRSQSDIYSLNYIYTSLSKPLELPGIHEFTAMGLMNNQQIDY
YDSVAKKKIPKQAWMREKLPADYWDKGTQSRKSKEQWFKVNVDILMKRMMHNSTDVHILQ
WKHGCEIDQQSDGTLKFIKGTDQYSYDGDDFLAFDDANMQWVAPVVQAQPTKRKWDGVQI
LNQYTKGYLEKECVDWLSKFMKYEDKEFSRADSPPRVYEFAKKAKTAGHVRLTCMATGFY
PKDVIMQIKKNGVPLTERDGVQSTGVLPNDDDTYQIRMSVQIPEADKETYECYVDHRTLK
KPIVVKWDGECCDCSSGTAVVIGAVIAIIVVLILISVVLFVLHKSGTIVFPGLKTTATGN
GVAFSGVNTLEYANLISPLL
>Onts-Z1_pt XP_024256643.1 NW_020128813:6.533.170-6.542.805
MGLMNNRQIDYYDSVDKKKIPKQDWMREKLPADYWEKGTQSRKSKEQWFKVNVDILMNRM
RHNNTDVHILQWRHGCEIDKQPGGTLKFMKGTDQYSYDGDDFLAFDDATMQWVAPVDQAL
QTKRKWDEVQILNQYTKGYLEKECVDWLSRFMEYGDKEFSKADSPPNIYVFANKAKTAGN
VCLTCMATGFYPKDVIINFKTNGIQLNKNDGVLSTGARPNNDDTYQIKISVQIPEADKQT
YECFVNHRTLNESIVEKWDGTCRDCSQSTVGIIAIIIIGGAAAIGIIIVLFRWKEGKICA
GNTGALPPTGIQAPLNGSGIGATNSNLTTTPMTMNGARGTSSDSGKDGGSINSSDSGKGS
QKEGLQMEPVRRKLRHRCCLIEVA
>Onts-Z2 XP_024256647.1 NW_020128813:6.544.708-6.554.336
MYFVVVLLLFGTLSTVNSETLSLNYIYTALSKPVELPGIHEFTAMGLMNNRQIDYYDSVD
KKKIPKQDWMREKLPADYWEKGTQSRKSKEQWFKVNVDILMNRMRHNNTGVHILQWRHGC
EIDKQPDGTLKFIKGTDQYSYDGDDFLAFDDATMQWVAPVDQALPTKRKWDGVQILNQYT
KGYLEKECVDWLSKFMTYEDKEANKHSPPKVYAFAKKAKTAGHIRLTCMATGFYPKDVIM
HIKKNGVQLTKQDGVQSDGVLPNDEESYQIRMSVQIPEADKETYECFVNHRTLKEPIVVK
WDGKCCDCSQVTVVVIVAVVAVVLLLAVVTSLLVLWRIGTICAGQTGALMPTGNQAPLNV
TKDGASSTSSDSGQSSNECLEREKMLTNGVTTEDASSTSSDSGQGSNERLESDVMLTNEA
TGE
>Onts-ZBAb_pt XP_024251602.1 chr.31 NC_037127:26.757.171-26.766.798
MGLMNNRQIDYYDSVDKKKIPKQDWMREKLPADYWEKGTQSRKSKEQWFKVNVDILMNRM
RHNNTDVHILQWRHGCEIDKQPGGTLKFMKGTDQYSYDGDDFLAFDDATMQWVAPVDQAL
QTKRKWDEVQILNQYTKGYLEKECVDWLSRFMEYGDKEFSKADSPPNIYVFANKAKTAGN
VCLTCMATGFYPKDVIINFKTNGIQLNKNDGVLSTGARPNNDDTYQIKISVQIPEADKQT
YECFVNHRTLNESIVEKWDGTCRDCSQSTVGIIAIIIIGGAAAIGIIIVLFRWKEGKICA
GNTGALPPTGIQAPLNGSGIGATNSNLTTTPMTMNGARGTSSDSGKDGGSINSSDSGKGS
QKEGLQMEPVRRKLRHRCCLIEVAR
>Onts-ZCAb Z XP_024251600.1 chr.31 NC_037127: 26.745.632-26.755.261
MYFVVVLLLFGTLSTVNSETLSLNYIYTALSKPVELPGIHEFTAMGLMNNRQIDYYDSVD
KKKIPKQDWMREKLPADYWEKGTQSRKSKEQWFKVNVDILMNRMRHNNTGVHILQWRHGC
EIDKQPDGTLKFIKGTDQYSYDGDDFLAFDDATMQWVAPVDQALPTKRKWDGVQILNQYT
KGYLEKECVDWLSKFMTYEDKEANKHSPPKVYAFAKKAKTAGHIRLTCMATGFYPKDVIM
HIKKNGVQLTKQDGVQSDGVLPNDEESYQIRMSVQIPEADKETYECFVNHRTLKEPIVVK
WDGKCCDCSQVTVVVIVAVVAVVLLLAVVTSLLVLWRIGTICAGQTGALMPTGNQAPLNV
TKDGASSTSSDSGQSSNECLEREKMLTNGVTTEDASSTSSDSGQGSNERLESDVMLTNEA
TGE
>Onmy-ZBAb XP_021416205.1 chr.18 NC_035090:28.089.685-28.101.281
MYTFILFVFYLSTECIVQSQSVIYSLNYIYTALSKPVELPGIHEFTAMGLMNNRQIDYYD
SVDKKKIPKQDWMRDKLPADYWEKGTQSRKSKEQWFKVNVDILMNRMRHNNTDVHILQWR
HGCEIDKQPDGTLKFIKGTDQYSYDGDDFLAFDDANMQWVAPVDQALPTKRKWDRVQILN
QYTKGYLEKECVDWLSKFMTYGDKEFSKADSPPNIYVFANKAKTAGNVRLTCMATGFYPK
DVIINFKKNGIQLNKNDGVLSTGARPNNDDTYQIRMSVQIPEADKQTYECFVNHITLNES
IMEKWDGTCHDCSQSTDAIIAIIIGGAAAIGIIIVLFR
>Onmy-ZCAb XP_021416204.1 chr.18 NC_035090:28.076.769-28.087.832
MYFVVVLLLFGTLSTVNSETLSLNYIYTALSKPVELPGIHEFTAMGLMNNRQIDYYDSVD
KKKIPKQDWMRDKLPADYWEKGTQSRKSKEQWFKVNVDILMNRMRHNNTGVHILQWRHGC
EIDKQPDGTLKFIKGTDQYSYDGDDFLAFDDATMQWVAPVDQALPTKRKWDGVQILNQYT
KGYLEKECVDWLSKFMEYGDKEANKHSPPKVYAFAKKAKIAGHIRLTCMATGFYPKDVLM
HIKKNGVQLTKQDGVHSDGVLPNDEESYQIRMSVQIPEADKETYECFVNHRTLKDPIVVK
WDGKCCDCSQGTVVVIVAVVAVVLLLALVTSLLILWRIGTICAGQTGALKPTGNQAPLNV
TKDGASSTSSDSGQGSIERLERETMLTNGVTKDGASSTSSDSGQGSNERLESEVMLTNEA
TGE
>Onmy-ZDAb-ps No prediction chr.14 NC_035090.1:28.075.382-28.075.939
VHLLMWKHGCKIDQQSDGTLKFIYGTDQYSYDGDDFLAFDDATMQWVAPVDQALPTKRKW

DGVQILNQYTKGYLEKECVDWVSKFMAYGGKVFSRADKKSQTAGHVRLT*MATGFYPKE
>Onmy-ZBAa XP_021427706.1 chr.18 NC_035094:33.630.377-33.638.372 two genes
MYRSNFMFFVLYFSLECICLSQSDIYSLNYIYTALSKPLELPGIHEFTAMGLMNDKQIDY
YDSVAKKKIPKQDWMREKLPADYWDKGTQSRKSKEQWFKVNVDILMKRMRHNNTDVHILQ
WKHGCEVDQQSDGTFKFIKGTDQYSYDGDDFLAFDDANMQWVAPVVQAQPTKRKWDGVQI
LNQYTKGYLEKECVDWLSKFMAYEDKEFSRADSPPRVYVFAKKAKTAGHVRLTCMATGFY
PKDVEMHIKKNGVPLTERDGVQSTGVLPNDDDTYQIRMSVQIPEADKETYECYVDHRTLK
KPIVGKWDGICCDCRSFGAVVIGAVIAIIVVLILISVVLFVLHKRGTIVIPGLRTRATDD
ESRTSSDSDQGSQNAGSSERGSEEDVKSMLPQGMEKWS
>Onmy-ZCAa XP_021427706.1 chr.18 NC_035094:33.647.289-33.649.964 two genes
LNYIYTALSKPVELPGIHEFTAMGLMNDKQIDYYDSVSKKKIPKQDWMREKLPADYWDKG
TQSRKSKEQWFKVNINILMDRMRHNNTDVHILQWKHGCEIDRQSDGTFKFIKGTDQYSYD
GDDFLAFDDANMQWVAPVVQAQPTKRKWDGVQILNQYTKGYLEKECVDWLSKFMAYGGKE
VSRASPPRVYAFAKKAKTAGHVRLTCMATGFYPKYVIMQMKKNGVPLTKRDGVQSTGVLP
NDDDTYQIRMSVQIPEADKETYECSVNHVALKEPVVVKWDGKCCDCTQVTGVIIGAVVVL
VLLLTAVSSMLVLWKKGKIFAGKKGDLPATGIQAPLNGNGNGTAVLNMPGQGSNKSLERE
AMLANGGTKDGASSTDSGQGSNERLEREAMLANGVIKDGASSTDSGQGSNASLEERQC
>Onmy-ZDAa XP_021427698.1 chr.18 NC_035094:33.686.393-33.693.710
MVVDRPSLQAAGVSFHQQDQEIYSLNYIYTSLSKPLELPGIHEFTAMGLMNNQQIDYYDS
VAKKKIPKQAWMREKLPADYWDKGTQSRKSKEQWFKVNVDILMKRMMHNSTDVHILQWKH
GCEIDRQSDGTLKFIKGTDQYSYDGDDFLAFDDANMQWVAPVVQAQPTKRKWDGVQILNQ
YTKGYLEKECVDWLSKFMAYEAKEFSRADSPPRVYAFAKKAKTAGHVRLTCMATGFYPKD
VIMQIKKNGVPLTERDGVQSTGVLPNDDDTYQIRMSVQIPEADKETYECYVDHRTLKKPI
VVKWDGECCDCRSFGAVVIGAVIAIIVVLILVSVVLFVLHKRGTIVIPGLRTRATGNGVA
FSGVNTLEYGG
>Satr-ZAAa XP_029587655.1 chr.34 NC_042990:10.854.743-10.873.281
MNISHLTVFVLCFSLECICQSDTYSLSYIYTALSKPVDLPGIHEFTAMGLMNNKQIDYYD
SVSKKKIPKQDWIREKLPADYWEKGTQSRKSKEQWFKVNVDILMKRMRHNNTDVHVLQWK
VGCEIDQQSDGTLKFIKGIDQYSYDGDDFLAFDDVTMQWVAPVDQALPTKRKWDGVQILN
QYTKGYLEKECVDWLSKFMEYEDKEFSWADSPPKVYAFAKKAKTAGHVQLTCMATGFYPK
DVVMHIKKNEVLLTKHDGVQSAGVLPNDDETFQIRMSVQIPEADKETYECYVHHRALKEP
IVVKWDGKCCDCSSVGAVVIGAVVIAFIVVLILVVLFVLHRRGTIGIPGLRTRANGNGAA
VMV
>Satr-ZBAa XP_029587649.1 chr.34 NC_042990:10.993.167-11.002.989
MYRPNLMFFVLYFSLECICRSQSDIYSLNYIYTALSKPVELPGIHEFTAMGLMNNKQIDY
YDSVSKKKIPKQDWMREKLPADYWEKGTQSRKSKEQWFKVNVNILMERMRHNDTDLHILQ
WKHGCEIDQQSNGTLKFIKGTDQYSYDGDDFLAFDDVTMQWVAPVDQALPTKRKWDGVQI
LNQYTKGYLEKECVDWLSKFMEYEDKEFSRADSAPKVYAFAKKAKTAGHVRLTCMATGFY
PKDVVMRIKKNAVPLTEHDGVQSAGVLPNDDETYQIRMSVQIPEADKETYECYVNHRPLE
EPIVVKWDGTCCDCSSFGAVVIGAVVIAFIAVLILVSVVLFVLHRRGTIVINGLRTRAKD
DESRTSSDSGKASQNTGSSDRGSEEDVKKSMLPKGSG
>Satr-ZCAa XP_029587646.1 chr.34 NC_042990:11.010.935-11.018.246
MCAFKMYVVVLLFLFATLSTVDSVERWSLNYIYTALSKPVELPGIHEFTAMGLMNNQQID
YYDSVAKKKIPKQDWMREKLPADYWEKGTQSRKSKEQWFKVNVNILMDRMRHNNTDLHIL
QWKHGCEIEQRDGTLKFIKGTDQYSYDGDDFLAFDDVTMQWVAPVDQALPTKRKWDGVQI
LNQYTKGYLEKECVDWLSKFMEYGEKHLRMDDSPPKVYAFAKKAKTAGHVRLTCMATGFY
PKDVVMRIKKNAVPLTEHDGVQSAGVLPNDDETYQIRMSVQIPEADKETYECYVNHRALK
EPIVEKWDGKCCDCNQVTGVIIGAVVAVVLLFITSLLVLWKKGKIFAGKKGAPPPTGIQE
PLNGNGFGAAVLNMPVKKDDASSTDSGQGSNERLETEPMLANGVKKDDASSTDSGQGSNE
RLETEPMLTNGVKKDDASSTDSEPLGS
>Satr-ZDAa XP_029587654.1 chr.34 NC_042990:11.044.071-11.050.979
MYRPNLMFFVLYFSLECICRSQSDIYSLNYIYTALSKPVDLPGIHEFTAMGLMNNQQIDY
YDSVSKKKIPKQDWMREKLPADYWEKGTQSRKSKEQWFKVNVNILMERMRHNNTDVHILQ
WKHGCEIDQQSDGTLKFIKGTDQYSYDGDDFLAFDDATMQWVAPVDQALPTKRKWDGVQI
LNQYTKGYLEKECVDWLSKFMEYGEKEFSRADSAPKVYAFAKKAKTAGHVRLTCMATGFY
PKDVEMRIKKNAVPLTEHDGVQSAGVLPNDDETYQIRMSVQIPEADKETYECYVHHRTLK
GPIVVKWDGICCDCSSGGAVVIGAVIIIIVVLILVSVVLFVLHRRGTIVIPGLRTTAIGN
GVAFSGVNTS
>Satr-ZBAb XP_029591369.1 chr.36 NC_042992.1:32.133.017-32.147.191
MYTFMLFVFYLSTECIVQSQSEIYSLNYIYTALSKPVELPGIHEFTAMGLMNNRQIDYYD
SVDKKKIPKQDWMREKLPADYWEKGTQSRKSKEQWFKVNVNILMERMRHNNTGVRILQWK
HGCEVDKQPDGTLKFIKGTDQYSYDGDDFLAFDDVTMQWVAPVDQALPTKRKWDGVQILN
QYTKGYLEKECVDWLSKFMEYGEKHFSSADSPPIINVFANKAKTAGNVHLTCMATGFYPK

DVIIHFKKNGVQLTEDDGVLSTGARPNNDDTYQIRISVQIPEADKQTYECFVNHITLKEQ
IVVKWDGTCRDFSQSTVPIIAIIIIGGSVAIGIIILLFLWKKGKICAGNTGAPPPTGIQA
SLIGNGNGATNLILTTTPMTTDGASSTSSDSGQGSNEHPEREAMLTNGVGEDGGSSNSSD
SGKGSQKERSSDGASEEEVKTPMLPN
>Satr-ZCAb XP_029591372.1 chr.36 NC_042992.1:32.120.133-32.131.498
MYFVVVLLLFGTLSTVNSEIHSLNYIYTALSKPVELPGIHEFTAMGLMNDKQIDYYDSV
DKKKIPKQDWMREKLPADYWEKGTQSRKSKEQWFKVNVNILMERMRHNDTGVHILQWRHG
CEVDTQPDGTLKFIKGTDQYSYDGDDFLAFDDVTMQWVAPVDQALPTKRKWDGVQILNQY
TKGYLEKECVDWLSKFMAYGDKELIRADSPPKVYAFAKKAKTAGHIRLTCMATGFYPKDV
LMHIKKNGVQLTKQDGVLSDGVLPNDDKTYQIRMSVQIPEADKETYECYVSHRMLKEPIE
VKWDGKCCDCSQVTVVVIVAVVVVVLLLALVTSLLVLRKKGKICAGNTGALPPTEIQAPL
NGNGNGAAILSMPVTMDSASSTSSDSGQGSNERLEREAMLTNEAIGE
>Satr-ZDAbpt XP_029591820.1 chr.36 NC_042992.1:32.117.738-32.119.137
MLWALTNNSYDGDDFLAFDDATMQWVAPVDQALPTKWKWDRVQILNQYTKGYLEKECVDW
VSKFMVYGDKEFSPPKIYVFANKAKTAGNVHLTCMATGFYPKDVIIHIKKNGVHLTDKDG
VQSSVVIPNDDDAYQIRKSVEIQEADTSNYKCYVLHGTLLKPIVGKWGKVF
>Sasa-ZAAa XP_014032772.1 chr.27 NC_027326:10.464.841-10.482.971
MNISHLTVFVLYFSLECICQSDTYSLSYIYTALSKPVDLPGIHEFTAMGLMNNQQIDYYD
SVSKKKIPKQDWMREKLPADYWEKGTQSRKSKEQWFKVNVDILMKRMRHNNTDVHVLQWK
VGCEIDQQSDGTLKFIKGIDQYSYDGDDFLAFDDVTMQWVAPVDQALPTKRKLDDVQILN
TYTKGYLEKECVDWLSKFMEYEDKEFSWADSAPKVYAFAKKAKTAGHVRLTCMATGFYPK
DVVMHIKKNGVPLTDRDGVQSAGLLPNDDETYQIRMSVQIPEADKETYECYVHHRTLEEP
IVVKWDGKCCDCSSGGAVVIGAVVITFIVVLILVGLFVLHRRGTIVIPGLRTRANGNGAA
AMV
>Sasa-ZBAa XP_014032768.1 chr.27 NC_027326:10.602.279-10.612.437
MYRPNLMFFVLYFSLECICRSQSDIYSLNYIYTALSKPVDLPGIHEFNAMGLMNNKQIDY
YDSVSKKKIPKQNWMREKLPADYWEKGTQSRKSKEQWFKVNVNILMDRMRHNNTNVHILQ
WKHGCEIDQQRDGTVKFIKGIDQYSYDGDDFLAFDDVTMQWVAPVDQALPTKRKWDGVQI
LNQYTKGYLEKECVDWLSKFMEYGDKEFSRADSAPKVYAFAKKAKTAGHVRLTCMATGFY
PKDVEMNIKKNGVPLTKHDGVQSAGVLPNDDETYQIRMSVQIPEADKETYECYVHHRTLE
EPIVIKWDGKCCDCSSFNAVVIGAVVITFIVVLILVVLFVLHRRGTIVIHGLRTRAKDDE
SRTSSDSGKGSQNAGSSDSGSGEDVKKSMLPKGSV
>Sasa-ZCAa XP_014032767.1 chr.27 NC_027326:10.620.148-10.627.552
MSAFKMYVVALLLLFATLSTEDTVETWSLNYIYTALSKPVELPGIHEFTAMGLMNDKQID
YYDSVAKKKIPKQDWMREKLPADYWEKGTQSRKSKEQWFKVNVNILMDRMRHNNTDVHIL
QWKHGCEINQQSDGTLKFIKGTDQYSYDGDDFLAFDDVTMQWVAPVDQALPTKRKWDGVQ
ILNQYTKGYLEKECVDWLSTFMEYGKKHLRMDDSAPKVYAFAKKAKTAGHVRLTCMATGF
YPKDVVMHIKKNGVPLTKHDGVQSAGVLPNDDESYQIRMSVQIPEADKETYECYVYHRTL
EEPIVEKWDGKFYDCNQVTGVIIGVVAVVLLFIVVTPLLVLWKKGKIFAGKKGAPPPTGI
QEPLNVKKDDASSTDSGQGSNEYLETEPMMTTGAVGE
>Sasa-ZDAa XP_014032770.1 chr.27 NC_027326:10.648.408-10.655.356
MYRPNLMFFVLFFFLECICRSQSHIYSLNYIYTALSKPVDLPGIHEFTAMGLMNNQQIDY
YDSVSKKKIPKQDWMREKLPADYWEKGTQSRKSKEQWFKVNVNILMDRMRHNNTDVHILQ
WKHGCEIDQQSDGTLKFIKGTDQYSYDGDDFLAFDDVTMQWVAPVDQALPTKRKWDGVQI
LNQYTKGYLEKECVDWLSKFMEYGEKEFSRPDSAPKVYAFAKKAKTAGHVRLTCMATGFY
PKDVVMHIKKNGVPLTDRDGVQSAGVLPNDDETYQIRMSVQIPEADKETYECYVHHRTLE
KPIVIKWDGICCDCSSFNAVVIGAVVITFIVVLILVVLFVLHRRGTIVIPGLRTTATGNG
VAFSGVNTS
>Sasa-ZBAb XP_013997187.1 chr.14 NC_027313:59.698.589-59.724.523
MYTFMLFVFYFSTECIVQSQSEIYSLNYIYTALSKPVELPGIHEFTAMGLMNDIQIDYYD
SVDKKKIPKQDWMREKLPADYWEKGTQSRKRKEQWFKVNVNILMERMRHNNTDVHVLQWR
HGCEVDKQPDGTLKFMKGIDQYSYDGDDFLAFDDVTMQWVAPVDQALPTKRKWDGVQILN
QYTKGYLEKECVDWLSKFMEYGEKHFSSTDSPPNIYVFTKKAKPAGNVHLTCMVTGFYPK
DVIIHFKKNGVQLTEDDGVLSTGARPNNDDTYQIRISVQIPEADKDMYECSVSHAMLKEP
IVEKWGAGNTGAPPPTGIQASLIGNGNGATNLNLTTTPIGEDGGSSNSSDSGKGSQKERS
SDGASEEEVKTPMLLN
>Sasa-ZCAb XP_013997181.1 chr.14 NC_027313:59.735.522-59.758.314
MYTFMLFVFYFSTECIVQSQSEIYSLNYIYTALSKPVELPGIHEFTAMGLMNNRQIDYYD
SVDKKKIPKQDWMRDKLPADYWEKGTQSRKSKEQWFKVNVNILMERMRHNNTGVRILQWK
HGCEVDKQPDGTLKFIKGTDQYSYDGDDFLAFDDVTMQWVAPVDQALPTKRKWDGVQILN
QYTKGYLEKECVDWLSKFMEYGEKHFSSADSPPDINVFANKAKTAGNVHLTCMATGFYPK
DVIIHFKKNGVQLTEDDGVLSTGARPNNDDTYQIRISVQIPEADKQTYECSVSHITLVQP
IVVKWDGTCRDFSQSTVPIIAIIIIGGSVAIGIIIVLFLSKKGKICAGNTGAPPPTGIQA

SLIGNGNGATNLNMIYTPSNGNGATNYNMIHTPSNGNGATNYNMIHTPSNGNGATNCNMI
>Sasa-ZDAb XP_013997179.1 chr.14 NC_027313:59.767.933-59.779.225
MYFVVVLLLFGTLSTVNSEIHSLNYIYTALSKPVELPGIHEFTAMGLMNDKQIDYYDSVD
KKKIPKQDWMREKLPADYWEKGTQSRKSKEQWFKVNVNILMERMRHNNTDVHILQWRHGC
EVDTQPDGTLKFMKGTDQYSYDGDDFLAFDDVTMQWVAPVDQALPTKRKWDGVQILNQYT
KGYLEKECVDWLSKFMAYRDKEFIRADSPPKVYAFAKKAKTAGHIRLTCMATGFYPKDVL
MHIKKNGVQLTKQDGVQSDGVLPNDDESYQIRMSVQIPEADKETYECYVSHRTLKEPIEV
KWDGKCCDCSQVTVVVIVAVVVVVLLLALVTSLLVLRKKGKICAGNTGALSPTEIQAPLN
GNGNGAAILSMPVTMDSA
>Eslu-ZAA XP_010898810.3 chr.10 NC_025977:19.692.986-19.710.404
MINNMNKFSFLFFVATGYIVPSQCEIYSLNYIYTALSKPVELPGIHEFTAMGLMNDKQID
YYDSDIKQKIPRQDWMKEKLPPDYWEKGTQSRKSKEQWFKVNVNILMERMKHNKTDVHIL
QWKHGCEVDKQSDGTLKFIKGTDQYSYDGDDFLAFDDVTSQWVAPVDQALPTKRKWDGVQ
ILNQYTKGYLEKECVDWLSKFMSYEHTEFSSTDAPPKVFAFAKKAKTEGHVRLTCMATGF
YPKDVQMQIMKNGVPLTEHDGVYSEGVLPNNDDTYQIRKSVQIPEADTNDYECHVDHRTL
TEPFVKKWDGICCDCGINIGAIIGPAVGVVFPLLFLLIFVVLWKIHIGRLPWAPVPQNVY
GNEMTNTPGDSDGDGVN
>Eslu-ZBA XP_028978590.1 chr.10 NC_025977:19.050.846-19.111.940
MVNNMYMFSYLFLVATGYIFQSQCEIYSLNYIYTALSKPVELPGIHEFTAMGLMNDRQID
YYDSDVKQKIPRQDWMKEKLPPDYWEKGTQSRKSKEQWFKVNVNILMERMKHNNTDVHIL
QWKHGCEVDKQSDGTLSFIKGTDQYSYDGDDFLAFDDVTSQWVAPAVQALPTKRKWDGVQ
ILNQYTKGYLEKECVDWLSKFVSYGHETFSSADNPPKIYGFAKKAKTKGQLQLTCMATGF
YPKDVEMQIRKNGVPLTKYDGVPSEGVLPNNDDTYQIRMSVQIPEADTHDYECHVDHRTL
KEPIRKKWDGKCCDCGPNIGIIIGAVGVVVVLLFLLILILVLWKTNNICIRRQTFKDQKP
QDPQNGQTLHNVYSEGGVKQNMLPNGSG
>Eslu-ZCA XP_028978597.1 chr.10 NC_025977:19.042.390-19.048.486
MYKLSFLFFVAMAYIVQNQCERYSLNYIYTALSKPVELPGIHEFTAMGLMNDRQIDYYDS
DVKQKIPKQGWMKEKLPPDYWEKGTQSRKSKEQWFKVNVNILMERMRHNNTDVHILQWKH
GCEIDEQNNGTMRFIKGTDQYSYDGDDFLSFDDATSQWVAPAVQALPTKRKWDGVQILNQ
YTKGYLEKECVDWLSKFMSYGEQELKKASPPKVYAFAKKARTEGHVRLTCMATGFYPKDV
EMQIRKNGVPLTKDDGVHSEGVLPNNDETYQIRKSVQIPEADTNDYECHVDHRTLTEPQV
VKWDGKCCDCSSSKGVIIGSIVGVVVVVLLIIILIVVLWKRLRDNGSRPWSPVSQNGYING
GTNTPVYANGGTGTPA
>Eslu-ZDA XP_028978589.1 chr.10 NC_025977:19.031.923-19.039.410
MYFIVVLLLVGTLSTVDSERYSLNYIYTALSKPVELPGIHEFTAMGLMNDRQIDYYDSDV
KQKIPKQGWMKEKLPPDYWEKGTQSRKSKEQWFKVNVNILMERMKHNNTDVHILQWKHGC
EVYKQNDGTFQFIKGTDQYSYDGDDFLAFDDATSQWVAPVDQAQLTKRKWDGVQILNQYT
KGYLEKECVDWLSKFMSYGDQELKKASPPKVYAFAKKARNEGHVRLTCMATGFYPKDVEM
QIRKNGVPLTKDDGVHSEGVLPNNDETYQIRKSVQIPEADTNDYECHVDHRTLTEPQVVK
WDGKCCDCSSSIGVIIGSTVGVVLVVVPLLILIVVLWKRNRVTMNCDSSSTSSESGNGSK
VSLETEAILTNAVNNDVSSTSTSDSGQASNERLEMLRN
>Eslu-ZEA XP_028978588.1 chr.10 NC_025977:19.020.042-19.028.369
MYFLMLLLFLGALSKVDSDRWSLNYIYTALSKPVELPGIHEFTAMGLMNDKQIDYYDSDV
KQKIPRQGWMKEKLPPDYWEKGTQSRKSKEQWFKVNVNILMERMKHNNTDVHILQWKHGC
EVDKQNDGTLRFIKGTDQYSYDGDDFLSFDDLTSQWVAPAVQALPTKRKWDGVQILNQYT
KGYLEKECVDWLSKFMSYGHQEFSSVDSPPKVYAFAKKARTEGHVRLTCMATGFYPKDVE
MQIRKNGVPLTKDDGVHSEGVQPNNDETYQIRMTVQIPEADTNDYECHVDHRTLTEPFVK
KWDGKCCDCSQSTVVIIGAVVCVVVLLAIFIAFLVFLWKKGIIGGFFRLY

AF4.3 Deduced L lineage amino acid sequences

>Saal-LLAps No prediction NW_019943469:81.560-81.919 pseudogene
IKTFLEKEKKCIMHKVXPRVRLIQKAMSGAIQQVSCLAFGFYPRHINLTLLRDSQPIAEQ
ALSGGQLLPNGDGTYQLRKSLEVSTEELXERHNYTCTASHLSLDNKLDVSWVPESGADRS
>Saal-LCA XP_023998402.1 NW_019944620:29.783-37.832
MDKLSVFLFVLSFCTIVNAGSGSHSLWALATYIRGETPFPEFTVVVMLDDVQVAYYDSNM
KHFIYRGHNTSDKIHDDEAKNGGFVFGVMYHHMKERYFHLKHHLNLTEGVQVQQRMAGCE
MLDNGEPALIVTKNIVNAVFVDHAIYYNMTHFTYDAGKLLQGWGGMRQAQEKFLYENVLL
PLCIRTLKTILKREKNVVMRKVPPRLRLINKELSGGLQVSCLAFGFYPRHINLTLLXDGQ
PVTEQELTGGEVLPSGDGTYQLRKSLEVSTEELKKRHNYTCTASHLSLDNKLDVSWESGA
ERVHLSTLSVLLVMLLIVILLGIFICVKRRRSN
>Saal-LGA2pt XP_023998403.1 NW_019944620:41.294-41.896
MMQEYERKVVENVCLPICIKTMKTFLKREKNIVMCKVPPRLRLIKKEVSGGLQVSCLAFG

FYPRHINLTLLRDGQPVAEQELTGGQVLPSGDGNYQLRKSLEVSTEELKKRHNYTCTASH
LSLDNKLDVSWESGAERVHLSTLSVLLVMLLIVILLGIFICVKRRRSNTASQLKLANVDA
NVSEEMILSSTSEDT
>Saal-LEA XP_023998401.1 NW_019944620:51.869-53.649
MGKLSVLLFVLSFSTIVNAGSGSHSLWALATYISGETPFPEFTVVVMLDDVQVAYYDSNI
KHFIYRVNNTPNKIHDNEAQDGAYILGMMYNNMKERSFNLKHHFKFTEGVQVQQRMSGCE
ILDNGEPVMFMVKDTFNAIYTDQMVYYNMTHFIYDAGKLLLGSDGIRQAFERVLFENVHL
PICIRTLKTILKREKNIVMRKVPPRLRLINKEVSGGFQVSCLVFGFHPRHINLTLLRDSQ
PVAEQELTGGEVLPSGDGTYQLRKSLEVSTEELKKRHNYTCTASHLSLDNKLDVSWESGA
ERVHLSTLSVLLVMLPIVILLGIFICVKRRWRCTASQSKLTNVDANVSEEINLSSDSET
>Saal-LHA1ps No prediction NW_019945788:11.961-12.656 pseudogene
HILCGTCNIYSGETPFPEFTVVVMLDDVQVAYYGLHMENFIYRATHQTKRG**EAQDGAH
VFVDMYQSMMRQII*TKAPL*SSGRCQVQQKMADCEMLDNANQPWFMSKDTTNGISADMH
YITT*HIYIRCWDTTT*DGWHNASIWKDTLENVYIPFASEH*RNISEERKNVCDA*SSSQ
TQVDKERGFCRAPGELPGVCFLPPHIQPDLLRDGQPVTEQS*QGGQVLPSGE
>Saal-LHA2ps No prediction NW_019946183:39.211-39.759 pseudogene
GVQVQQRMAGCEMLDNGEPAMIMSKNTFNAIYTDRT*FSTQHIYI*CWETTRMGWDEQAF
QNTFD*WLLPCIKH*KTFLKREKNCDA*SSSQTQVDKKRGFWRAPGELPGVWFLPPPHQP
DPAERRPASGRTGADRGGVLPSGDGDYQLRKSLEVSTEELKKRNSYTCTASHLSLDTKLD
VRW
>Saal-LBAps No prediction NW_019946183:39345-40.025 pseudogene
SGSGSHSLWALATBIXGETPFPEFTVVVMFDDVQVGYYDSNMKEFIYSGLNPTDKIHDDV
AQDGAYVFGTIVPRYKERLQLKHHCNLTEVFKFSKEWLAVRCWTMVNQP*SCQRTLSMPF
IQIERNFQHNTFTYD-AGKLLGWDGMSKHFRTLLINGYFHASNTEKHS*RERRIVMRKVP
PRLRLIKKEVSGGLQVSCLAFGFYPRHINLTLLRDGQPVAEQELTGGE
>Saal-LNApt No prediction NW_019946183:48.240-48.899
SGPGSHSLWALATYIIGETPFPEFTSVGLLDDVQVGYYDSKDKLSVYRGLNPTDKMYDDL
AQEGDRVFETMYQDMKERAFQLKHHFNLTGGVQVQQRMAGCEMLDNGEPAMIMYKETFNA
IYTDQTVISNTTHFTYDAGKLLLGWDGMMQDFKRTLYINVHLPRCIRTLKKFLKREKKVV
MRKVPPRLRLIKKEVSXGLQVSCLAFGFYPRHINLTLLRD
>Saal-LDA XP_023853355.1 chr.1 NC_036838.1:57.299.152-57.301.114
MGKLSIFLFVLSFYTIVNAGSGSHSLWALATYIIGETPFPEFTVVLMLDDVQIGYYDSNI
KQSVYRGYHITDKMNDEAQDGTYVLGTMYHHMKERSFRLKHHLNLTEGVHVQQRIGGCEI
LHNGEPALIMTKNSFNAIFADYAVYYNMTHFTYDSGKLLLGYNWMRQATERTLYANVWIN
TLKHTICINTLKKCLNRENFVMRRVPPRLRLIKKEVSGDLQVICLAFGFYPRHINLTLLR
DGHPVAEQELTGGEVLPSGDGTYQLRKSIYVSTEELRERHNYTCTASHLSLNNKLDVSWE
SGAERVHLFILSAPLVMALIVILFCILICLTRRIRAAS
>Saal-LAA1ps No prediction chr.17 NC_036857:19.496.374-19.499.780 pseudogene
IITGIFVHQRRIGCELVDNEKQGQLMIKEVFNGIDGGVLNFNKLQYNYHPMWPELEFNQQ
RTQYVQMGLDKVYFPICIK*LKDYLKKEEKLVMRKVSPRVRFI*KETQILKGPRTPAWPV
YHRHINLTLLRDGQPVAEHELKGRQLLPN*DWTYQLRKSLTITVQEL*ERHNYTCTASPV
SMDNKLDVSWPDTSPDSASIIPVVLVMAVVLILIGSLVVFGMWKWKHAGFPPFSGHIYSV
AKDTETEQSNSSLETESTDS
>Saal-LAA2ps No prediction chr.7 NC_036847:21.281.746-21.283.178 pseudogene
VLSILDHVQMGYYDSNAWICISKTDGNSDEAFEGVAQITLWDVFLIMRGIFVNQRLCGCE
LMDNXKQGQIMIKESFNGFDGALNFNKLQYHPKWPELAFNQHRPQYIQMGLGKVHLPICI
KTLKDYLKKGEMLVMRAFGFYPCHINLTLLRNGQPVAEHDLTGGQLLPNGDRL*QLRMNL
TINAQELRERGRDIITPVLPLICLDNKLXVSW
>Saal-LIAps No prediction chr.36 NC_036875:17.167.958-17.168.785 pseudogene
WAFATCISGEAPFPECSVVLMQDDIQVGYFDSNMEQFIHKGPYAPDETEVDVAQDAAYVF
GHMFLSMKRRLSDLRYRFNSTGNIDVQQRMTGCEMLETGEPGLILSTDAFNAIFADLIYY
NMTHYSYNSGNLLSPWSEMHQTYAKWRYQTIYLPICIKTLKKYLE*LKNFVMRKVRPRVR
LIQKAMSGGARVSCLAFGFYPRHINLTLLRDGQPIVEQEMTGGQLLPNGDGTYQLRKSLE
VNTEELRERHNYTCTTSHLSLDNKLDVSWIPESGID
>Saal-LJAps No prediction chr.2 NC_036839:31.412.441-31.413.246 pseudogene
TYIIGDTPFPEYTVVLMLDDIEVGYYEWSNVLQHERQII*LKQRFNFTEGIHVQQRMAGC
EMLDNDKMVLTLSRSTFNKTVADGMCYNMTQNTYYTGNPQLAWDEVKLEYVKMLYAHVYL
TLCIKTLKIFLERENNNLMRKVRPRVRLIKKAKSGGLQVSCLAFGFYPRHINVTLLRDGQ
PIAEKELTGGQLLPNGDGTYQLRKSLEIIAEDLRERHNYTCTVSHLSLDNKLDASWVPEP
GRD
>Onne-LAA1pt XP_029477974.1 chr.20 NC_042554.1:16.723.950-16.727.606
HVLGETEFPEFCVLWMLDDVQVGYYDSKSWRFISRTDGNIDEEYSRTVQGASWDVYLSMR
KWSSLLQHRFNSTTGIFVHQRLIGCELVDNEKQGQLMIKEAFNGIDGGVLNFNKLQYNYH

PKWPELEFNQQRTQYVQMGLDKVYLPICIKSLKDYLKKEEELVMRKVRPRVRLISKESTD
TAGAKITCLAFGFYPRHINLTLLRDGQPVAEHELKGGGAIATEGDWTYQLRKSLTITIQE
LRERPNYTCTANHISMDNKLDVSWGVPTFSGHIYSAAKVTETEQSNSSLETEPTDS
>Onne-LAA2ps No prediction chr.7 NC_042541:47.191.684-47.192.995 pseudogene
FPESCVVSILDNVQMGYYDSNAWICISKTDGISDEAFEGVAQNTFWDMFLSMRKQASFLL
HHFNNGIFVNLRRCGCELLDNNKQGKIMIKESFNGFDGALNFNKLQYHPKWPELAFNQHL
ENLHLPICIKTLKDYLTKGEMLVMRRV*KKTLGIQQEQIYSGSIDTHQAFGFYPCHINLT
LLRNGQPVAEHDLTGGQ
>Onne-LJAps No prediction chr.3 NC_042537:50.850.799-50.851.083 pseudogene
LIQKVMSVGLQMSCLVFGFNPRHTNLTLLRDGQLKAEPEMTGGQLPPNGDWVY*LRKRLE
AITEELRERHNYTCTVSHLSLDNKLDVSWEPDPDR
>Onne-LIA XP_029493803.1 chr.1 NC_042535:19.342.746-19.344.526
MVKLCFFWLFLSLYTIINAGSHSLLAFATCISGEAPFPECSVVLMQDDIQVGYFDSNTEQ
FIHKGPYAPDETEVDLAQDAANVFGHMFLSMKRRLLDLRYRFNSTGNIDVQQRMAGCEML
DTGEPGLILSTDAFNAILADLIYYNMTHYSYNSGNLLSPWSEVHQTYAKWRYQTIYLPIC
IKTLKKFLERLKNFVMRKVRPRVRLTQKAMSGGARVSCLAFGFYPRHINLTLLRDGQPIV
EQDITGGQLLPNGDGTYQLRKSLEVNTEELRERHNYTCTTSHLSLDNKLDVSWIPESGID
RVGLYVKSGPLGMVAIIIILLSIFVCVRRRNAAGSQTLSQLSNANDAQVAEQMSLSSHSET
>Onne-LGA2 XP_029512659.1 NW_021815573:18.384-20.495
MGKLSVFLFVLSFYTIVNAGSGSHSLWALATYISGETPFPEFTVVVMLDDVQVGYYDSNM
KQSVYRGQHINDKAEDEEVQDGALVFGDMYQSMKNRLFKLKHHFNLTEGVQVQKRIAGCE
MLDNGEPALVMFKNVFNTVSVDYAIYYNMTHFTYDAGQLLQGWDGKRKEVEINNLMEVYL
PICIKSLKRFLKREKNIVMRKVPPRLRLIKKEVSGGFQVSCLAFGFYPRHINMTLLRDGQ
PVAEQELTGGEVLPSGDGTYQLRKSLEVSTEELKKRHNYTCTASHLSLDNKLDVSWESGA
ERVHLSTL
>Onne-LNAps No prediction NW_021815399:7.214-7.912
IMYHHMKGRSFHLKHHFNLTGGVQVQQRMSGCEMLDSGEPALIMEKDTFNAIYTDQTLYY
NMTHFTYDAGKLRPGYDGVRREYLRTLFGNVFLPICIRTLKTILKREKNVVMRKVPPQTQ
VDKERGFWRVSGELPGVWFLPPPHQLKKRHNYTCTASHLSLDYKLDVSWESGAERVHPST
LSVLLVMLLILILLGIFICVKWRRRTASQT*SQLATVDAKVEG*INLSSDSKT
>Onne-LCA1 XP_029511433.1 NW_021813807.1:20.949-23.822
MGKRSVFLFVLSFCTIIKSGSGSHSLWALATYISGETPFPEFTVVGMLDDVQVTYYDSNM
KHFIYRGHNTSDKIHDDEAKNGDFVFGVMYHHMKERYFHLKHHLNLTEGVQVQQRMAGCE
MFDNGEPALIMTKNTFNAVFVDHAIYYNMTHFTYDAGKLLQGWDGMRQLQEKILYENVLL
TLCIRTLKTILKREKNVVMRKVPPRLRLIKKEVSGRFQMSCLAFGFYPRHINMTLLRDGQ
PVTEQELTGGEVLPSGDGTYQLRKSLEVSTEELKKRHNYTCTASHLSLDNKLDVSWESGT
ERVHLFTLSVLLVMLLVVILLGIFICVKRRWSNTASQSKLVNVDATVSEEMNLSSDSET
>Onne-LGA1 XP_029511432.1 NW_021813807:23.923-27.281
MRGYTIKIMGKLSVFLFVLSFYTIVNAGPVSHSLWALATYITGETPFPEFTVVLMLDDVQ
VGYYDSNDKQSVYRGQHITEKNKDDEAQDGAYVFGVIYQSMKDRSFELKHHFNLTEGVQV
QQKLTGCEMLDNGEPALVMFKDAFNAIYTDRTLYYNMTHFTYDAGKLLLGWDGIRQAYER
TLYENVYLPICIKSLKKILKREKNVVMRKVPPRLRLIKKEVSGGFQMSCLAFGFYPRHIN
MTLLRDGQPVAEQELTGGEVLPSGDGTYQLRKSLEVSTEELKKSHNYTCTASHLSLDNKL
DVSWESGAERVHLSTLSALLVMLLIVIILGIFICVKRRWSNTASQSKLVNVDATVSEEMI
LSSDSEI
>Onne-LEApt No prediction NW_021797834:685-1.552
LWVLATYISGETPFPEFTVVVMLDDVQVAYYDSNMKHFIYRVNNNPNKMHDNEAQDGAYI
LGMMYNNMKERSFHLKHHFNLTEGVQVQQRMSGCEMLDNGEPVMFMVKDTFNAIRQAFER
VLFENVHLPICIRTQKKLLKREKNIVMREVPPRLRLIKKEVSGGFQMSCLAFGFYPRHIN
LTLLRDGQPVAEQELTGGEVLPSGDGTYQLRKSLEVSTEELKKRHNYTCTASHLSLDNKL
DVSWESGT
>Onne-LCA2pt XP_029504626.1 NW_021794521:1.422-1.907
MRKVPPRLRLIKKEVSGGFQVSCLAFGFYPRHINMTLLRDGQPVAEQELTGGEVLPSGDG
TYQLRKSLEVSTEELKKRHNYTCTASHLSLDNKLDVSWESGTERVHLSTLSALLVMLLVV
ILFSIFICVKRRWSNTASQSKLANIDATVSEKTNLSSVSET
>Onne-LLApt XP_029503466.1 NW_021791591:126.624-134.137
MTSLKARSSELKRFNSTKGIHVQQRLAGCEMSDNGEPALYADLAIYSNMTHYSYNSGNLL
PPWSKVHQTNVKGLYQTIYLNICMDTLKTFLEKEKKCIMHKVRPRVRHIQKAMSXDGSSS
LAIGFYPRHIGQPIAEQALNRGQLLPNGDDTYQLRKSLEVSTEELRERRNYTCTASHLSL
DNKLDVSWVPESGADRSTILSALLVMVSITILICISVCLRRRREAGSQTLSQLSKPC
>Onne-LCA3ps No prediction NW_021798966:217-910 pseudogene
LCNITPLPGSGSHSLWALAT*TAIGETPFPELRLW*CWMMFKWVTMTPTINSLSTGDNIS
TTKLRMRKLRTELMYLE*YTRGNLNLR*FELKHHFNLTEGL*VQQRLTDCEMMDNGEPVL

AVFKDVFNGIFQDMYYNMTHYTYDAGKLLLGRDGLMQVYKIITFYLPLCIKSLKRFLKRE
KNVVMREVPPRLRLIKKEVSGGFQVSCLAFGFYPRHINMTLLRDGQPVGEQE
>Onne-LDA XP_029502641.1 NW_021789945:33.648-35.270
MGKLSIFLFVLSFYTIVNAGSGSHSLWALATYIIGETPFPEFTVVLMLDDVQIGYYDSNM
KLSVYRGYHITDKMNDEAQDGTYVLGTMYHHMKERSFRLKHHLNLTEGVHVQQRIGGCEI
LHNGEPALIMTKNSFNGIFADYAVYYNMTHFTYDSGQLLLGYNWMRQATERTLYANVWLP
ICINTLKKCLNREKNFVMRRVPPRLRLIKKEVSGDLQVICLAFGFYPRHINLTLLRDGHP
VAEQDLTGGEVLPSGDGTYQLRKSIYVSTEELREKHNYTCTASHLSLNNKLDVSWESGAE
RVHLFILSAPLVMALIVILFCILICLARRIRAASQNLQLASVDALEADERNLPSDSEI
>Onne-LCA4pt XP_029501170.1 NW_021786625:5-1.037
MGKLSVFLFVLSFCTIVKSGSGSHSLWALATYISGETPFPEFTVVGMLDDVQVTYYDSNM
KHFIYRGHNTSDKIHDDEAQDGAYVFGIMYHHMKERYFHLKHHLNLTEGVQVQQRMAGCE
MLDNGEPALIMTKNIFNAVFADHAIYYNMTHFTYDAGQLLQGWDGIRQAYQRTVYENFYV
PICIRTLKRFLKREKNVVMRKVPPRLRLIKKEVSGGFQVSCLAFGFYPRHINMTLLRDGQ
PVA
>Onki-LEA1 XP_020329286.2 NW_022263489:10.300-12.741
MGKLSVLLFVHSFSTIVNAGSGSHSLWVLATYISGETPFPEFTVVVMLDDVQVAYYDSNM
KHFIYRVNNTPNKMHDNEAQDGAYILGMMYNNMKERSFNLKHHFNLTEGVQVQQRMSGCE
MLDNGEPVMFMVKDTFNAIYTDQMVYYNMTHFIYDAGKLLRGWDGMRQAFERVLFENVHL
PICIRTQKTLLSREKNVVMRKVPPRLRLIKKEVSGGFQVSCLAFGFYPRHINMTLLRDGQ
PVAEQELTGGEVLPSGDGTYQLRKSLEVSTEELKKRHNYTCIAFHLSLDNKLDVSWESGT
ERVHLSTLSVLLVMLLVVILLGIFICVRRWSNTASQSKLVNVDAKVSEEMNLSSVSEA
>Onki-LCA1 XP_031674528.1 NW_022263489:44.278-46.380
MGKLSVFLFVLSFCTIVKSGSGSHSLWALATYISGETPFPEFTVVVMLDDVQVTYYDSNM
KHFIYRGHNTSDKIHDDEAQDGAYVFRIMYFHMKERYFHLKHHLNLTEGVQVQQRMAGCE
MFDNGEPALIMTKNTFNAVFVDHAIYYNMTHFTYDAGQLLQGWDGMRQLQEKILYENVLL
TLCIRTLKTILKREKNVVMRKVPPRLRLIKKEVSGGFQVSCLAFGFYPRHINMTLLRDGQ
PVAEQELTGGEVLPSGDGTYQLRKSLEVSTEELKKRHNYTCTASHLSLDNKLDVSWESGA
ERVHLSTLSVLLVMLLVVILLGIFICVKGRWSNTASQSKLVNVDATVSEEIILSSDSET
>Onki-LCA2 XP_031675082.1 NW_022263912:18.540-21.867
MGKLSVFLFVLSFCTIVKSGSGSHSLWALATYISGETPFPEFTVVVMLDDVQVTYYDSNM
KHFIYRGHNTSDKIHEDEAQDGAYVFRIMYHHMKERYFHLKHHLNLTEGVQVQQRMAGCE
MFDNGEPALIMTKNTFNAVFVDHAIYYNMTHFTYDAGQLLQGWDGMRQLQEKILYENVLL
TLCIRTLKTILKREKNVVMRKVPPRLRLIKKEVSGGFQVSCLAFGFYPRHINMTLLRDGQ
PVAEQELTGGEVLPSGDGTYQLRKSLEVSTEELKKRHNYTCTASHLSLDNKLDVSWESGT
ERVHLSTLSVLLVMLLVVILLGIFICVKGRWSNTASQSKLVNVDATVSEEIILSSDSET
>Onki-LGA2.1 XP_020329298.2 NW_022263912:27.797-30.086
MGKLSVFLFVLSFYTIVNAGSGSHSLWALATYINGETPFPEFTVVVMLDDVQVTYYDSND
KQSVYRGQHITDKTEDDDAQDEAYIFGDMYQSMKDRSVELKHHLNLTEGVQVQQRLTGCE
MLDNGEPALAMFKNIFNGISEDYALYYNTTHFTYDAGKLLLGWDGIMQTLMKTKFKNFYL
PICIKNLKRFLKREKNVVMRKVPPRLRLIKKEVSGGFQVSCLAFGFYPRHINMTLLRDGQ
PVAEQELTGGEVLPSGDGTYQLRKSLEVSTEELKKRHNYTCTASHLSLDNKLDVSWESGT
ERVHLSTLSVLLVMLLVVILLGIFICVKRRWSNTASQSKLANIDATVSEEMILSSDSET
>Onki-LGA2.2 XP_031676068.1 NW_022265080:6.822-9.114
MGKLSVFLFVLSFYTIVNAGSGSHSLWALATYINGETPFPEFTVVVMLDDVQVTYYDSND
KQSVYRGQHITDKTEDDDAQDEAYIFGDMYQSMKDRSVELKHHLNLTEGVQVQQRLTGCE
MLDNGEPALAMFKNIFNGISEDYALYYNTTHFTYDAGKLLLGWDGIMQTLMKTKFKNFYL
PICIKNLKRFLKREKNVVMRKVPPRLRLIKKEVSGGFQVSCLAFGFYPRHINMTLLRDGQ
PVAEQELTGGEVLPSGDGTYQLRKSLEVSTEELKKRHNYTCIAFHLSLDNKLDVSWESGA
ERFHLSTPSVLLVMLLIVILLGIFICVKRRWSNTASQSKLANIDATVSEEMILSSDSET
>Onki-LNA XP_020330717.2 NW_022261980:3.615.302-3.617.889
MGKLSVFLFVLSFSTIVNAGSGSHSLWALANYIIGETPFPEFTAVVMLDDIHVVYYDSND
KQPVYRGPKPNGKIHDEVVQDGDYVFGIMYHHMKGRSFHLKHHFNLTGGVQVQQRLTGCE
MLDNGEPALIMEKDTFNAIYTDQTLYYNMTHFTYDAGKLRPGYDGVRREYLRTLYGNVFL
PICIRTLKRILKREKNVVMRKVPPRLRLIKKEVSGGFQVSCLAFGFYPRHINMTLLRDGQ
PVAEQELTGGEVLPSGDGTYQLRKSLEVSTEELNKRHNYTCTAFHLSLDNKLDVSWESGA
ERVHLSTLSVLLVMLLILILLGIFICVKGRRRTASQT
>OnkiLEA2 XP_031668801.1 NW_022261980.1:4.277.226-4.279.638
MGKLSVLLFVHSFSTIVNAGSGSHSLWVLATYISGETPFPEFTVVVMLDDVQVAYYDSNM
KHFIYRVNNTPNKMHDNEAQDGAYILGMMYNNMKERSFNLKHHFNLTEGVQVQQRMSGCE
MLDNGEPVMFMVKDTFNAIYTDQMVYYNMTHFIYDAGKLLRGWDGMRQAFERVLFENVHL
PICIRTQKTLLSREKNVVMRKVPPRLRLIKKEVSGGFQVSCLAFGFYPRHINMTLLRDGQ
PVAEQELTGGEVLPSGDGTYQLRKSLEVSTEELKKRHNYTCIAFHLSLDNKLDVSWESGT

ERVHLSTLSVLLVMLLVVILLGIFICVRRWSNTASQSKLVNVDAKVSEEMNLSSVSET
>Onki-LFA XP_031668802.1 NW_022261980.1:4.293.378-4.296.692
MAKLSVLFFILFYTIGNAGSGSHSLWALATYINGETPFPEFTVVVMLDDVQVAYYDSNMK
HFIYRVNNTPNKMHDDEAQDGAYVFGIIYQSMKERYFHLKHHLNLTEGVQVQQRMSGCEM
LDNGEPALIMFKETFNGIFVDHAIYNSMTHFTYDSGTLLVGYYGIRQAYEKALFENVLLP
ICIKSLKRFLKREKNVVMRKVPPRLRLIKKEVSGGFQVSCLAFGFYPRHINMTLLRDGQP
VAEQDLTGGEVLPSGDGTYQLRKSLEVSTEELKKRHNYTCTASHLSLDNKLDVSWESGAE
RVHLSTLSVLLVMLLIVILLGMFICVMRWRCTASHLELSLQKRERK
>Onki-LGA1 XP_031668490.1 NW_022261980.1:4.306.192-4.309.687
MGKLSVFLFVFSFYTIVNAGSGSHSLWALATYITGETPFPEFTVVLMLDDVQVAYYDSND
KQSVYRGQHITEKNKDDEAQDGAYVFGVIYQSMKDRSAKLKHHLNLTEGVQVQQKITGCE
MLDNGEQALVMFKDVFNAIYTDRTLYYNMTHFTYDAGKLLLGWDGMRQAYERTLYENVYL
PICIKSLKKILKREKNVVMRKVPPRLRLIKKEVSGGFQVSCLAFGFYPRHINMTLLRDGQ
PVAEQELTGGEVLPSGDGTYQLRKSLEVSTEELKKRHNYTCTPSHLSLDNKLDVSWESGA
ERVHLSTLSVLLVMLLVVILLGIFICVKRRWSNTASQSKLVNVGATVSEEMILSSDSEI
>Onki-LDA XP_020350446.1 chr.1 NC_034174:12.150.462-12.152.115
MLHFKIMGKLSIFLFVLSFYTIVNAGSGSHSLWALATYIIGETPFPEFTVVLMLDDVQIG
YYDSNMKLSVYRGYHITDKMNDEAQDGTYVLGTMYHHMKERSFRLKHQLNLTEGVHVQQR
IGGCEIRHNGEPALIMTKNSFNGIFADYAVYYNMTHFTYDSGQLLLGYNWMKQATDRTLY
ANVWLPICINTLKKCLNREKNFVMRRVPPRLRLIKKEVSGDLQVICLAFGFYPRHINLTL
LRDGHPVAEQELTGGEVLPSGDGTYQLRKSIYVSTEELREKHNYTCTASHLSLNNKLDVS
WESGAERVHLFILSAPLVMALIVILFCILICLARRIRAASQNLQLASVDALEADERNLSS
DSEI
>Onki-LIA No prediction chr.26 NC_034199:18.984.593-18.983.787
AFATCISGEAPFPECSVVLMQDDIQVGYFDSNTEQFIHKGPYAPDETEVDVAQDAANVFG
HMFLSMKRRLSDLRYHFNSTGNIDVQQRMAGCEMLDTGEPGLILSTDAFNAILADLIYYN
MTHYSYNSGNLLSPWSEVHQTYVKWHYQTIYLPICIKTLKKFRERLKNFAMRKVRPRVRL
TGARVSCLAFGFYPRHINLTLLRDGQPIVEQDMTGGQLLPNGDGTYQLRKSLEVNIEELS
ERHNYTCTTSHLSLDNKLDVSWIPESGID
>Onki-LAA1pt No prediction chr.24 NC_034197:17.676.225-17.679.167
LATHVLGETEFPEFCVLWMLDDVQVGYYDSNSWRFISRTDGNIDEEYSRTVQGASWDVYL
SMRKWSSLLQHRFISTTGLIGCELVDNEKQGQLMKKEAFNGIDGGVRHINLTLLRDGQPV
AEHELKGGQLLPGAIATEGDWTYQLRKSLTITVQELRERQQAVCVPDTGPDSASIIPVVL
VMAVVLILIGILVVFGMWKWKHTG
>Onki-LAA2ps chr.17 No prediction NC_034190:65.180.493-65.181.877 pseudogene
FPEFCVVSILDNVQMGYYDSNAWICISKTDGISDEAFEGVAQNTFWDMFLSMRKQASFLL
HHFNNGIFVNLRRCGCELLDNNKQGKIMIKESFNGFDGALNFNKMQYHPKWPKLACNQHR
TQYIQMGLGKASPPICIKTLKDYLKKGEMLVMRRAFGFYPCHINLTLLRNGQPVAEHDLT
GGQLLPNGDRL*QLRMSLTISAQELRE
>Onki-LLApt No prediction chr.2 NC_034175:69.317.237-69.317.864
SLKARSSELKRFNSTKGIHVQHMLAACEMSDNGEPALIISKDSFNGIYADLAIYSNMTHY
SYNSGNLLPPWSKVHQTYVKGLYQTIYLNMATLKTFLEKEKKCIMHKVPPRVRIIQKLAF
GFYPRHINLTLLRDGQPIAEQALNTRQLLPNGDGTYQLRKSLEVSTEELRERRNYTCTAS
YLSLDNKLDVNWVPECEADRS
>Onts-LDA XP_024239748.1 chr.2 NC_037098:52.252.264-52.253.911
MLHFKIMGKLSIFLFVLSFYTIVNAGSGSHSLWALATYIIGETPFPEFTVVLMLDDVQIG
YYDSNMKLSVYRGYHITDKMNDEAQDGTYVLGTMYHHMKERSFRLKHHLNLTEGVHVQQR
IGGCEILHNGEPALIMTKNSFNGIFADYAVYYNMTHFTYDSGQLLLGYNWMRQATERTLY
ANVWLPICINTLKKCLNREKNFVMRRVPPRLRLIKKEVSGDLQVICLAFGFYPRHINLTL
LRDGHPVAEQELTGGEVLPSGDGTYQLRKSIYVSTEELREKHNYTCTASHLSLNNKLDVS
WESGAARVHLFILSAPLVMALIVILFCILICLARRIRAASQNLQLASVDALEADERNLSS
DSEI
>Onts-LAA1pt XP_024239354.1 chr.22 NC_037118:18.137.866-18.149.768
MKSIQEQFKVRLGTCTSVWRKWSSLLQHRFNSTTVHPRVRLISKESTDTAGAKITCLAFG
FYPRHINLTLLRDGQPVAEHELKGGQLLPXGDWTYQLRKSLTITVQELRERPDYTCTANH
ISMDNKLYVSWVPDTGPDSASIIPVVLVMAVVLILIGILV
>Onts-LAA2ps no prediction chr.16 NC_037112:54.025.063-54.026.488 pseudogene
FPEFCVVSILDNVQMGYYDSNAWICIPKTDGISDEAFEGVAQNTFWDMFLSMRKQASFLL
HHFNNGIFVNLRHCGCELLDNNKQGKIMIKESFNGFDGALNFNKLQYHPKWPELAFNQHR
TQYIQMGLGKASPPAFGFYPCHINLTLLRNGQPVAEHDLTGGQLLPNGDRL*QLRMSLTI
SAQELRERERERHNYTCTATHLS
>Onts-LIA XP_024244907.1 chr.26 NC_037122:19.640.524-19.641.996
MVKLCFFWLFLSLYTTINAGSHSLLAFATCISGEAPFPECSVVLMQDDIQVGYFDSNTEQ

FIHKGPCAPDETEVDVAQDAANVFGHMFLSMKRRLSDLRYRFNSTGNIDVQQRMAGCEML
DTGEPGLILSTDAFNAILADLIYYNMTHYSYNSGNLLSPWSEVHQTYAKWRYQTIYLPIC
IKTLKKFLERLKNFVMRKVRPRVRLTQKAMSGGARVSCLAYGFYPRHINLTLLRDGQPIV
EQDMTGGQLLPNGDGTYQLRKSLEVNTEELRERHNYTCTTSHLSLDNKLDVSWIPESGID
SVGLYVKSGPLGMVAIIILLSIFVCVRRRNAAGSQTLSQLSNANDAQVAEQMSLSSHSET
>Onts-LNA1 XP_024264664.1 NW_020130735:43.853-46.470
MGKLSVFLFVLSFSTIVNAGSGSHSLWALANYIIGETPFPEFTVVVMLDDIHVAYYDSND
KQPVYRGPKPTGKIHDELFQDGDYVFGIMYHHMKGRSFHLKHHFNLTGGVQVQQRMSGCE
MLDNGEPALIMEKDTFNAIYTDQTLYYNMTHFTYDAGKLRPGYDGVRREYLRTLYGNVFL
PICIRTLKTILKREKNAVMRKVPPRLRLIKKEVSGEFQVSCLAFGFYPRHINLTLLRDGQ
PVAEQELTGGEVLPSGDGTYQLRKSLEVSTEELKKRHNYTCTASHLSLDNKLDVSWESGA
ERVHLSTLSVLLVMLLVVILLGIFICVKGRRRTASQT
>Onts-LNA2 XP_024264663.1 NW_020130735:91.015-93.632
MGKLSVFLFVLSFSTIVNAGSGSHSLWALANYIIGETPFPEFTVVVMLDDIHVAYYDSND
KQPVYRGPKPTGKIHDELFQDGDYVFGIMYHHMKGRSFHLKHHFNLTGGVQVQQRMSGCE
MLDNGEPALIMEKDTFNAIYTDQTLYYNMTHFTYDAGKLRPGYDGVRREYLRTLYGNVFL
PICIRTLKTILKREKNAVMRKVPPRLRLIKKEVSGEFQVSCLAFGFYPRHINLTLLRDGQ
PVAEQELTGGEVLPSGDGTYQLRKSLEVSTEELKKRHNYTCTASHLSLDNKLDVSWESGA
ERVHLSTLSVLLVMLLVVILLGIFICVKGRRRTASQT
>Onts-LKA1ps No prediction NW_020130477:58.887-59.660 pseudogene
FPEFTVVVMLDDVQVTYYDSKRDHFTKAHFNLTGGVQVQQRMSGCEMLDNGEQACSW*RT
LPCHYTDQMVFTT*NIFLYAGNYYEDGMDEASI*KVVLFRMFTVPPCLRTQKTLLSREEE
RCDA*VPPRLRLIKKEVSGGFQVSCLAFGFYPRQGEVLPSGDGTYQLRKSLEVSTEELKK
RHNYTCTASHLSLDNKLDVSWESGT
>Onts-LFA1ps No prediction NW_020130477:66.136-66.960 pseudogene
SPGSGSHSLWALATI*AERPFP*VYGCGDVGCVQVAYYDSNMKHFIYRGHNTSDKIHDDL
AQDGAYVFGIIYQS*KRDPST*STT*ISRKELSSAKMSDVSVGHGEPALSCLRGLLMAFL
*IMRYLQHDTFYNTILGHYL*VLWVRQAYEKALFENVLLPICIKSLKRFLTREKNIVMRK
VPPRLRLIKKEVSGGFQVSCLAFGFYPRHINLTLLRDGQPVAEQELTGGEVLPSGDGTYQ
LRKSLEVSTGELKKRHNYTCTAFHLSLDNKLDVSW
>Onts-LGA2.1ps No prediction NW_020130477:80.914-80.085 pseudogene
GSGSHSLWALATYITGETPFPEFTVVVMLDDVQVAYYDSNDKQSVYRIYQSMKDRSVELK
HHFNLTEGVQVQQKLTGCEMLDNGEPVLVMFKGVFNAIVYLPICRKSLKRFLKREKNVVM
RKVPPRLRLI*KEVSGGFQVSCLVFGFYPRHINMTLLRDGQPVAEQELTGGEVLPSGDGT
YQLRKSLEVSTEELKKRHNYTCTASHLSLDNKLDVSW
>Onts-LCA1 No prediction NW_020130477:83.100-83.996
YKMFITHFTLTALPGSGSHSLWALATYISGETPFPEFTVVVMLDDVQVTYLDSNMKHFIY
RGHNTSDKINDDEDKNGXFVFXIMYNHMKERYFHLKHHLNLTEGVQVQQRMAGCEMFDNG
EPALIMTKNTFNAVFVDHAIYYNLTHFTYDAGQLLQGWDGIRQAQEKILYENVLLPICIK
TLKTILKRERNVVMRKVPPRLRLIKKEVSGGFQVSCLAFGFYPRHINMTLLRDGQPVAEQ
ELTGGEVLPSGDGTYQLRKSLEVSTEELKKRHNYTCTASHLSLDNKLDVSWESGAEMSS
>Onts-LCA2 No prediction NW_020130477:163.908-164.804
YKMFITHFTLTALPGSGSHSLWALATYISGETPFPEFTVVVMLDDVQVTYLDSNMKHFIY
RGHNTSDKINDDEDKNGXFVFXIMYNHMKERYFHLKHHLNLTEGVQVQQRMAGCEMFDNG
EPALIMTKNTFNAVFVDHAIYYNLTHFTYDAGQLLQGWDGIRQAQEKILYENVLLPICI
KTLKTILKRERNVVMRKVPPRLRLIKKEVSGGFQVSCLAFGFYPRHINMTLLRDGQPVAE
QELTGGEVLPSGDGTYQLRKSLEVSTEELKKRHNYTCTASHLSLDNKLDVSWESGAEMSS
>Onts-LGA2.2ps No prediction NW_020130477:166.990-167.819 pseudogene
GSGSHSLWALATYITGETPFPEFTVVVMLDDVQVAYYDSNDKQSVYRIYQSMKDRSVELK
HHFNLTEGVQVQQKLTGCEMLDNGEPVLVMFKGVFNAIVYLPICRKSLKRFLKREKNVVM
RKVPPRLRLI*KEVSGGFQVSCLVFGFYPRHINMTLLRDGQPVAEQELTGGEVLPSGDGT
YQLRKSLEVSTEELKKRHNYTCTASHLSLDNKLDVSW
>Onts-LFA2ps No prediction NW_020130477:180.944-181.768 pseudogene
SPGSGSHSLWALATI*AERPFP*VYGCGDVGCVQVAYYDSNMKHFIYRGHNTSDKIHDDL
AQDGAYVFGIIYQS*KRDPST*STT*ISRKELSSAKMSDVSVGHGEPALSCLRGLLMAFL
*IMRYLQHDTFYNTILGHYL*VLWVRQAYEKALFENVLLPICIKSLKRFLTREKNIVMRK
VPPRLRLIKKEVSGGFQVSCLAFGFYPRHINLTLLRDGQPVAEQELTGGEVLPSGDGTYQ
LRKSLEVSTGELKKRHNYTCTAFHLSLDNKLDVSW
>Onts-LKA2ps No prediction NW_020130477:188.244-189.017 pseudogene
FPEFTVVVMLDDVQVTYYDSKRDHFTKAHFNLTGGVQVQQRMSGCEMLDNGEQACSW*RT
LPCHYTDQMVFTT*NIFLYAGNYYEDGMDEASI*KVVLFRMFTVPPCLRTQKTLLSREEE
RCDA*VPPRLRLIKKEVSGGFQVSCLAFGFYPRQGEVLPSGDGTYQLRKSLEVSTEELKK
RHNYTCTASHLSLDNKLDVSWESGT

>Onts-LEA XP_024264766.1 NW_020130833:12.771-13.858
MGKLSVLLFLHSFSTIVNAGSGSHSLWALATYIIGETPFPEFTVVVMLDDVQVTYYDSNM
KHFIYRVNNTPNKMHDNEAQDGAYILGMMYNNMKERSFHLKHHFNLTGGVQVQQRMSGCE
MLDNGEPAMFMVKDTFNAIYTDQMVYYNMTHFXYDAGKLLRGWDGMRQAFERVLFENVHL
PICIRTQKTLLSREKNVVMRKVPPRLRLIKKEVSGGFQVSCLAFGFYPRHINLTLLRDSQ
PVAEQELTGGEVLPSGDGTYQLRKSLEVSTEELKKRHNYTCTASHLSLDNKLDVSWESGT
ERVHLSTL
>Onts-LFA3 XP_024264767.1 NW_020130833:17.067-17.597
MGKLSVLFFISFYTIGNAGSGSHSLWALATYISGETPFPEFTVVVMLDDVQVAYYDSNMK
HFIYRGHNTSDKIHDDLAQDGAYVFGIIYQSMKERSFHLKHHLNLTEGVQVQQRMSGCEM
LDNGEPALIMFKETFNGIFVDHAIFNSMTHFTYDSGTLLVGYYGIRQAYEKALFENVLLP
ICIKSLKRFLKREKNIVMRKVPPRLRLIKKEVSGGFQVSCLAFGFYPRHINLTLLRDGQP
VAEQELTGGEVLPSGDGTYQLRKSLEVSTEELKKRHNYTCTA
>Onmy-LHA1 XP_021463538.1 chr.6 NC_035082:75.466.317-75.468.798
MCKLSVFLIVLSFYTIVNAGSGSHSLWALATYINGETPFPEFTVVVMLDDVQVGYYDSNM
KECIYSGLNPTDKIHDDLAQDGAYVFGTMYQSIKERSFQLKHHLNLTEGVQVQQRMAGCE
MLDNGEPALIMTKDTFNAIHTDRTLYYNITHLTYDAGKLLPGWDAMRKEYLRTLFEKVFL
PICIKTMKTFLKREKNVVMRKVPPRLRLIKKEVSGGFQVSCLAFGFYPRHINLTLLRDGQ
PVAEQELTGGEVLPSGDGTYQLRKSLEVSTEELKKRHNYTCTASHLSLDNKLDVSWESGA
ERVHLFILSAPLVMALIVILFCILICLARRIRAASQNLQLASVDALEADERNLSSDSEI
>Onmy-LAA1 XP_021421958.1 chr.16 NC_035092:45.979.845-45.987.016
MNFYTLFLIYLPIVNAGKSSHSLWGLATHVLGETEFPEFCVLWMLDDVQVGYYDSNSWRF
ISRTDENIDEEYSKTVQGASWDVYLSMRKRSSLLQHRFNSTTGIFVHQRLIGCELEDNEK
QGQLMIKEAFNGIDGGVLNFNKLQYNYHPKWPELEFNQQRTQYIQMGLDKVYLPICIKSL
KDYLKKEEKLVMRKVRPRVRLISKESTDTEGAKITCLAFGFYPRHINLTLLRDGQPVAEH
ELKGGQLLPNGDWTYQLRKSLTITVQELRERPNYTCTANHISMDNKLDVSWVPDTGPDSA
SIIPVVLVMAVVLILIGILVVIGMWKWKHAGVPTFSGHIYSAAKDTETEKSNSSLETEST
DS
>Onmy-LAA2ps no prediction chr.9 NC_035085:48.584.161-48.585.737
FPEFCVVSILDNGQMGYYDSNAWICISKTDGISDEAFEGVAQNRFWDMFLSMRKQVSFLL
HHFNNGIFVNQRHCGCELLDNNEQGQIMIKESFNGFDGALNFNKLQHPKWPKLAFNQHRT
QYIQMGLGKAALEKLHLPICIKTLKDYLKKGEMLVMRRGAFGFYPCHINLTLLRNGQRVA
EHDLTGGQLLPNGDRL*QLRMSLTISAQELRERERHHYTCTATHLS
>Onmy-LDA XP_021425739.1 chr.17 NC_035093:67.362.765-67.364.428
MLHKATLQNHGQTVNLFVCSFILHHCQRRGYHITDKINDEAQDGSYVLGTMYHHMKERSF
RLKHHLNLTEGVHVQQRIGGCEILHNGEPALIMTKNSFNGIFADYAVYYNMTHFTYDSGQ
LLLGYNWMRQATERTLYANVWLPICINTLKKCLNREKNFVMRRVPPRLRLIKKEVSGDLQ
VICLAFGFYPRHINLTLLRDGHPVAEQELTGGEVLPSGDGTYQLRKSIYVSTEELREKHN
YTCTASHLSLNNKLDVSWESGAERVHLFILSAPLVMALIVILFCILICLARRIRAASQNL
QLASVAALEADERNLSSDSEI
>Onmy-LIA XP_021434561.1 chr.22 NC_035098:9.835.124-9.837.436
MVKLCFFWLFLSLYTIINAGSHSLLAFATCISGEAPFPECSVVLMQDDIQVGYFDSNTEQ
FIHKGPYAPDETEVDVAQDAANVFGHMFLSMKRRLSDLRFRFNSTGNIDVQQRMAGCEML
DTGEPGLLLSTDAFNAILADLIYYNMTHYSYNSGNLLSPWSEVHQTYVKWHYQTIYLPIC
IKTLKTFRESLKNFVMRKVRPRVRLIQKAMSGGARVSCLAYGFYPRHINLTLLRDGQPIL
EQDMTGGQLLPNGDGTYQLRKSLEVITEELRERHNYTCTTSHLSLDNKLDVSWIPESGID
RVGLYVKSGPLGMVAIIIILLSIFVCVRRRNAAGSQTLSQLSNANDAQVAEQMSLSSHSET
>Onmy-LHA2 XP_021442420.1 chr.26 NC_035102:33.688.970-33.692.422
MCKLSVFLIVLSFYTIVNADPGSHSLWALATYIIGETPFPEFTVVVMLDDVQVGYYDSNM
KHLIYSGLPTYKIRDDEAQDGAYVFGIIYQSIKERSFQLKHHLNLTRGVQVQQRIAGCEM
LNNGELMIMSKNTFNAVFVDHEIYYNMTHFTYNSGKLLPEWDAMRREYLKTLFGNVLLPI
CIRTLKTILKREKNIVMRKVPPRLRLIKKEVSGGLKVSCLAFGFYPRHINLTLLRDGQPV
AEQELTGGEVLPSGDGTYQLRKRLEVSTEELKKRHNYTCTASHLSLDNKLDVSWESGAER
VHLSTLSALLVMLLVVILLGIFICVKRRRRSASQALVTTCKR
>Onmy-LEA1pt XP_021442443.1 chr.26 NC_035102:33.977.597-33.980.079
MRKVPPRLRLIKKEVSGGLKVSCLAFGFYPRHINMTLLRDGQPVAEQDLTGGEVLPSGDG
TYQLRKSLEVSTEELKKRHNYTCTASHLSLDNKLDVSWESGAERVHLSTFSALLVMLLIV
ILLGIFICVRRWRYTASQSKIANVDAKVSEEMNLSSDSET
>Onmy-LGA XP_021442415.1 chr.26 NC_035102:33.996.968-34.014.691
MGKLSVFLFVLSFYTIVNAGSGSHSLWALATYISGETPFPEFTVVVMLDDVQVTYYDSND
KQSVYRGQHITDKTEDDDAQDEAYIFGDIYQSMKDRSVELKHHFNLTEVVQVQQRLTGCE
MLDNGEPALAMFKNIFNGISEDYALYYNSTHFTYDAGKLLLGWDGIMQTLMKTKFENFYL
PICIKNLKRFLKREKNIVMRKVPPRLRLIKKEVSGGFQVSCLAFGFYPRHINMTLLRDGQ

PVAEQDLTGGEVLPSGDGTYQLRKSLEVSTEELKKRHNYTCTAFHLSLDNKLDVSWEFGA
ERVHLFTLSALLVMLLIVILLGIFFCVKRRWSNTASQSKLANIDATVSEETNLSSDSET
>Onmy-LFA XP_021442419.1 chr.26 NC_035102:34.008.645-34.010.877
MGKLSVLFFISFFTIGNAGSGSHSLWALATYISGETPFPEFTVVVMLDDVQVGYYDSNMK
HFIYKGHNTSDKIHDDLAQDGGYVFGIIYQSMKERSFHLKHHLNLTEGVQVQQRMSGCEM
LDNGEPALIMFKETFNGIFVDHAIYNSMTHFIYDSGTLLLGYYGIRQAYEKALFENVLLP
ICIKSLKRFLKREKNVVMRKVPPRLRLIKKEVSGGFQVSCLAFGFYPRHINLTLLRDGQP
VAEQDLTGGEVLPGGDGTYQLRKSLEVSTEELKKRHNYTCTAFHLSLDNKLDVSWESGTE
RVHLSTLSVLLVMMLILILSIFICVKRRWRCTASHLKLVNIDAKA
>Onmy-LBA1 XP_021442411.1 chr.26 NC_035102:34.020.825-34.024.713
MRGYTIKIMGKLSVFLFVFSFYTIVNAGSGSHSLWALATYISGETPFPEFTVVVMLDDIQ
VTYYDSNMKHFIYKGHNTSDKIHDDEAKNGDFVFGVMYHHMKERYFHLKHHFNLTEGVQV
QQKLTGCEMFDNGEPALVMFKDAFNAIYTDRTLYYNMTHFTYDAGKLLLGWDGMRQAYER
TLYENVYLPICIKSLKKILKREKNIVMRKVPPRLRLIKKEVSGGFQVSCLAFGFYPRHIN
LTLLRDGQPVAEQDLTGGEVLPSGDRTYQLRKSLEVSTEELKKRHNYTCTASHLSLDNKL
DVSWESGAERVHLSTLSALLVMLLVVILLGIFICVKRRWSNTASQSKLANVDATVSEEMN
LSSDSET
>Onmy-LEA2 XP_021442414.1 chr.26 NC_035102:34.061.638-34.063.352
MGKLSVLLFVHSFSTIVNAGSGSHSLWVLATYISGETPFPEFTVVVMLDDIQVTYYDSNM
KHFIYRVNNTPNKMHDNEAQDGAYVFGMMFNNMKERSFNLKHHFNLTEGVQVQQRMSGCE
MLDNGEPVMFVVKDTFNAIYTDQMVCYNMTHFIYDAGKLLRGWDGMRQAFERVLFENVHL
PICIRTLKTILKREKNIVMRKFPPRLRLIKKEVSGGLKVSCLAFGFYPRHINLTLLRDGQ
PVAEQDLTGGEVLPSGDGTYQLRKSLEVSTEELKKRHNYTCTASHLSLDNKLDVSWESGA
ERVHLSTLSVLLVMLLVVILLGIFICVKRRWSNTASQSKLANVDATVSEEINLCSDSEN
>Onmy-LEA3 XP_021442418.1 chr.26 NC_035102:34.066.830-34.069.490
MGKLSVLLFVHSFSTIVNAGSGSHSLWVLATYISGETPFPEFTVVVMLDDIQVTYYDSNM
KHFIYRVNNTPNKMHDNEAQDGAYVFGMMFNNMKERSFNLKHHFNLTEGVQVQQRMSGCE
MLDNGEPVMFVVKDTFNAIYTDQMVCYNMTHFIYDAGKLLRGWDGMRQAFERVLFENVHL
PICIRTLKTILKREKTFAMRKVPPRLRLIKKEVSGGLKVSCLAFGFYPRHINLTLLRDGQ
PVAEQDLTGGEVLPSGDGTYQLRKSLEVSTEELKKRHNYTCTASHLSLDNKLDVSWESGA
ERVHLSTFSALLVMLLIVILLGIFICVRRWRYTASQSKIANVDAKVSEEINLSSDSET
>Onmy-LCA XP_021442413.1 chr.26 NC_035102:34.070.204-34.072.325
MGKLSVFLFVLSFCTIVNSGSGSHSLWALATYISGETPFPEFTVVVMLDDVQVTYYDSNM
KHFIYKGHNTSDKIHDDEAKNGDFVFGVMYHHMKERYFHLKHHLNLTEGVQVQQRMAGCE
MFDNGEPALIMTKNTFNAVFVDHAIYYNMTHFTYDAGKLLQGWDGMRQLQEKILYENVLL
TLCIRTLKTILKREKNVMMRKVPPRLRLIKKEVSGGFQVSCLAFGFYPRHINLTLLRDGQ
PVAEQELTGGEVLPSGDRTYQLRKRLEVSTEELKKRHNYTCTASHLSLDNKLDVSWESGA
E
>Onmy-LBA2pt XP_021442442.1 chr.26 NC_035102:34.084.331-34.089.088
MRKVPPRLRLIKKEVSGGLKVSCLAFGFYPRHINLTLLRDGQPVVEQELTGGEVLPSGDG
TYQLRKRLEVSTEELKKRHNYTCTASHLSLDNKLDVSWESGAERVHLSTLSALLVMLLVV
ILLGIFICVKRRWSNTASQSKLANVDATVSEEINLSSDSET
>Satr-LAA1ps no prediction chr.30 NC_042986:17.684.542-17.685.599
F*IITGIFVHQRLIGCELVDNVKQG*LTIKEVFNGIDGGILNFNELQYNYHPKWPELEFN
QQRTQYVQMGLDKVYLPICIKSLKDYLKKEEKLVMRKVSPRVRFISKESTDSEGAKITCL
AFPFYPRHINLTLLRDGQPVAEHQLKGGQLLPN*DWTYQLRNSLPITVQEL*ERRERHNY
TCTASHIRMHNTLDVSWPDTSPDSASIIPVVLVMAVVFILIGSLVVFGMWKWKHTG
>Satr-LAA2pt no prediction chr.28 NC_042984:22.516.693-22.518.122
SNAWICVSKTDGNSDEAFETVAQNTFCGVFLSMRKQLSFLQHRFNKDTGIFVNQRRGCEL
RDNDKQGQIMIKESFNGIDGDLNLNKLQYHPKWPELVFNQHRTQYIQMGLEKVRLPICIK
TLKDYLKKEEMLVMRRGLWTYNCLLKNIRNTTGANLQAFGFYPCHINLTLLRNGQPVAEH
DLIGGQLLPNGDRLYQLRMSLTISAQELRERERDIITPLLPLICLDNKLDVSW
>Satr-LNAps No prediction chr.12 NC_042968.1:35.027.883-35.028.903
SGLGSHSL*ALATYIIGETPFPEFTVVVMLDDVQVGYYDSKDKLSVYRGFNPTNKIYDDL
AQEGHSVFQTMYHGMKERAFQLKHHYNLTGGVQVQQRMAGCEMLDNGEPAMIMYKETFNA
IYTDQTVISNTTHFTYDSGKLLLGWDGMMQDFKRTLSINVHLPRCIRTQKTLLKREKNIV
MHKVPPRLRLIKKEVSGGFQVSCLAFGFYPRHINLTLLRDGQPVAEQELTGGEVLPSGDG
TFQLRKSLEVSTEELKKRHNYTCTASHLSLDNKLDVSWESGAERVHLSTLSVLLMMLLIL
ILLVIFICVKRRRRTASKALSQLANVDAKVAEEMNLSSDSAT
>Satr-LGA2.3 No prediction chr.12 NC_042968.1:35.639.205-35.640.653
MGKLSVFLFVLSFYPIVNAGKGGSGSHSLWALATYISGETPFPEFTVVLMLDDVQVTYYD
SNDKQYVYRGQNIKEKTEDDEAKDEAHIYGDIYHSMKDRSFELKHHFNLTEGVQVQQRWT
GCEMMDNGEPGLAMFKNIFNGISEDYAMYYNTTHVTYNAGKILLGWDWKMQAYNINKFEN

```
YYLPICIKHLKTLLKREKNIVMRKVPPRLRLIKKEVSGGFQVSCLAFGFYPRHINLTLLR
DGQPVAEQELTGGEVLPSGDGTYQLRKSLEVSTEELKKRHNYTCTASHLSLDNKLDVSWE
SGAERVHLSTLSVLLMMLLILILLVIFICVKRRWSNTASQMKLANFDAKVSEEMNLSSDS
ET
>Satr-LMA2 No prediction chr.12 NC_042968.1:35.651.573-35.652.582
GSGSHSLWALATYIRGETPFPEITVVVMLDDVQVAYYDSNMKHFIYRGHNTSDKIHDEAQ
EGAYLLGKMYHHMKERSFNLKHHFNLTEGVQAQQRMSGCEMLDNGEPVLIMVKDTFNAIY
TDQMVYYNMTHFIYDAGKLLLGWDGMRQAYERVLFENVHLPICIRTQKTLLKREKNVVMR
KVPPRLRLIKKEVSGGFQVSCLAFGFYPRHINLTLLRDGQPVAEQELTGGEVLPSGDGTY
QLRKSLEVSTEELKKRHNYTCTASHLSLDNKLDVSWESGAERVHVKRRRSNTASQSELAN
VDAKVSEEMNLSSVSET
>Satr-LGA2.4 XP_029623984.1 chr.12 NC_042968.1:35.665.172-35.667.127
MTRGYTITIMGKLSVFLFVLSFYPIVNAGSGSHSLWALATYISGETPFPEFTVVLMLDDV
QVTYYDSNDKQYVYRGQNIKEKTEDDEAKDEAHIYGDIYHSMKDRSFELKHHFNLTEGVQ
VQQRWTGCEMMDNGEPGLAMFKNIFNGISEDYAMYYNTTHVTYNAGKILLGWDWKMQAYN
INKFENYYLPICIKHLKTLLKREKNIVMRKVPPRLRLIKKEVSGGFQVSCLAFGFYPRHI
NLTLLRDGQPVAEQELTGGEVLPSGDGTYQLRKSLEVSTEELKKRHNYTCTASHLSLDNK
LDVSWESGAERVHLSTLSVLLMMLLILILLVIFICVKRRWSNTASQMKLANFDAKVSEEM
NLSSDSET
>Satr-LMA3 No prediction chr.12 NC_042968.1:35.686.748-35.687.758
GSGSHSLWALATYIRGETPFPEFTVVVMLDDVQVAYYDSNMKHFIYRGHNTSDKIHDEAQ
EGAYLLGKMYHHMKERSFNLKHHFNLTEGVQAQQRMSGCEMLDNGEPVLIMVKDTFNAIY
TDQMVYYNMTHFIYDAGKLLLGWDGMRQAYERVLFENVHLPICIRTQKTLLKREKNVVMR
KVPPRLRLIKKEVSGGFQVSCLAFGFYPRHINLTLLRDGQPVAEQELTGGEVLPSGDGTY
QLRKSLEVSTEELKKRHNYTCTASHLSLDNKLDVSWESGAERVHVKRRRSNTASQSELAN
VDAKVSEEMNLSSVSET
>Satr-LGA2.5 XP_029623985.1 chr.12 NC_042968.1:35.700.358-35.702.313
MTRGYTITIMGKLSVFLFVLSFYPIVNAGSGSHSLWALATYISGETPFPEFTVVLMLDDV
QVTYYDSNDKQYVYRGQNIKEKTEDDEAKDEAHIYGDIYHSMKDRSFELKHHFNLTEGVQ
VQQRWTGCEMMDNGEPGLAMFKNIFNGISEDYAMYYNTTHVTYNAGKILLGWDWKMQAYN
INKFENYYLPICIKHLKTLLKREKNIVMRKVPPRLRLIKKEVSGGFQVSCLAFGFYPRHI
NLTLLRDGQPVAEQELTGGEVLPSGDGTYQLRKSLEVSTEELKKRHNYTCTASHLSLDNK
LDVSWESGAERVHLSTLSVLLMMLLILILLVIFICVKRRWSNTASQMKLANFDAKVSEEM
NLSSDSET
>Satr-LMA4 No prediction chr.12 NC_042968.1:35.712.951-35.713.960
GSGSHSLWALATYIRGETPFPEFTVVVMLDDVQVAYYDSNMKHFIYRGHNTSDKIHDEAQ
EGAYLLGKMYHHMKERSFNLKHHFNLTEGVQAQQRMSGCEMLDNGEPVLIMVKDTFNAIY
TDQMVYYNMTHFIYDAGKLLLGWDGMRQAYERVLFENVHLPICIRTQKTLLKREKNVVMR
KVPPRLRLIKKEVSGGFQVSCLAFGFYPRHINLTLLRDGQPVAEQELTGGEVLPSGDGTY
QLRKSLEVSTEELKKRHNYTCTASHLSLDNKLDVSWESGAERVHVKRRRSNTASQSELAN
VDAKVSEEMNLSSVSET
>Satr-LGA2.6 XP_029623983.1 chr.12 NC_042968.1:35.729.951-35.731.907
MTRGYTITIMGKLSVFLFVLSFYPIVNAGSGSHSLWALATYISGETPFPEFTVVLMLDDV
QVTYYDSNDKQYVYRGQNIKEKTEDDEAKDEAHIYGDIYHSMKDRSFELKHHFNLTEGVQ
VQQRWTGCEMMDNGEPGLAMFKNIFNGISEDYAMYYNTTHVTYNAGKILLGWDWKMQAYN
INKFENYYLPICIKHLKTLLKREKNIVMRKVPPRLRLIKKEVSGGFQVSCLAFGFYPRHI
NLTLLRDGQPVAEQELTGGEVLPSGDGTYQLRKSLEVSTEELKKRHNYTCTASHLSLDNK
LDVSWESGAERVHLSTLSVLLMMLLILILLVIFICVKRRWSNTASQMKLANFDAKVSEEM
NLSSVSET
>Satr-LMA5 No prediction chr.12 NC_042968.1:35.742.639-35.743.650
GSGSHSLWALATYIRGETPFPEFTVVVMLDDVQVAYYDSNMKHFIYRGHNTSDKIHDEAQ
EGAYILGKMYHHMKERSFNLKHHFNLTEGVQAQQRMSGCEMLDNGEPVLIMVKDTFNAIY
TDQMVYYNMTHFIYDAGKLLLGWDGMRQAYERVLFENVHLPICIRTQKTLLKREKNVVMR
KVPPRLRLIKKEVSGGFQVSCLAFGFYPRHINLTLLRDGQPVAEQELTGGEVLPSGDGTY
QLRKSLEVSTEELKKRHNYTCTASHLSLDNKLDVSWESGAERVHLSTLSVLLMMLLIVIL
LVIFICVKRRWSNTASQSELANVDAKVSEEMNLSSVSET
>Satr-LMA6 No prediction chr.12 NC_042968.1:35.745.282-35.746.293
GSGSHSLWALATYIRGETPFPEFTVVVMLDDVQVAYYDSNMKHFIYRGHNTSDKIHDEAQ
EGAYILGKMYHHMKERSFNLKHHFNLTEGVQAQQRMSGCEMLDNGEPVLIMVKDTFNAIY
TDQMVYYNMTHFIYDAGKLLLGWDGMRQAYERVLFENVHLPICIRTQKTLLKREKNVVMR
KVPPRLRLIKKEVSGGFQVSCLAFGFYPRHINLTLLRDGQPVAEQELTGGEVLPSGDGTY
QLRKSLEVSTEELKKRHNYTCTASHLSLDNKLDVSWESGAERVHLSTLSVLLMMLLIVIL
LVIFICVKRRWSNTASQSELANVDAKVSEEMNLSSVSET
```

>Satr-LCA2 XP_029622515.1 chr.12 NC_042968.1:35.752.335-35.758.141
MCTKTCYTRKHFLNHGQAVCILFVLSFYTIVNSGSGSHSLWALATYISGATPFPEFTVVV
MLDDVQVTYYDSNMKNFIYSGHNTSDKIHDDEAKNGDFVFGVMYHHMKERYFHLKHHLNL
TEGVQVQQRMAGCEMFDNGEPALIMTKNIFNAVFVDRAIYYNITHFTYDAGKLLQGWDGM
RQAQEKILYENVLLTICIRTLKTLLKREKNIVMRKVPPRLRLIKKEVSGGFQVSCLAFGF
YPRHINLTLLRDGQPVAEQELTGGEVLPSGDGTYQLRKSLEVSTDELKKRHNYTCTASHL
SLDNKLDVSWESGAERVHLSTLSVLLMMLLILILLVTFICVKRRWSNTASQSELANVDAK
VSEEMNLSSVSET
>Satr-LCA1 XP_029623986.1 chr.12 NC_042968.1:35.796.559-35.799.118
MGKLSIFLLVLSFYTIVNAGSGSHSLWALATYIRGETPFPEFTVVVMLDDVQVTYYDSNM
KHFIYRGHNTSDKIHDDEAKNGDFVFGVMYHHMKERYFHLKHHLNLTEGVQVQQRMAGCE
MLDNGEPALIMTKNNFNAVFADHAIYYNITHFTYDAGKLLQGWDGMRQAQEKILYENVLL
TLCIRTLKTLLKREKNIVMRKVPPRLRLIKKEVSGGFQVSCLAFGFYPRHINLTLLRDGQ
PVAEQELTGGEVLPSGDGTYQLRKSLEVSTDELKKRHNYTCTASHLSLDNKLDVSWESGA
ERVHLSTLSVLLMMLLILILLVTFICVKRRWSNTASQSELANVDAKVSEEMNLSSVSET
>Satr-LDA XP_029630277.1 chr.14 NC_042970.1:10.704.457-10.706.122
MGKLSIFLFVLSFYTIVNAGSGSHSLWALATYIIGETSFPEFTVVLMLDDIQIGYYDSNI
KQSVYRGYHITDKMNDEAQDGTYVLGTMYDHMKERSFRLKHHLNLTEGVHVQQRIGGCEI
LHNGEPALIMTKNSFNAIFEDYAVYYNMTHFTYDSGKLLLGYNWLRQATERTLYTNVWLP
ICINTLKKCLKRENFVMRRVPPRLRLIKKAVSGDIQVICLAFGFYPRHINLTLLRDGHPV
AEQELTGGEVLPSGDGTYQLRKSIYVSTEELRERHNYTCTASHLSLNNKLDVSWESGAER
VHLFILSAPLVMALIVILFCIFICLVRRIRAASQNLLQLASVDALEADAMNLSSDSEKT
>Satr-LIA XP_029558880.1 chr.20 NC_042976:33.824.877-33.826.457
MAKLCFFLILLSLYTIVNPGSHSLWAFATCISGEAPFPECSVVLMQDDIQVGYFDSNKEQ
FIHKGPYAPDETEVEEAQDAAYVFGHMFLSMKRRLSDLRYRFNSTGNIDVQQRMAGCEML
DTGEPGLILSTDAFNAILADLIYYNMTHYSYNSGNLLSPWSEVHQTYAKWRYQTIYLPVC
IKTLKRFLERLKNFVMRKVRPRVRLIQKAMSGGARVSCLAFGFYPRHINLTLLRDGQPIV
EQEMAGGQLLPNGDGTYQLRKSLEVNTEELRERHNYTCSTSHLSLDNKLDVSWIPESGMD
RVGLYVKSAPLATVAIIILLSIFVCVRRRNTAGSQTLSQLSNANDAQVAEQISLSSHSET
>Satr-LJAps No prediction chr.24 NC_042980:29.522.035-29.522.883
SGSGSHSLWAFATYIIGDTPFPEYSVLLLLDDIEVGYYERQFVYRGHNALDEKEMGIVLD
IASVFGAMSFSMKGRSYDLKQLFYFTEGIHVQQRMACCEMLDNDKLVLILSRSTFNKIVA
DGMCYNMTQNTYYTGNPQLAWDEVKLEYVKMLYAHVYLPLCIKTLNIFLERKKNIVMRKV
RPRIRLIKKAKSGGLQVSCLAFGFNPRHTNLTLLRDSQPIAEQELTGGQLPPNGDWVY*L
RKSLEVITEELRERHNYTCTVSHLSLDNKLDVSWVPEPGRDRV
>Satr-LLAps No prediction chr.24 NC_042980:40.874.193-40.876.693
SGSGSHSLWAFATYISG*TPFPVLTVVLMLDDFQVGYFDSNIKRYIHKGYNASEETEVEE
AYHIAFIFGAMSFSLRAR*SELKRFNSKEGIHVQQRLAGCEMSDNGEPALIVSRESLNGI
YADLAIYYNMTHYSYNSGKLLYVKGLYQTIYLNICMDTLKTFLEKEKKCIMRKVRPRVRL
IQKDLSGGVQQMSCLPFGFYPRHINLTLLRDGRPIAEQVLNGGQLLHNGDGTYQLRKSLV
VSTQELREERHNYTCTASHLSLDNKLDVSWVPESGADRV
>Satr-LGA1.2 XP_029576627.1 chr.29 NC_042985.1:9.458.865-9.462.166
MRGYTIKIMGKLSVFLFVFSFYTIVSPGSGSHSLWALATYIVGETPFPEFTVVVMLDDVQ
VAYYDSNDKQSVYRGQHITKTKDDEAQDGAHVFGVIYQSMKDRSFELKHHFNLTEGVQVQ
QKITGCEMLNNGESALIMFKDVFNAIYTDRTLYYNMTHFTYDAGKLLLGWDGIRQAYERT
LYENVYLPICIKSLKRLLKREKNIVMRKVPPRLRFIKKEVSGGFQVSCLAFGFYPRHINL
TLLRDGQPVAEQELTGGEVLPSGDGTYQLRKSLEVSTEELKKRHNYTCTASHLSLDNKLD
VSWESGAERVHLSTLSVLLMMLLILILLGIFICVKRRWSNTASQSKLANVDAKVSEEINL
SSDSET
>Satr-LFA XP_029576634.1 chr.29 NC_042985.1:9.468.567-9.471.475
MGKLSVLFFILFYTIGNAGSGSHSLWALATYINGETPFPEFTVVVMLDDVQVGYYDSNMK
DFIYRGHNPTDKIHDDVAQDGAYVFGIIYQSIKERSFHLKHQLNLTEGVQVQQRMSGCEM
LDNGEPALIMFKETFNGIFVDHAIYNSMTHFTYDSGTLLLGYYGIRQAYEKALFENVLLP
ICIKNLKTILKREKNVVMRKVPPRLRFIKKEVSGGFQVSCLAFGFYPRHNNLTLLRDGQP
VAEQELTGGEVLPSGDGTYQLRKSLEVSTDELKKRQNYTCTASHLSLDNKLDVSWESGAE
RVHLSTLSVLLMMLLIVMLFGIFICVKRRWRCTASHLKLVNVDAKA
>Satr-LGA1.1pt XP_029576636.1 chr.29 NC_042985.1:9.493.218.-9.494.900
VRPTCAREVKERSFQLKHQFILTEGVQAQQKITGCEMLNNGEPALIMFKDVFNAIYTDRT
LYYNMTHFTYDAGKLLLGWDGIRQAYERTLYENVYLPICIKSLKRLLKREKNVVMRKVPP
RLRLIKKEVSGGFQVSCLAFGFYPRHINLTLLRDGQPVAEQELTGGEVLPSGDGTYQLRK
SLEVSTEELKKRHNYTCTASHLSLDNKLDVSWESGAERVHLSTLSVLLMMLLILILLGIF
IFVKRRWSNTASQSKLANVDANVSEEMYLYSVSET
>Satr-LHA XP_029576650.1 chr.29 NC_042985.1:10.157.491-10.162.043

MDKLSVFLFVLSFYTVVNTGSGSHSLWAFATYIIGETPFPEFTVVVMLDDVQVGYYDSNM
KDFIYSGLTTYKIHDNVAQDGAYVFGIIYQTIKEGSFQLKHHFNLTRGVQVQQRMAGCEM
LDNDEPALIMSKNTFNAVFADHEIYYNITHFTYNSGKLLPEWDAMRREYLRTLFGNVLLP
ICIKNLKTFLKIEKNVVMRKVPPRLRLIKKEVSGGFQVSCLAFGFYPRHINLTLLRDGQP
VAEQELTGGEVLPSGDGTYQLRKSLEVSTEELKKRHNYTCTASHLSLDNKLDVSWESGAE
RVHLSTLSVLLMMLLILILLGLFICIKRRRRTASQALSQLANVDAKVAEEINLSSDSAT
>Satr-LGA2.1ps No prediction NW_021823199:7.823-8.843 pseudogene
GSGSHSLWALATYISGETPFPPFPEFTVVLMLDDVQVTYYELQR*TVCLQGPKYQRENRG
**SKG*SSYNMEISTTA*RPIIELKHHFNLTEGVQVQQRWTGCEMMDNGEPGLAMFKNIF
NGISEEYAMYYNTTHVTYNAGKILLGWDWKMQAYNINKFENYYLPICIKHLKTLLKREKN
IVMRKVPPRLRLIKKEVSGGFQVSCLAFGSTPATSNLTLLRDGQPVAEQELTGGEVLPSG
DGTYQLRKSLEVSTEELKKRHNYTCTASHLSLDNKLDVSWESGAERVHLSTLSVLLMMLL
ILILLVIFICVKRRWSNTASQMKLANFDAKVSEEMNLSSDSET
>Satr-LMA1 No prediction NW_021823199:19.749-20.758
GSGSHSLWALATYIRGETPFPEFTVVVMLDDVQVAYYDSNMKHFIYRGHNTSDKIHDEAQ
EGAYLLGKMYHHMKERSFNLKHHFNLTEGVQAQQRMSGCEMLDNGEPVLIMVKDTFNAIY
TDQMVYYNMTHFIYDAGKLLLGWDGMRQAYERVLFENVHLPICIRTQKTLLKREKNVVMR
KVPPRLRLIKKEVSGGFQVSCLAFGFYPRHINLTLLRDGQPVAEQELTGGEVLPSGDGTY
QLRKSLEVSTEELKKRHNYTCTASHLSLDNKLDVSWESGAERVHVKRRRSNTASQSELAN
VDAKVSEEMNLSSVSET
>Satr-LGA2.2ps No prediction NW_021823199:33.983-35.055
SIFPYYLKKCVYIHSSPRFRITFSVGTCNIYKRRETPFPEFTVVLMLDDVQVTYYDSNDK
QYVYRGQNIKEKTEDDEAKDEAHIYGDIYHSMKDRSFELKHHFNLTEGVQVQQRWTGCEM
MDNGEPGLAMFKNIFNGISEDMYYNTTHVTYNAGKILLGWDWKMQAYNINKFENYYLPIC
IKHLKTLLKREKNIVMRKVPPRLRLIKKEVSGGFQVSCLAFGFYPRHINLTLLRDGQPVA
EQELTGGEVLPSGDGTYQLRKSLEVSTEELKKRHNYTCTASHLSLDNKLDVSWESGAERV
HLSTLSVLLMMLLILILLVIFICVKRRW**HCLPDETCQL*CKVSEEMNLSSVSET
>Sasa-LAA1ps No prediction chr.13 NC_027312:22.597.045-22.598.101
F*IITGICVNQRLTGCELVDNVKQG*LMIKEVFNGIDGGVLNFNELQYNYHPKWPELEFN
QQRTQYVQMGLDKVYLPICIKSLKDYLKKEEKLVMRKGRVSPRVRFISKESTDSEGAKIT
CLAFPFYPRHINLTLLRDGQPVAEHELKGGQFLPK*DWTYQLRKSLPITVQDLRERHNYT
CTASRISMHNKLDVSWPDTSPDSASIIPVVLVMAVVIILIGSLVVFGMWKWKHAG
>Sasa-LAA2pt no prediction chr.15 NC_027314:79.225.661-79.226.008
TGIFVNQRRGCELRDNDKQGQIMIKESFNGIDGALNFNKLQYHPKWPELVFNQHRTQYIQ
MGLGK
>Sasa-LCA XP_013983104.1 chr.11 NC_027310:38.173.444-38.175.944
MGKLSVFLFVLSFYTIVNSGSGSHSLWALATYISGETPFPEFTVVVMLDDVQVAYYDSNM
KHFIYRGHNTPNKIHDDEAKNGDFVFGVMYHHMKERYFHLKHHLNLTEGVQVQQRMAGCE
MFDNGEPALIMTKNTFNAVFADHAIYYNITHFTYDAGKLLQGWDGMRQAQEKILYENVLL
TLCIRTLKTILKREKNIVMRKVPPRLRLIKKEVSGGFQVSCLVFGFYPRHINLTLLRDGQ
PVAEQELTGGEVLPSGDGTYQLRKSLEVSTEELKKRHNYTCTASHLSLDNKLDVSWESGA
ERVHLSTLSVLLVMLLILILLVTFICVKRRWSNTASQSELANVDAKVSEEMNLSSDSEN
>Sasa-LCApt No prediction chr.11 NC_027310:38.129.721-38.130.194
MYYNISHYTYDAGKLLLGRDGMMQAYKITLLETFYLPLCIKSLKTFLKREKNVVMRKVPP
SVTLVKKEVYRRFQVSCLAFGFYPRHINLTLLRDGQPVAEQELTGGEVLPSGDGTYQLRK
SLEVSTEELKKRHNYTCTASHLSLDNKLDVSW
>Sasa-LDA XP_013989407.1 chr.12 NC_027311:80.563.693-80.565.337
MGKLSIFLFVLSFYTIVNAGSGSHSLWALATYIIGETPFPEFTVVLMLDDVQIGYYDSNI
KQSVYRGYHITDKMNDEAQDGTYVLGTMYDHMKERSFRLKHHLNLTEGVHVQQRIGGCEI
LHNGEPALIMTKNSFNAIFEDYAVYYNMTHFTYDSGKLLLGYNWIRQATERTLYANVWLP
ICINTLKKCLKRENFVMRRVPPRLRLIKKAVSGDIQVICLAFGFYPRHINLTLLRDGHPV
AEQELTGGEVLPSGDGTYQLRKSIYVSTEELRERHNYTCTASHLSLNNKLDVSWESGAER
VHLFILSAPLVMALIVILFCIFICLVRRIRAASQNLLQLASVDALEADEMNLSSDSEKT
>Sasa-LFA XP_014031975.1 chr.26 NC_027325.1:38.686.632-38.689.189
MGKLSVLFFILFYTIGNAGSGSHSLWALATYINGETPFPEFTVVVMLDDVQVGYYDSNMK
DFIYRGHNPTDKIHDDVAQDGAYVFGIIYQSIKERSFHLKHQLNLTEGVQVQQRMSGCEM
LDNGEPALIMFKETFNGIFVDHAIYNSMTHFTYDSGTLLLGYYGIRQAYEKALFENVLLP
ICIKNLKTILKREKNVVMRKVPPRLRLIKKEVSGGLQVSCLAFGFYPRHINLTLLRDGQP
VAEQELTGGEVLPSGDGTYQLRKSLEVSTEELKKRHNYTCTASHLSLDNKLDVSWESEAE
RVHLSTLSVLLVMLILILLGIFICVKRRWRCTASHLKLVNVDAK
>Sasa-LGA XP_014031973.1 chr.26 NC_027325.1:38.706.031-38.709.086
MGKLSVFLFVFSFYTIVSPGSGSHSLWALATYIVGETPFPEFTVVVMLDDVQVAYYDSND
KQSVYRGQHITKTKDDEAQDGAHVFRVIYQSMKDRSFELKHRFNLTEGVQVQQKITGCEM

LNNGESALVMYKDVFNAIYTDRTLYYNMTHFTYDAGKLLLGWDGIRQAYERTLYENVYLP
ICIKSLKRLLKREKNIVMRKVPPRLRLIKKEVSGGFQVSCLAFGFYPRHINLTLLRDGQP
VAEQELTGGEVLPSGDGTYQLRKSLEVSTEELKKRHNYTCTASHLSLDNKLDVSWESGAE
RVHLSTLSVLLMMLLILILLGIFICVKRRWSNTASQSELANVDAKVSEEINLSSDSET
>Sasa-LHA XP_014032010.1 chr.26 NC_027325.1:40.134.217-40.138.243
MGKLSVFLFVLSFYTIANAGSGSHSLWALATHIIGETPFPEFTVVVMLDDVQVGYYDSNM
KHFIYSGHNPTDKIHDDVAQDGAYVFGTMYQSIKERSFHLKYHLNLTRGVQVQQRMAGCE
MLNNGEPALIMSKNTFNAIYTDRTLYYNMTHFTYDAGKLLPGWDAMRREYLRILFGNVFL
PICIKTMKTFLKMEKNVVMRKVPPRLRLIKKEVSGGLQVSCLAFGFYPRHINLTLLRDGQ
PVAEQELTGGEVLPSGDGTYQLRKSLEVSTEELKKRHSYTCTASHLSLDNKLDVSWEPGA
ERVHLFTISILLMMLLIVILLGIFICVKRRRCTASQAFVTSCQHGGH
>Sasa-LIA XP_014019802.1 chr.21 NC_027320.1:21.859.914-21.861.394
MAKLCFFLILLSLYTIVNAGSHSLWAFATCISGEAPFPECSVVLMQDDIQVGYFDSNKEQ
FIHKGPYAPDETEVEEAQDAAYVFGHMFLSMKRRLSDLRYRFNSTGNIDVQQRMAGCEML
DTGEPGLILSTDAFNAILADLIYYNMTHYSYNSGNLLSPWSEVHQTYTKWHYQTIYLPVC
IKTLKRFLERLKNFVMRKVRPRVRLIQKAMSGGACVSCLAFGFYPRHINLTLLRDGQPIV
EQEMTGGQLLPNGDGTYQMRKSLEVNTEELRERHNYTCTTSHLSLDNKLDVSWIPESGMD
RVGLYVKSAPLATVAIIIILLSIFVCVRRRNTAGSQTLSQLSNANDAQVAEQISLSSHSET
>Sasa-LJAps No prediction chr.25 NC_027324:29.534.954-29.535.742
GSHSLWAFATYIIGDTPFPEYTVLLLLDDIEVGYYERQFVYRGHNALDEKEMGIVLDIAS
VFGAMSFSMKGRSYDLKQLFHFTEGIHVQQRMACCEMLDNDKLVLILSRSTFNKIVADGM
CYNMTQNTYYTGNPQLAWDEVKLEYVKMLYAHVYLPLCIKTLNIFLEREKNIVMRKVRPR
IRLIKKAKSGGLQVSCLVFGFNPRHTNLTLLRDSQPIAEQELTGGQLPPNGDWAY*LRKS
LEVITEELRERHNYTCTVSHLSL
>Sasa-LKApt No prediction chr.10 NC_027309:82.183.979-82.184.266
KVPPRLRLIKKEISGGFQVSCLVFGFYPRHINLTLLRDGQPVAEQELTGGEVLPSGDGTY
QLRKSLEVSTEELKKRHNYTCTASHLSLDNKLDVSW
>Sasa-LLAps No prediction chr.25 NC_027324:41.418.460-41.420.842
TVVLMLDDIQVGYFDSNIKRYIHKGYNASEETEVEEAYHIAFIFGPMSFSLRARSSELKR
FNS*EGIHVQQRLAGCEMSDNGEPALIVSSLNGIYADSAIYYNMTHYSYNSGKLFPPWSE
VHQTYVKGLYQTIYLNICMDTLKTFLEKEKKCIMHKVHARVRLIQKDMSGGVQQMSCLPF
GFYPRHINLTLLRDGRPIAEQVLNGGGQLLPNGDGTYQLRKSLVVSTQELRERHNYTCTA
SHLSLDNTLDVSW
>Sasa-LEA No prediction NW_012354073:3.249-4.233
MAKLSVLLFIHSFYTVVNAGSGSHSLWALATHIIGETPFPEFTVVVMLDDVQVAYYDSNM
KDFIYRVNNPSDKIHDEAQEGAYVFGVIYQSIKERSFQLKHHFILTEGVQAQQRMSGCEM
LDNGEPAMVMVKDTFNAIYTDQMVYYNMTHFIYDAGKLLPGWDGMRQAYERVLFENVHLP
ICIRTQKTLLKIEKNVVMRKVPPRLRLIKKEVSGGLQVSCPAFGFYPRHINLTLLRDGQP
VAEQELTGGEVLPSGDGTYQLRKSLEVSTEELKKRHNYTCTVSHLSLDNKLDVSW
>Eslu-LBA No prediction chr.02 NC_025969:9.994.527-9.995.536
GSGSHSLWALATYIIGETPFPEFIVVVMLDDVQVGYYDSKIKQPVYKGYHTSDKTKDDVA
QNRILYTAYENMYNNMKARSSELNQVFNFTERFQVQQSISGCEMLDNGDQALIMTKNNLN
SIYTDYAIYYNNTHFTYDAGNLLVGHDEKIQTYLFQNVWFPICIQTLKRCLKREKNVVMR
KVAPRLRLIKKRVSEGHRVSCLAFGFYPRHINLTLLRDGQPVAEQELTGGEVLPSGDGTY
QMRKSLEVSTKELMERHQYSCTASHLSLDNKLDVSWNSEVERVHLSILSAPLVMVSVVML
LGIYRCVKWR
>Eslu-LCA XP_010875198.1 chr.02 NC_025969.4:10.005.995-10.009.199
MGKLDVFLLQLFFCTIVNTGSGSHSLWAFATYIIGETSFPEYTIVEMLDDVQVIYYDSND
KHTVYRGHNTTHKINSNEIPDRTYLFEDIYHRMKERSYDYKRRFNHTEGIQVQQSMGGCE
MLDNGDPALIMMKDIFNAISVDHAIFYNITHYTYDPGKLLLGWDGIRQAVERSLVENVYL
PLCIKSLKAWLKREKNVVMRKVAPRLRLIKKDVSGGLRVTCLAFGFYPRHINLTLLRDGQ
PVAEQELTGGEVLPSGDGTYQMRKSLEVSTKELMERHQYSCTASHLSLDNKLDVSWKSGA
ERVHLSILSALLVIVLIVILLGISICLARRRHRLTDNFTTCKR
>Eslu-LPA XP_019909495.1 chr.15 NC_025982:6.802.982-6.806.755
LDIEKHSVTLLGSGSHSLWALATYIIGETPFPEFTVVLMLDDIQVGYYDSNNHQLLQKGH
EVPDKIDDDISQDSGRFVFGSMLNEMKARSSDLQRYFNSTDGIHVQQRMAGCEMKDNGKP
VLVMASDMYDGTVSDWLSFNMTHTSYYTGEPGLPWGEIQINFVKLHFQHVYLPICIRSLK
VRLEREKSTVMRKVRPRVRLIQKAMSGGVKVSCLAFGFYPRHINLTLLRDSQPIADQDLT
GGEVLPNGDKTYQLRKTLEVRTEELNEKHNYTCTASHLSLDNKLDVSWVPELEVRDGRFS
LLVPLVMMGICLLCVFICIKRKRSTTSSKTSSQLFNTNEVGPIS
>Eslu-LIA XP_010888243.1 chr.20 NC_025987:10.835.945-10.837.883
MRELRFFFLLLSTYSIVNAGSGSHSLWAFATCISGETPFAECSVVLMMDDIQVGYFDSNT
ERFVHKGHNTQNATDVDMAKDATYVFGHMFLSLKRRLSALKYRYNSTGDIVVNQRMTGCE

MLDNGEPALFLSTDAFNGIYADLIHYNITHYSYNSGNLLSPWSQVHQTNAKWRYQTIYLP
ICIKTLKKFLEKENNFVMRKVRPRVRLIQKAVSGGTRVSCLAFGFYPRHINLTLQRDGQT
IPDQELSAGLVLPSADGTYQLRKSLTVSAEELSEKHSYTCSTFHLSLDNKLDVSWVPRDG
TDPVLVSIPSAAVLIACLAILLACVWRRRNAAGTHVPHLSNADEAQAVEQISLSSHDS

AF4.4 Deduced S lineage amino acid sequences

>Saal-SAA XP_023991886.1 NW_019942612:111.299-114.376
MITTILISFMQFSIVAPHSLHRHCIATQGTLYPKNIQLVMIDDVIVYYYNSSAEQEAVVP
EWLNHPEGIEFWQEVHRNLKFNRYVMDTAVRVTSEHYNHSHDHFYQAHGRCGWKSDGTTE
AFMSHAYDGKDFVSFDVSTRTWTAAVSHAVFYKRKRETDLEDLVRLVIHYESGCIRWLKK
LLQFSVTFREPKVPAVSLFERSPHGNSEVEVTCHVTGFYPRAVQVEWLGAEGLPMVDGVS
SGEVLPNGDGSYQLRKSLTVPQEAQDTQSYSCLVLHSSKAENITVTWAPKKNLPNVLMAT
VIIVSVVLILTVLFTYLVWRRAVAQNPE
>Saal-SBAps LG22 No orf chr.22 NC_036862:35.259.299-35.262.616
HCIATQWILNPKLVHLVMIDDVIVY*YNSSAEQEAVMPEWLNHPEGIEFWRDVNRNLNCN
RYVMVTAVRVTSEHYNH*HSGQTWAAAVFQAVHFRKCRANGAFDLESLYYECIVRLKTIL
KYAAWIHDKKSPSSACY**KRPTDTLQGGGHVTGFYPQAVQVE*LGTQGLPLVDRVSSGE
VLPNGDGSYQLRKSSSVPQGSQDTQRYSCLLHSSVARNITVTW
>Onne-SAA XP_029537278.1 chr.14 NC_042548.1:49.264.637-49.267.655
MITTILISFMQFSIVAPHSLHRHCIATQGTLYPKNIQLVMIDDVIVYYYNSSAEQEAVVP
EWLNHPEGIEFWQEVHRNLKFNRYVMDTAVRVTSEHYNHSHDHFYQAHGRCGWKSDGTTE
AFMSHAYDGKDFVSFDVSTRTWTAAVSHAVFYKRKRETDVEDLVRLVIHYESGCIRWLEK
LLEFSVTVREPKVPAVSLFERPPHGNSEVEVTCHVTGFYPRAVQVEWLGAEGLPMVDRVS
SGEVLPNGDGSYQLRKSLTVPQEAQDTQSYSCLVLHSSIAGNITVTWAPKKNLANVLMAI
VIIVSVVLILTVLIKYLVWRRAVGKSQ
>Onne-SBAps chr.11 No prediction NC_042545:1.740.497-1.736.849
HCIATQWILNPKLVHSVMIDDVIVY*YNSSGKQEAVMPEWLNHPEGIEFWRDVNRNLNCN
QYVMDTAVRVTSEYYNY*HTADLHLGGYVTGFYPQAVQVEWLGAQGLPLVDRVSSGEVLP
NGDGPYQLRKSPSVPQGSQDTQRYSC
>Onki-SAA XP_020324114.1 chr.18 NC_034191:16.365.696-16.368.709
MITTILISFMQFSIVAPHSLHRHCIATQGTLYPKNIQLVMIDDVIVYYYNSSAEQEAIVP
EWLNHPEGIEFWQEVHRNLKFNRYVMDTAVRVTSEHYNHSHDHFYQAHGRCGWKSDGTTE
AFMSHAYDGKDFVSFDVSTRTWTAAVSHAVFYKRKRETDLEDLVRLVIHYESGCIRWLEK
LLEFSVTVREPKVPAVSLFERPPHGNSEVEVTCHVTGFYPRAVQVEWLGAEGLPMVDGVN
SGEVLPNGDGSYQLRKSLTVPQEAQDTQSYSCLVLHSSIAGNITVTWAPKKNLANVLMAI
VIIVSVVLILTVLFKYLVWRRAVAQNPE
>Onki-SBAps chr.15 NC_034188:77.214.861-77.218.450 pseudogene
HCIATQWILNPKLVHSVMIDDVIVYSYNSSGKQEAVMPEWLNHPEGIEFWRDVNRNLNCN
RYVMDTAVRVTSEHYNH*HLKRPTDDLHLGGYVTRFYPQAVQVEWLGAQGLPLVDRVSSG
EVLPNGDGSYQLRKSPSVPQGSQDTQRYSC
>Onts-SAA XP_024301302.1 chr.14 NC_037110:47.875.406-47.879.660
APHSLHRHCIATQGTLYPKNIQLVMIDDVIVYYYNSSAEQEAVVPEWLNHPEGIEFWQEV
HRNLKFNRYVMDTAVRVTSEHYNHSHDHFYQAHGRCGWKSDGTTEAFMSHAYDGKDFVSF
DVSTRTWTAAVSHAVFYKRKRETDLEDLVRLVIHYESGCIRWLEKLLEFSVTVREPKVPA
VSLFERPPHGNSKVEVTCHVTGFYPRAVQVDWLGAEGLPMVDGVNSGEVLPNGDGSYQLR
KSLTVPQEAQDTQSYSCLVLHSSIAGNITVTWAPKKNLANVLMAIVIIVSVVLMLTVLFK
YLVWRRAVGKS
>Onts-SBAps no prediction chr.13 NC_037109:70.437.457-70.441.973 pseudogene
HCIATQWILNPKLVHSVMIDGVIVYLYNSSGKQEAVMPEWLNHPEGIEFWRDVNRNLNCN
RYVMDTAVRVTSEHYNH*HRWLDYAWRSRSPSCVCY*LKRPTDDLHLGGYVTGFYPQAVQ
VEWLGAQGLPLVDRVSSGEVQPNGDGSYQLRKSPSVPQGSQDTQRYSC
>Onmy-SAA XP_021437178.1 chr.24 NC_035100:28.955.775-28.958.794
MITTILISFMQFSIVAPHSLHRHCIATQGTLYPKNIQLVMIDDVIVYYYNSSAEQEAVVP
EWLNHPEGIEFWQEVHRNLKFNRYVMDTAVRVTSEHYNHSHDHFYQAHGRCGWKSDGTTE
AFMSHAYDGKDFVSFDVSTRTWTAAVSHAVFYKRKRETDLEDLVRLVIHYESGCIRWLEK
LLEFSVTVREPKVPAVSLFERPPHGNSEVEVTCHVTGFYPRAVQVEWLGAEGLPMVDGVN
SGEVLPNGDGSYQLRKSLTVPQEAQDTQSYSCLVLHSSIAGNITVTWAPKKNLANVLMAI
VIIVSVVLILTVLFKYLVWRRAVAQNPR
>Onmy-SBAps no orf predicted chr.27 NC_035103:34.982.134-34.984.099 pseudogene
HCIATQWILNPKLVHSVMIDDVIVYSYNSSGKQEAVMPEWLNHPEGIEFWWDVNRNLNCN
RYVMDTAVRVTSEHYNH*HYVTGFYPQAVQVEWLGAQGLPLVDRVSSGEVLPNGDSTEEE
PECPTGIPGHPEIQLLHSGVARNITVT

>Satr-SAA XP_029629540.1 chr.13 NC_042969.1:77.567.602-77.570.818
MITTILISFMQFSIVAPHSLHRHCIATQGTLYPKNIQLVMIDDVTVYYYNSSAEQEAVVP
EWLNHPEGIEFWQEVHRNLKFNRYVMDTAVRVTSEHYNHSHDHFYQAHGRCGWKSDGTTE
AFMSHAYDGKDFVSFDVSTRTWTAAGSHAVFYKRKRETDLEDLVRLVIHYESGCIRWLEK
LLQLSVTFRKPKVPAVSLFERPPHGNSEVEVTCHVTGFYPRAVQVEWLGAEGLPMVDGVS
SGEVLPNGDGSYQLRKSLTVPQEAQDTQSYSCLVLHSSIAENITLTWAPKKNLANVLMAI
VIIVSVVLILTVLFKYLVWRRAVA
>Satr-SAA2pt XP_029629541.1 chr.13 NC_042969.1:77.576.696-77.578.445
MQVEWLGAEGLPMVDGVSSGEVLPNGDGSYQLRKSLTVPQEAQDTQSYSCLVLHSSVAGN
ITLTWVPKTRSSFWRDRIVTVIGC
>Satr-SBAps no prediction chr.26 NC_042982:33.079.931-33.082.983 pseudogene
HCIATQWILNPKLFQLVMIDDVIVY*YNSGAEQRAVMPEWLNHPEGIEFWRDVNRNLNCN
RYVMDTAVRVTSEHYNH*HHVTGFYPQAVQVEWLGAPGLPLVDRVSRGEVLPSGDGSYQL
GSQDTQRYSCQVVHSSVARITVTW
>Sasa-SAA1 XP_014039590.1 NW_012348808:12.841-15.855
MITTILISFMQFSIVAPHSLHRHCIATQGTLYPKNIQLVMIDDVTVYYYNSSAEQEAVVP
EWLNHPEGIEFWQEVNRNLKFNRFVMDTAVRVTSEHYNHSHDHFYQAHGRCGWKSDGTTE
AFMSHAYDGKDFVSFDVSTRTWTAAVSHAVFYKRKRETDLEDLVRLVIHYESGCIRWLKK
LLQFSVTFREPKVPAVSLFERPPHGNSEVEVTCHVTGFYPRAVQVEWLGAEGLPMVDGVS
SGEVLPNGDGSYQLRKSLTVPQEAQDTQSYSCLVLHSSIAGNITVTWAPKKNLANVLMAI
VIIVSVVLILTVLFKYLVRRRAVGKSQS
>Sasa-SAA2 XP_014044169.1 NW_012366222:207-2.911
APHSLHRHCIATQGTLYPKNIQLVMIDDVTVYYYNSSAEQEAVVPEWLNHPEGIEFWQEV
NRNLKFNRFVMDTAVRVTSEHYNHSHDHFYQAHGRCGWKSDGTTEAFMSHAYDGKDFVSF
DVSTRTWTAAVSHAVFYKRKRETDLEDLVRLVIHYESGCIRWLKKLLQFSVTFREPKVPA
VSLFERPPHGNSEVEVTCHVTGFYPRAVQVEWLGAEGLPMVDGVSSGEVLPNGDGSYQLR
KSLTVPQEAQDTQSYSCLVLHSSIAGNITVTWAPKKNLANVLMAIVIIVSVVLILTVLFK
YLVRRRAVGKSQS
>Sasa-SAA3pt XP_014044525.1 NW_012370682:1.054-1.564
VPAVSLFERPPHGNSEVEVTCHVTGFYPRAVQVEWLGAEGLPMVDGVSSGEVLPNGDGSY
QLRKSLTVPQEAQDTQSYSCLVLHSSIAGNITVTWAPKKNLANVLMAIVIIVSVVLILTV
LFKYLVRRRAVGKSQS
>Sasa-SAA4 XP_014043725.1 NW_012363096:2.586-5.597
MITTILISFMQFSIVAPHSLHRHCIATQGTLYPKNIQLVMIDDVTVYYYNSSAEQEAVVP
EWLNHPEGIEFWQEVNRNLKFNRFVMDTAVRVTSEHYNHSHDHFYQAHGRCGWKSDGTTE
AFMSHAYDGKDFVSFDVSTRTWTAAVSHAVFYKRKRETDLEDLVRLVIHYESGCIRWLKK
LLQFSVTFREPKVPAVSLFERPPHGNSEVEVTCHVTGFYPRAVQVEWLGAEGLPMVDGVS
SGEVLPNGDGSYQLRKSLTVPQEAQDTQSYSCLVLHSSVAGNITLTWAPKKNLANVLMAI
VIIVSVVLILTVLFKYLVRRRAVGKSQS
>Sasa-SAA5pt XR_001327798.1 NW_012573932:108-952
APHSLHRHCIATQGTLYPKNIQLVMIDDVTVYYYNSSAEQEAVVPEWLNHPEGIEFWQEV
NRNLKFNRFVMDTAVRVTSEHYNHSHDHFYQAHGRCGWKSDGTTKAFMSHAYDGKDFVSF
>Sasa-SAA6pt XR_001326745.1 NW_012361613:51-929
APHSLHRHCIATQGTLYPKNIQLVMIDDVTVYYYNSSAEQEAVVPEWLNHPEGIEFWQEV
NRNLKFNRFVMDTAVRVTSEHYNHSHDHFYQAHGRCGWKSDGTTEAFMSHAYDGKDFVSF
DVSTRTWTAAVS
>Eslu-SAA XP_010891190.1 chr.01 NC_025968.4:29.672.471-29.675.989
MITTLVVLFLQLSIVDPHTFHRHCIASQGTLYPKNLQIVMVDDVMVYYYNSSAEQDAIIP
EWMDHSEGIEFWREMHQNLKYNRFVMDSAVKMTSEHFNHSHDHIYQAHGRCGWRSDGSID
ASMSHAYDGKDFVSFDVQTRTWTAAVSHAAFYKRKRETDLEDLVRLVIHYESGCIRWLEN
LLKFSIKVREPKVPEVIVFERLPHGSSEVEVTCHVTGFYPRAVQVEWIGSEGLPLVDRVI
SGEVLPNGDGSYQLRKSLTVLQGVQYNQSYTCLVHHSSVTGSVSVTWVPKKNLNYMVIAV
CIIVAVTFLFILAVLYKAGVIGHFR

AF4.5 Deduced H lineage amino acid sequences

>Saal-HAA XP_023833862.1 chr.32 NC_036871:14.212.450-14.221.846
MRIPLTFILFFKLSWGLPIDTVILFQRSGVVGEMETVEQVLVNGAPLSNCGKYVSGILRA
VLLGNYSESFQQAFEIEENVTNTMTKNPTYQYLRVRECKLNGFRVVHLTDQLLVNGNDFL
TLDQSTDTWTAEVPQALSLKQLWDWDTERTRRERMQLHESCTELMELTQSEPTTNGAGTS
LLTVLAPLFAGLAFVAVVIASFLISQQDPKVSGHPGGVLGSIVHYPQNVTETPQAPQSGK

```
XNQVL
>Saal-HBA chr.19 XP_023865004.1 NC_036859:27.504.960-27.509.583
MRLPLTFILICELCWGFKRDMVIQFQRSGVVGEVEQVLINGAALFSCEKDVNGILRAVLL
GNYSESFQQAFEIEENVNNTMTKNHTYQYLRVHESKLDGFRVVHLTXRLLVDGNYFLTLD
RNTDTWTAKVPQALALKQLWDQDTEGMRLERIQLHESCAKLMKELKHSEPTTTGGGMSML
AALASLLAGLAFVALVTTSFLIFKQQDPNVSGHPGSVLGSIVHYPQNVTETPRPGKGYQV
L
>Onne-HAA XP_029498320.1 chr.9b NC_042563:14.569.407-14.582.882
MLLPLTFILFFNLSWGLPIDTVILFQRSGVVGEMETVEQVLVNGAPPSSSGKNVSGILRA
VLMGNYESFHQAFEIEEVTNTMTSREKPTYQYLRVRECKLNGFRVVHSTDQLLVNENDFL
TLDQSTDTWTAEVPQALSLKQLWDWDTEFTRREKMQLHEICTELMKELRQSEPTTNRGTS
LLTVLAPLFACLTFVVMVIASFLISKQQDPRVSRHPGGVLGSIVHYYQNVTETQSGKDYQ
GPLKLNPPPLIHL
>Onne-HBA XP_029479749.1 chr.20 NC_042554:60.651.094-60.662.234
MRLPLTFILICELCWGFKRDMVIQFQRSGVVGEVEQVLINGAALSSCEKDVNGILRAVLL
GNYSESFQQAFEIEEHVNNTMTKNHTYQYLRVHESKLDGFRVVHLTDRLLVDGNDFLTLD
RNTDTWTAEVPQALALKHLWDQDTERTRLERIQLHESCAKLMKELTHSEPITTGGGMSML
AALASLLAGLAFVALVTTSFLIFKQQDPNVSGHPGGVLGSIVHYPQNVTETPCPGKGYQV
L
>Onki-HAA XP_020322949.1 chr.30 NC_034203:14.721.044-14.737.299
MLLPLTFILFFNISWGLPIDTVILFQRSGVVGEMETVEQVLVNGAPPSSGGKNVSGILRA
VLMGNYESFQQAFEIEENVSNTMTKNPTYQYLRVRECKLNGFRVVHSTDQLLVNGNYFLT
LDQSTDTWTAEVPQALSLKRLWDWDTEFTRREKMQLHESCTELMKELRHSEPTTNRGTSL
LTVLASLFACLTFVVMVIASFLMSKQQDPRVSRHPGGVLGSIVHYYQNVTETQGGKDY
>Onki-HBA XP_031663205.1 chr.27 NC_034200:28.309.378-28.315.392
MRLPLTFILICELCWGFKRDMVIQFQRSGVVGEVEQVLINGAALSSCEKDVNGILRAVLL
GNYSESFQQAFEIEENVNNTMTKNHTYQYLRVHESKLDGFRVVHLTDRLLVDGNDFLTLD
RNTDTWTAEVPQALALKHLWDQDTERTRLERIQLHESCAKRMKELTHSEPITTGVPCLTS
YEAGESGEFLLCFQGGMSMLAALASLLAGLALVAMVTTSFLIFKQQDPNVSGHPGGVLGS
IVHYPQNVTEKPCPGKGYQVL
>Onts-HAA XP_024291244.1 chr.10 NC_037106:35.188.051-35.172.011
MLLPLTFILFFNLSWGLPIDTVILFQRSGVVGEMETVEQVLVNGAPPSSGGKNVSGILRA
VLMGNYESFQQAFEIEENVTNTMTKNPTYQYLRVRECKLNGFRVVHSTDQLLVNGNDFLT
LDQSTDTWTAEVPQALSLKQLWDWDTEFTRREKMQLHESCTELMKELRHSEPTTNRGTSL
LTVLAPLFACLTFVVMVIASFLMSKQQDPRVSRHPGGVLGSIVHYYQNVTETQSGKDYQA
>Onts-HBA XP_024247562.1 chr.28 NC_037124:27.560.431-27.566.291
MRLPLTFILICELCWGLKRDMVIQFQRSGVVGEVEQVLINGAALSSCEKDVNGILRAVLL
GNYSESFQQAFEIEENVNNTMTKNHTYQYLRVHESKLDGFRVVHLTDRLLVDGNDFLTLD
RNTDTWTAEVPQALALKHLWDQDTERTRLERIQLHESCAKRMKELTHSEPITTGVPCLTS
YEAGESGEFLLCFQGGMSMLAALASLLAGLAFVAMVTTSFLIFKQQDPNISGHPGGVLGS
IVHYPQNVTETPCPGKGYQVL
>Onmy-HAA XP_021468778.1 chr.08 NC_035084:52.834.866-52.853.052
MQLPLTFILFFNLSWGLPIDTVILFQRSGVVGEMETVEQVLVNGAPLSGSGKYVSGILRA
VLMGNYESFQQAFEIEENVTNTMTKNPTYQYLRVRECKLNGFRVVHSTDQLLVNGNDFLT
LDQSTDTWTAEVPQALSLKQLWDWDTEFTRREKMQLHESCIELMKELRHSEPTTNRGTSL
LTVLAPLFACLTFVVMVIASFLISKQRDPRVSRHPGGVLGSIVHYYQNVTETQSGKDYQA
L
>Onmy-HBA XR_002471367.1 chr.28 NC_035104:26.645.362-26.651.156
MRLPLTFILICELCWGFKVDMVIQFQRSGVVGEVEQVLINGAALSSCEKDVNGILRAVLL
GNYSESFQQAFEIEENVNNTMTSRPHESKLDGFPVVHLTDRLLVDGNDFLTLDRNTDTWT
AEVPQALALKHLWDQDTERTRLERIQLHESCAKLMKELTHSEPITPAGVLGSIVHYPQNV
TETPCPGKGYQVL
>Satr-HAA XP_029560449.1 chr.21 NC_042977: 17.473.705-17.488.093
MRLPLTFILFFKLNWGLPIDTVILFQRSGVVGEMETVEQVLVNGAPLSSCGKYVSGILRA
VLLGNDSESFQQAFEIEENVTNTITKNPTYQYLRVRECKLNGFRVVHLTDQLLVNGNYFL
TLDQSTDTWTAEVPQALALKQLWDWDTERTRRERMQLHESCTELMKELTHSEPTTNREMS
LLTVLAPLLASLAFVAVVIASFLIANRQDPRVSGHPGGVLGSIVHYPQNVTETPQAPQSG
KDYQVL
>Satr-HBAps no prediction chr.31 NC_042987:27.983.152-27.989.933 pseudogene
VDMVIQFQRSEVVGEVETVEQGLISGAALSSCEKDVSGILRAVLLGNYSESFQQAFEIEE
NVNNTMTENHTYQYLRVHESKLDGFQVVHLTDQLLVDGNDFLTLDRNTDTWTAEVPQALA
LKQL*DQDTERTRLERIQLHESCAKLMKELTHSEPTTPAGVLGSIVHYPQNVTETPRPGK
GYQVL
>Sasa-HAA XP_013995094.1 chr.14 NC_027313.1:16.479.536-16.491.787
```

MRLPLTFILFFKLNWGLPIDTVILFQRSGVVGEMDTVEQVLVNGAPLSSCGKYVSGILRA
VLLGNDSESFQQAFEMEENVTNTITKNPTYQYLRVRECKLNGFRVVHLTDQLLVNGSDFL
TLDQSTVTWTAEVPQALALKQLWDWDTERTRRERMQLHESCTELMKELTHSEPTTNRAGM
SLLTVLAPLLASLAFVAVVIASFLIANRQDPRVSGHPGGVLGSIVHYPQNVTETPQAPQS
GKDYQVL
>Sasa-HBAps chr.03_NC_027302.1:17.030.685-17.037.352 pseudogene
VDMVIQFQRSGVVGEVKTVEQVLISGAALSSSEKDVSGILRAVLLGNYSESFQQAFEIEE
NVNNTMTENHTYQYLRVHESKLDGFQVVHLTDQLLVDRNDFLTLDRNTDTWTAEVPQALA
LKQL*DQDTERTRLDRIQLHESCAKLMKEITHSEPTTPAGVLGSIVHYPQNVTETPRPGK
GYRVL
>Eslu-HAA XP_010881876.1 chr.03 NC_025970:32.751.310-32.757.904
MIANMQLPLITILLFKSCLCLQIDTVIQFLRSGVFGKVETVEQVLVNGAPLSSCGKDVSS
ILNAVLLINYNESFQTIEESGNNTMKISHTYMRVRECKLDSFGVLLLTDRLLVDGNNFLT
LDRATDTWTAEGPQALPLKHLWDRESTRTRREIMQLHESCTKLLEEITCSEPGATGGMDI
PTALAPLLAGLGFVILVIASFLISKKQDPTISGHPGGVIGSIIHYPHNISELSHSPNPGR
GYQVL

AF4.6 Deduced P lineage amino acid sequences

>Saal-PAApt No prediction chr.03 NC_036840:4.724.783-4.724.090
VLQRRRGCTYITNESSTGFDQWGLNGENFLTFDPTSHTWMSESPQANPIEPSWNSNKVRS
HVFKEFLQHDIPHKEDVSLRCHGTSTDLRKVHLTRDRVVMTDRVRVIGPLPNVDGSVLLR
L
>Onne-PAAps No prediction chr.08 NC_042542:3.503.547-3.504.250 pseudogene
VLQRRRGCTYITNESSAGFDQWGLNGENFLTFDPTSHTWMSESLQANPIEQSWISNKVRS
HMFKEFLQDDLPHKEDVSLTCHVTSTDLSGLKVHLTRDRDVMTDRVRVIGPLPNVAGSFL
LRLSVEIPTGLTKT*RYRCKVQTSTSNTAA
>Onki-PAAps No prediction chr.09 NC_034182:5.596.843-5.596.152 pseudogene
VLQRRRGCTYITNESSAGFDQWLNGENFLTFDPTSHTWMSESLQANPIEQSWKSNKVRSH
MFKEFLQDDLPHKEDVSLRCYVTSTDLKVHLTRDRDVMTDRVRVIGPLPNVAGSFLLRLS
VEIPTGLTKT*RYRCKVQTSTSNTAA
>Onts-PAAps No prediction chr.15 NC_037111:3.386.312-3.386.578 pseudogene
SMIFKFAKPIPHKEDVSLRCYVTSTDLSGLKVHLTRDRDVMTDRVRVIGPLPNVAGSFLL
RLSVEIPTGLTKT*RYRCKLQTSTSNTAA
>Onmy-PAAps No prediction chr.21 NC_035097:14.167.986-14.168.685 pseudogene
VLQRRRGCTYITNESSAGFDQWGLIGEN*LTFDPTSHTWMSESLQANPIEQSWNSNKVRS
HLFKEFLQDDLPHKLDVSLRCHVTSTDLSGLKVHLTRDRDVMTDRVRVIGPLPNVDGSVL
LRLSVEIPTGLTKT*RYRCKVQTSTSNTAA
>Satr-PAAps No prediction chr.08 NC_042964:41.135.886-41.136.011 pseudogene
IGPLPNVDGSVLLRLSVEIPTGHTKS*RYRCKVQTSTSNTAA
>Sasa-PAAps No prediction chr.07 NC_027306.1:43.236.079-43.236.783 pseudogene
VLQRRRGCTYITN*SSTGFGQ*GLNGENFLTFDPTSHTWMSESLQANPIEQSWNSNKVRS
HMFKEFLQHDCPH*EDVSLRCHVTSTDLSGLKVHLTRDRGVMTDRARVIGPLPNVDGSVL
LRLSVEIPTGHTKS*RYHCKVQTSTSNTAA
